# GO/APTES-mediated bifunctional CuFe_2_O_4_@GO-NH_2_-facilitated synthesis of pyrazolo-triazepine scaffolds as a potent post-prandial antidiabetic agent against dual α-amylase and α-glucosidase enzymes

**DOI:** 10.1039/d6ra00293e

**Published:** 2026-05-18

**Authors:** Romica Jain, Ashok Kumar, Pratibha Sharma

**Affiliations:** a School of Chemical Sciences, Devi Ahilya University Indore 452001 (M.P.) India drpratibhasharma@yahoo.com

## Abstract

Non-insulin-dependent diabetes mellitus (NIDDM) is a prevalent illness among individuals. This study elucidates the synthesis of pyrazolo-fused triazepine skeletons containing heterocycles and their assay-based *in vitro* and *in silico* antidiabetic activity against dual α-amylase and α-glucosidase enzymes followed by *ab initio* studies. The synthesis was assisted by acid-base bifunctional APTES-grafted magnetic nanocatalyst (CuFe_2_O_4_@GO-NH_2_), using dibenzalacetone (1a–g) and hydrazine (2a–b) as precursors, followed by the addition of isoniazid (3). The obtained heterocyclic compounds (4a–n) were corroborated using spectroscopic techniques. The *ab initio* structural insights were computed at the B3LYP/6-311G++(d,p) level of theory to investigate the HOMO–LUMO energy gap, chemical reactivity and chemical potential of the synthesized compounds. Molecular docking using the CDOCKER (CHARMm-based DOCKER) algorithm revealed appreciable binding interaction modes between the active sites of the receptor (PDB ID: 2QV4 and 3W37) and heterocyclic ligands. Among all the synthesized compounds, 4,4'-(1-(2,4-dinitrophenyl)-8-hydroxy-8-(pyridin-4-yl)-2,3,5,6,7,8-hexahydro-1*H*-pyrazolo-[1,5-*d*][1,2,4]-triazepine-2,5-diyl)bis(2-methoxyphenol) (4d) (IC_50_ = 123.79 µg mL^−1^) exhibited superior antidiabetic activity against α-amylase compared with the existing drug acarbose (IC_50_ = 171.8 µg mL^−1^), which is in accordance with the *in silico* and DFT studies. ADMET, Lipinski's Rule and TOPKAT descriptor studies were performed to assess the remarkable biocompatibility and toxicity. Therefore, the synthesized heterocycles could emerge as potent antidiabetic drugs for first-line treatment in the near future.

## Abstract 2

CuFe_2_O_4_@GO-NH_2_ driven synthesis and multidimensional analysis of pyrazolo-fused triazepine for antidiabetic potential.

## Graphical abstract

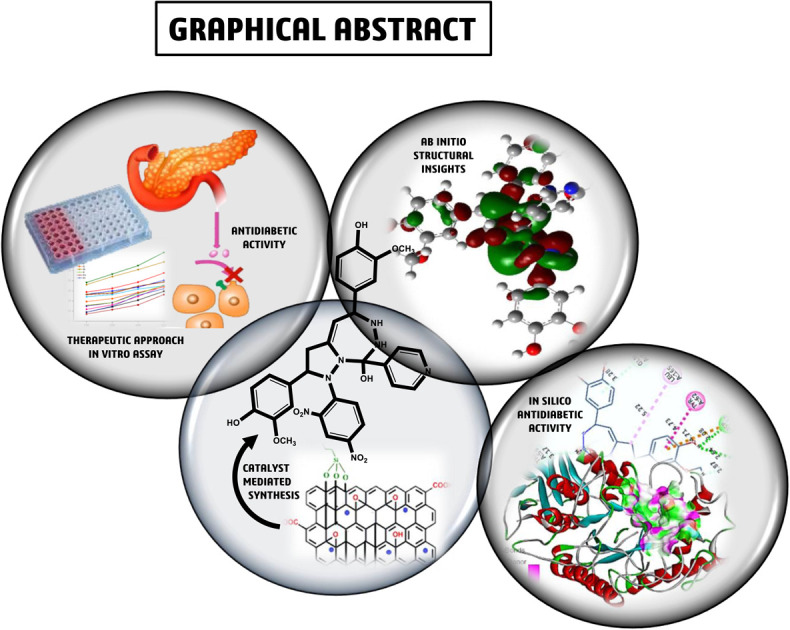

## 1. Introduction

Diabetes mellitus is a serious life-threatening disease affecting a considerable portion of the aged population, as claimed by the International Diabetes Federation (2021),^1^ and is one of the most common consequences of detrimental life habits. Despite the availability of several existing antidiabetic drugs, *viz.*, acarbose, miglitol, and voglibose, many of these are accompanied by adverse side effects, such as nausea, abdominal distension, flatulence, diarrhea, weight loss^2–4^ and low absorption.^5,6^ This has created a paramount need to develop novel drugs with minimal side effects and poor absorption. Diabetes is a condition in which the beta cells of the pancreas do not produce insulin adequately or develop insulin resistance, resulting in high levels of glucose in the blood, termed “hyperglycemia”.^7^ It is evident that inadequate secretion or malfunctioning of insulin is a substantial cause of type-II diabetes mellitus. Glucose primarily enters the blood owing to the hydrolysis of carbohydrates *via* the action of α-amylase and α-glucosidase enzymes, which are responsible for starch breakdown and intestinal absorption, respectively. In addition, α-amylase plays a vital role in the digestion of polysaccharides, such as starch and glycogen, by disintegrating them into monomers by cleaving (1,4) glycosidic linkages,^8^ while α-glucosidase liberates glucose in the blood by cleaving the glycosidic linkages of non-reducing sugars.^9^ The deterioration of these two enzymes has proven to be a significant therapeutic procedure for treating post-prandial hyperglycemia, which is associated with type-II diabetes.^10^

**Fig. 1:**
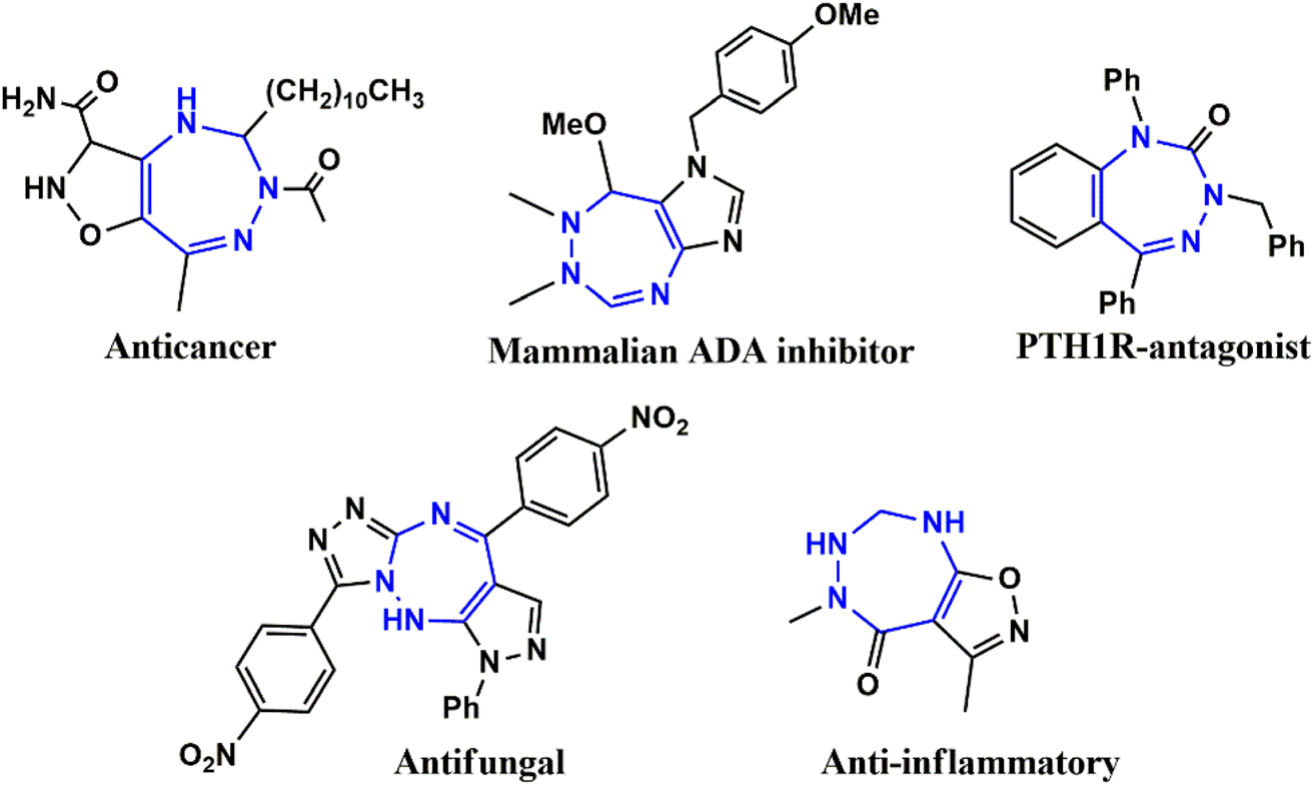
Biologically active heterocycles adopting a [1,2,4]-triazepine framework.

Beyond these observations are the current need and prerequisites for developing a new antidiabetic drug. Virtual screening and *ab initio* studies are the best recommended practices before proceeding directly to *in vitro* analysis. Phytochemicals are verified to possess various inherent biological activities against several diseases and have very few side effects. It was found that most phytochemicals, which are promising pharmacologically active drugs, are mainly composed of heterocyclic rings and that about 67% of the heterocycles are extracted from plants.^11^ Thus, by possessing a significant biological application (Fig. 1), *viz.*, anti-cancer,^12,13^ anti-inflammatory,^14^ and anti-microbial activities,^15^ PTH1R antagonist, mammalian ADA inhibitor,^16^ and antifungal^17^ activity, the^1,2,4^-triazepine skeleton becomes a molecule of biological interest. Therefore, the present study illustrates the synthesis of two novel series of heterocycles comprising pyrazolo-fused triazepine moieties, followed by computational studies comprising molecular docking with the protein (PDB ID: 2QV4 and 3W37), density functional theory (ligand optimization, HOMO–LUMO energy gap, and chemical potential), ADMET and toxicity evaluation studies. Furthermore, the docked ligands with proteins were chosen for assay-based *in vitro* analysis to validate their antidiabetic activities against α-amylase and α-glucosidase enzymes.

To address the growing demands for green and sustainable approaches, the introduction of heterogeneous magnetic catalysts in the cascade-cyclized synthetic protocol has laid the foundation for tailoring heterocycles. In this regard, multi-component one-pot synthesis is also powerful to construct heterocycles *via* cascade cyclization.^11,18–20^ As multiple steps, from condensation to cyclization to rearrangement, occur in one vessel, it enhances the reaction efficiency, overall yield, time and step economy in incorporating heterocycles.^21–23^ By keeping in mind the green synthetic protocols and the need for an efficient antidiabetic drug, we constructed a magnetic nanocatalyst-mediated synthesis of precursor dibenzalacetone (1a–g), which subsequently reacts with hydrazines (2a–b) to form^4,5^-dihydropyrazole intermediate (IIIaa–ag and IIIba–bg, respectively). Furthermore, the addition of isoniazid (3) gave the desired novel drug compounds 1-(2,4-dinitrophenyl)-2,5-diphenyl-8-(pyridin-4-yl)-2,3,5,6,7,8-hexahydro-1*H*-pyrazolo[1,5-*d*][1,2,4]-triazepin-8-ol (4a–g) and 8-hydroxy-2,5-diphenyl-8-(pyridin-4-yl)-2,3,5,6,7,8-hexahydro-1*H*-pyrazolo-[1,5-*d*][1,2,4]-triazepine-1-carboxamide (4h–n).

Several reported reactions have used different catalysts, like GO, ZnO/r-GO, Al_2_O_3_/CaO, and ZnO NPs, to synthesize dibenzalacetone.^24,25^ However, due to various bottlenecks with existing protocols, *viz.*, low yield, harsh conditions, and tedious workup, we performed the synthesis using a novel bifunctional catalyst. The versatile and compelling properties of acid-modified graphene, *i.e.* GO, such as low toxicity, catalytic activity, structural dispersion in polar solvents, large surface area, and easy separation,^26–30^ make it an acceptable catalyst for many organic conversions.^31^ To address issues like agglomeration and separation after the completion of the chemical reaction, GO is immobilized by magnetic CuFe_2_O_4_ nanoparticles that exhibit catalytic applications^32^ and provide a large surface area,^33^ as Fe^3+^ in copper ferrite also acts as a Lewis acid.^34^ Furthermore, to append the basic nature in as-prepared CuFe_2_O_4_@GO, (3-aminopropyl)triethoxysilane (APTES) was grafted on its surface for amine functionalization, resulting in the desired bifunctional acid-base catalytic activity (CuFe_2_O_4_@GO-NH_2_).

Thus, this study aims to establish a one-pot, magnetic nanocatalyst CuFe_2_O_4_@GO-NH_2_-mediated synthesis of novel antidiabetic pyrazolo-fused triazepine (4a–n) using multi-component-synthesized precursor dibenzalacetone (1a–g), hydrazines (2a–b) and isoniazid (3), followed by the evaluation of their biological activities. A greater extent of red region in the surface contour of 4d and 4k exposes the compound susceptibility towards electrophile, showing efficient hydrogen bonding, which again demonstrates good agreement with biological activity.

## 2. Results and discussion

### 2.1. Chemistry

#### 2.1.1. Magnetic nanocatalyst CuFe_2_O_4_@GO-NH_2_

**Scheme 1:**
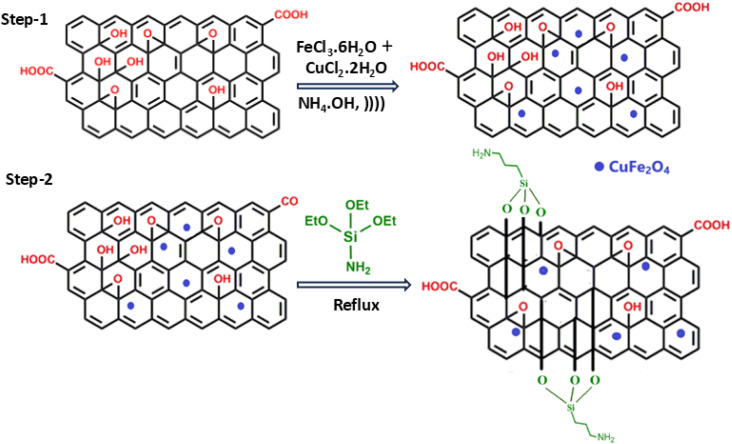
Fabrication of CuFe_2_O_4_@GO-NH_2_. )))) represent ultrasonics irradiation.

The acidic functionalization of graphene to tailor GO was carried out under ulrasonic irradiation, as reported in literature. Subsequently, CuFe_2_O_4_@GO-NH_2_ was synthesized by the sequential addition of copper and iron salts to GO under ultrasonic irradiations, followed by the addition of APTES refluxing, with anhydrous toluene for amine functionalization (Scheme 1). Anhydrous toluene was necessary to enhance refluxing and avoid hydrolysis and premature polymerization. The dark brown solid nanocatalyst thus prepared was characterized using different analytical techniques.

##### 2.1.1.1 FT-IR

**Fig. 2:**
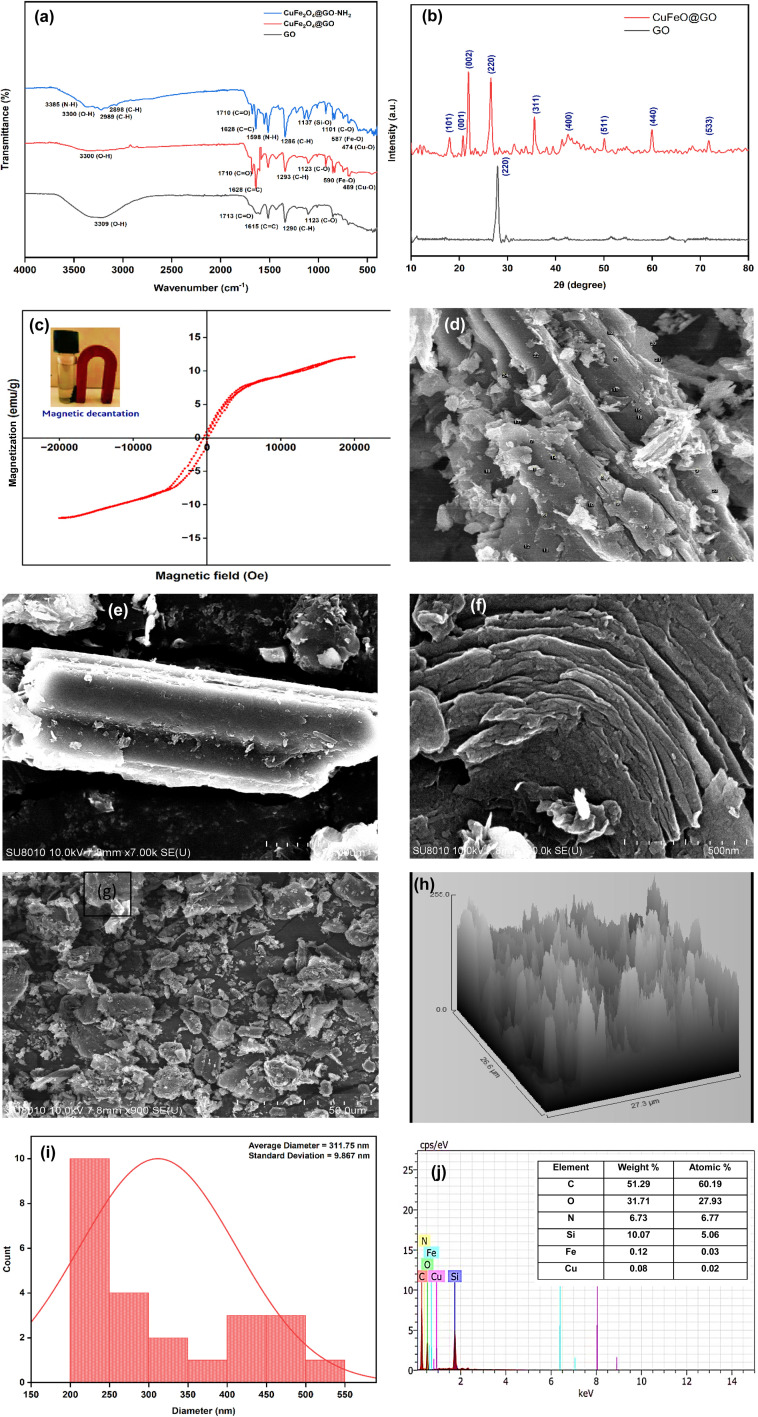
Characterization of CuFe_2_O_4_@GO-NH_2_: FT-IR spectra (a), XRD patterns (b), hysteresis loop (c), FE-SEM images (d)–(g), surface roughness plot (h), particle size distribution curve (i), and EDX elemental mapping (j).

In FT-IR, the peaks near 3385 cm^−1^, 1598 cm^−1^ and 3300 cm^−1^ are associated with N–H stretching, bending vibrations and O–H stretching vibration, respectively. The bands around 1710 cm^−1^(C=O), 1628 cm^−1^(C=C), 1137 cm^−1^(Si–O), 587 cm^−1^(Fe–O), and 474 cm^−1^(Cu–O) confirm the successful functionalization of GO.^35,36^ The frequency shifts and intensity changes in functional group bands (compared with bare GO) further depict the chemical interaction among CuFe_2_O_4_, APTES and GO (Fig. 2a).

##### 2.1.1.2 XRD

The high intense peak at 26.5° with the (002) index and a *d*-spacing of 3.36 Å of represents graphene. A single phase cubic spinel structure of CuFe_2_O_4_@GO-NH_2_ with 2*θ* values at 18.01°, 30.2°, 35.6°, 43.3°, 51.6°, 62.09°, and 73.68° mapped to (101), (220), (311), (400), (511), (440), and (533), respectively^37^ (Fig. 2b). An average crystalline size of 45 nm was obtained and determined by applying the Debye Scherrer method. Low diffraction angles at 20.85° (*d*-spacing = 4.25 Å) and 21.74° (*d*-spacing = 4.07 Å) with expansion factors of 1.26 and 1.21, respectively, reveal the interlayer (001) amination, oxygen insertion, and intercalated water molecules that push the sheets far apart compared with pristine graphite.

##### 2.1.1.3 Magnetism

The observed magnetic profile with magnetic saturation of 12.096 emu g^−1^ was performed from −20 kOe to +20 kOe using a vibrating sample magnetometer (Fig. 2c). Due to the addition of non-magnetic GO-NH_2_, magnetic saturation is low compared with copper ferrite, but it is commendatory for magnetic separation of the catalyst from the reaction mixture.

##### 2.1.1.4 FE-SEM and EDX

In Fig. 2d–f, the faint contrast regions in FE-SEM images depicted the less flat, smooth ultrathin and wrinkled support GO sheets due to the presence of functional groups and embedded strong contrast spherical CuFe_2_O_4_, which made the GO surface rough (Fig. 2g). Due to the presence of mild agglomeration of CuFe_2_O_4_ and organic residues, the size increases to 311.75 nm (Fig. 2h). The elemental composition of C, N, O, Fe, Cu, and Si was calculated through EDX (Fig. 2i), favoring the structural design of the catalyst.

#### 2.1.2. Heterocycles

**Scheme 2:**
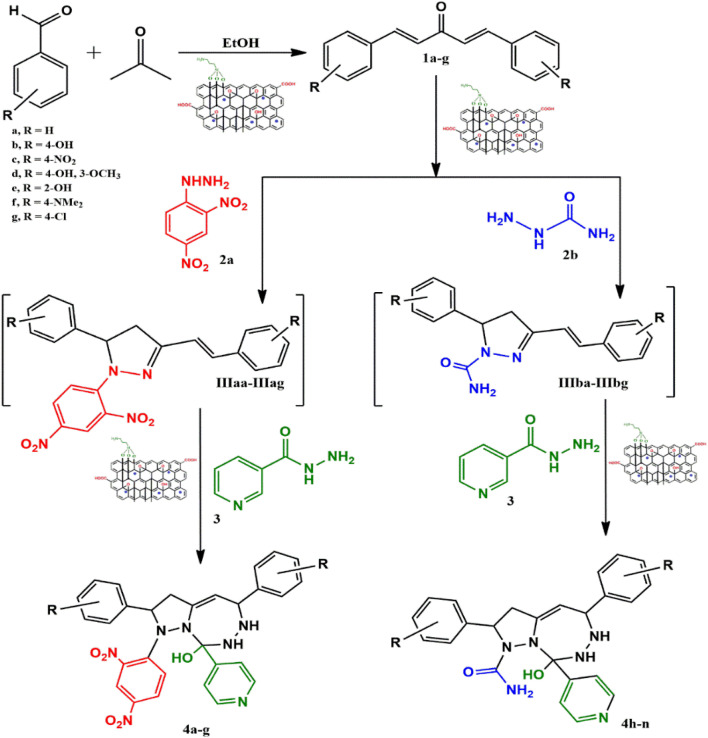
Synthesis of the heterocycles (4a–n).

**Scheme 3:**
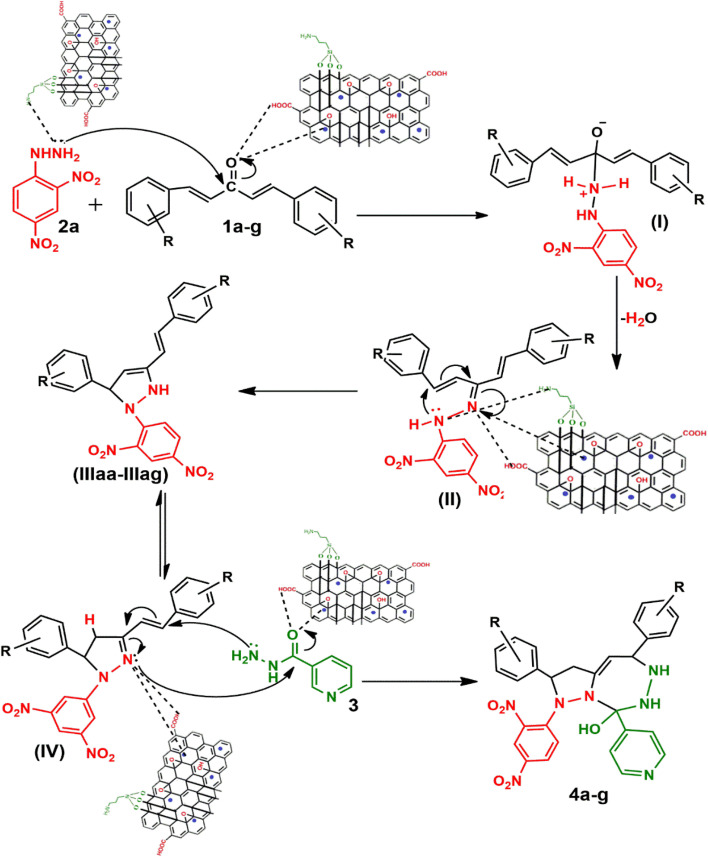
Plausible mechanism of the synthesis of heterocycles (4a–g).

A catalyst-mediated synthetic protocol was developed to en route the target compounds in three steps, as shown in Scheme 2. CuFe_2_O_4_@GO-NH_2_-catalyzed Claisen–Schmidt condensation was established between aromatic aldehydes and acetone in the presence of ethanol to construct a dibenzalacetone analogue (1a–g) (Table 1). With 0.04 g catalyst loading, 1c yielded 85% without use of sodium hydroxide, which has traditionally been used^38,39^ for this condensation reaction (Table 2). Among the solvents studied, ethanol exhibited superior results due to its moderate polarity, which is not as high as that of water and as low as that of acetonitrile, good solubility of reactants, support for ionization, lack of suppression of the base activity of the catalyst, and stabilization of the enolate intermediate and transition states. It was observed that benzaldehydes with electron-withdrawing substituents produce a maximum yield of 85% (1c), while electron-donating substituents show low returns. The synthesized dibenzalacetone (1a–g) was used as a substrate in the next step, reacted with hydrazine (2a–b) in the presence of catalyst CuFe_2_O_4_@GO-NH_2_ in ethanol, and was heated to reflux for 3–4 h at 70 °C to incorporate the^4,5^ dihydropyrazole intermediate (IIIaa–IIIag and IIIba–IIIbg) (Fig. S44). An electron withdrawing group attached to hydrazine decreases the nucleophilicity of nitrogen, which can be enhanced by deprotonation in a basic medium.^40^ Thus, the acidic and basic functionality of the catalyst plays an important role in the activation of the carbonyl of dibenzalacetone and the nitrogen of hydrazine, respectively. It was found that the corresponding hydrazones (Fig. S46) were obtained at a lower temperature around 40 °C, so to achieve the desired cyclized pyrazoline, a high temperature was required. Now, without isolating the cyclized intermediate and catalyst, isoniazid (3) was added along with ethanol, and after continuous stirring of 3–4 h under reflux, the desired fused triazepine (4a–n) was obtained (Tables 3 and 4). With 0.02 g catalyst and 3 hours of refluxing, 4b and 4i in ethanol solvent produce maximum yields of 65% and 63%, respectively (Tables 5 and 6). Ethanol was found to be the best medium due to its tendency to form hydrogen bonding and sufficient dipole–dipole interaction, which activates carbonyl carbon electrophilicity and stabilizes imine, cyclized intermediates and transition states, with subsequent suppression of side products, and due to moderate Lewis acidity, it does not over-stabilize the nucleophile, like strongly polar solvents. One major spot associated with the desired product was observed along with several other spots in the TLC using the hexane:ethyl acetate (7 : 3) eluent, and the compound was purified *via* column chromatography without trying to isolate and identify other spots. To avoid side products, lower temperatures and low catalyst loading were favorable, but in the absence of a catalyst, no product was obtained. However, the presence of electron donating substituents on the benzene ring underwent efficient cascade ring closure *via* attack of pyrazoline nitrogen to carbonyl carbon of isoniazid, facilitated by a catalyst (Scheme 3), and the ortho-substituted benzene ring offered poor yield due to steric crowding during cyclization. When we performed the reaction with GO and CuFe_2_O_4_@GO, we did not obtain the final desired product as the reaction can occur with the synthesized catalyst because the enhanced catalytic activity of CuFe_2_O_4_@GO-NH_2_ is attributed to the synergistic interaction between Lewis acidic Cu^2+^/Fe^3+^ centers, Brønsted acidic sites –COOH, –OH and basic –NH_2_ groups, which enables simultaneous activation of electrophile and nucleophile, thereby facilitating efficient Schiff base formation and subsequent cyclization. An additional key feature of the synthesized magnetic CuFe_2_O_4_@GO-NH_2_ that puts the reaction protocol under the umbrella of green chemistry is the facile recovery of the catalyst *via* external magnet, and it can be reused up to five cycles with a subsequent decrease in yield to 25–30% in the fifth cycle for all the synthesized heterocycles (4a–n). The heterogeneity of CuFe_2_O_4_@GO-NH_2_ was confirmed by performing a hot filtration test^40^ to tailor 4b and 4i. After 1.5 hours, when half of the conversion had taken place, the catalyst was removed from the reaction mixture, and the reaction mixture was continuously stirred for the next 1.5 hours. It was found with the help of TLC that no progression and completion of reaction occurred for both 4b and 4i, hence clearly revealing that no leaching of catalyst took place, which indicates its heterogeneity. The structures of the compounds were elucidated by ^1^H and ^13^C NMR spectra.

**Table 1:** Reaction-optimized conditions for the synthesis of 1a–g^a^^,^^d^

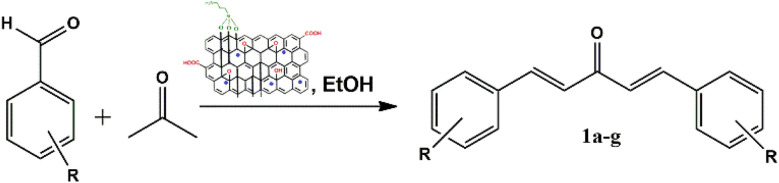					
Entry	Benzaldehydes	Temp (in °C)	Time^c^ (h)	Product	Yield^b^ (in %)
1	R = H	r.t.	3	1a	80
2	R = 4-OH	40	4.5	1b	40
3	R = 4-NO_2_	r.t.	3	1c	85
4	R = 4-OH and 3-OCH_3_	40	4	1d	55
5	R = 2-OH	40	5	1e	30
6	R = 4-NMe2	50	4.5	1f	35
7	R = 4-Cl	r.t.	3	1g	83

a Reaction conditions: benzaldehydes (2 mmol), acetone (1 mmol), solvent (10 mL), and r.t. 25 °C-30 °C.

b Isolated yield.

c All the reactions were monitored using TLC.

d Catalyst loading = 40 mg.

**Table 2:** Optimized reaction conditions for the synthesis of 1c^a^

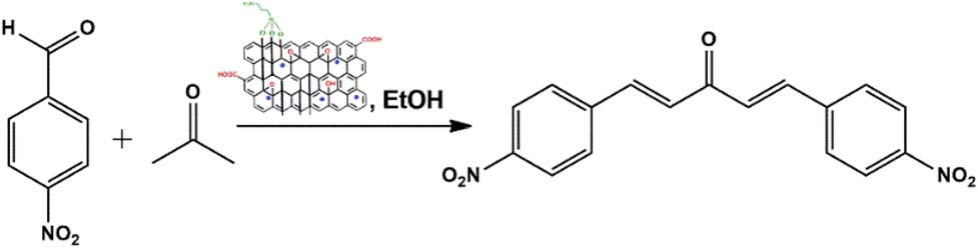					
Entry	Catalyst loading (mg)	Solvent	Temp (in °C)	Time^c^ (h)	Yield^b^ (in %)
1	20 (CuFe_2_O_4_@GO-NH_2_)	CH_3_CN	40	3	No product
2	30 (CuFe_2_O_4_@GO-NH_2_)	CH_3_CN	Reflux	4	No product
3	20 (CuFe_2_O_4_@GO-NH_2_)	H_2_O	40	2	20
4	40 (CuFe_2_O_4_@GO-NH_2_)	H_2_O	Reflux	2	28
5	20 (CuFe_2_O_4_@GO-NH_2_)	EtOH	r.t.	2	35
6	20 (CuFe_2_O_4_@GO-NH_2_)	EtOH	Reflux	2	40
7	30 (CuFe_2_O_4_@GO-NH_2_)	EtOH	r.t.	2.5	70
8	40 (CuFe_2_O_4_@GO-NH_2_)	EtOH	r.t.	3	85
9	40 (CuFe_2_O_4_@GO-NH_2_)	DCM	r.t.	3	No product
10	40 (CuFe_2_O_4_@GO-NH_2_)	EtOH + H_2_O (6 : 4)	r.t.	3	55
11	40 (CuFe_2_O_4_@GO-NH_2_)	EtOH + H_2_O (8 : 2)	r.t.	3	65
12	40 (CuFe_2_O_4_@GO)	EtOH	60	4	70
13	60 (GO)	EtOH	70	4.5	65
14	60 (CuFe_2_O_4_)	EtOH	60	4	60

a Reaction conditions: *p*-nitrobenzaldehyde (2 mmol), acetone (1 mmol), solvent (10 mL), and r.t. 25 °C-30 °C.

b Isolated yield.

c All the reactions were monitored by TLC.

**Table 3:** Optimized reaction conditions for the synthesis of 4a–g^a^

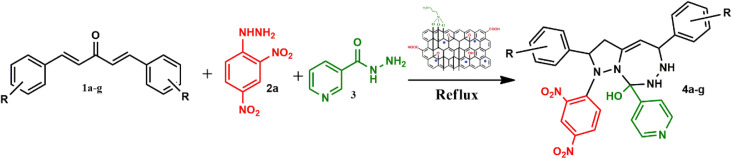						
Entry	Dibenzalacetone	R	Temp^b^ (in °C)	Time^c^ (h)	Product	Yield^d^ (in %)
1	1a	R =H	40	3	4a	53
2	1b	R = 4-OH	40	3	4b	65
3	1c	R = 4-NO_2_	40	3.5	4c	43
4	1d	R = 4-OH and 3-OCH_3_	45	4	4d	55
5	1e	R = 2-OH	45	4	4e	42
6	1f	R = 4-NMe_2_	40	3	4f	58
7	1g	R = 4-Cl	40	3	4g	48

a Reaction conditions: dibenzalacetone (1 mmol), 2,4-dinitrohydrazine (1 mmol), isoniazid (1 mmol), ethanol (10 mL), and 0.02 g catalyst.

b Refluxing.

c All the reactions were monitored using TLC.

d Isolated yield.

**Table 4:** Optimized reaction conditions for the synthesis of 4h–n^a^

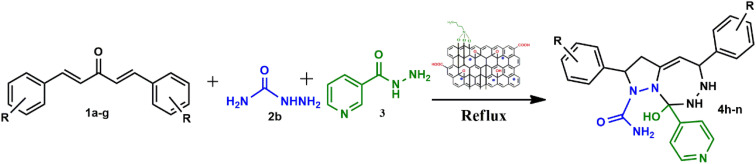						
Entry	Dibenzalacetone	R	Temp^b^ (in °C)	Time^c^ (h)	Product	Yield^d^ (in %)
1	1a	R = H	45	3.5	4h	53
2	1b	R = 4-OH	40	3	4i	65
3	1c	R = 4-NO_2_	50	4	4j	43
4	1d	R = 4-OH and 3-OCH_3_	45	4	4k	55
5	1e	R = 2-OH	55	4.5	4l	42
6	1f	R = 4-NMe_2_	40	3.5	4m	58
7	1g	R = 4-Cl	40	3	4n	48

a Reaction conditions: dibenzalacetone (1 mmol), 2,4-dinitrohydrazine (1 mmol), isoniazid (1 mmol), ethanol (10 mL), and r.t. 25–30 °C.

b Refluxing.

c All the reactions were monitored using TLC.

d Isolated yield.

**Table 5:** Optimized reaction conditions for the synthesis of 4b^a^

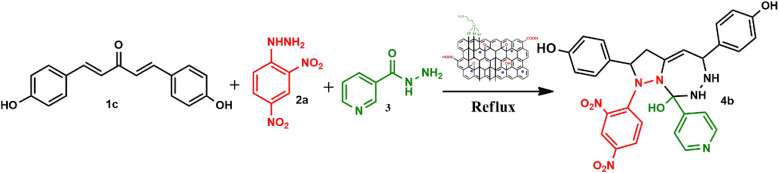					
Entry	Catalyst loading (mg)	Solvent	Temp. (in °C)	Time^c^ (h)	Yield (in %)^b^
1	20 (CuFe_2_O_4_@GO-NH_2_)	CH_3_CN	r.t.	3	30
2	20 (CuFe_2_O_4_@GO-NH_2_)	CH_3_CN	40	2.5	35
3	20 (CuFe_2_O_4_@GO-NH_2_)	CH_3_CN	Reflux	2	45
4	30 (CuFe_2_O_4_@GO-NH_2_)	CH_3_CN	Reflux	3	28
5	20 (CuFe_2_O_4_@GO-NH_2_)	DCM	Reflux	2	55
6	20 (CuFe_2_O_4_@GO-NH_2_)	DCM	40	3	45
7	30 (CuFe_2_O_4_@GO-NH_2_)	DCM	Reflux	1.5	48
8	20 (CuFe_2_O_4_@GO-NH_2_)	DMF	Reflux	2.5	58
9	30 (CuFe_2_O_4_@GO-NH_2_)	DMF	Reflux	2	50
10	20 (CuFe_2_O_4_@GO-NH_2_)	H_2_O	Reflux	3	40
11	20 (CuFe_2_O_4_@GO-NH_2_)	EtOH	r.t.	3	42
12	20 (CuFe_2_O_4_@GO-NH_2_)	EtOH	r.t.	3	43
13	20 (CuFe_2_O_4_@GO-NH_2_)	EtOH	Reflux	3	65
14	30 (CuFe_2_O_4_@GO-NH_2_)	EtOH	Reflux	3	60
15	60 (CuFe_2_O_4_@GO)	EtOH	Reflux	24	No product
16	80 (GO)	EtOH	Reflux	24	No product
17	65 (CuFe_2_O_4_)	EtOH	Reflux	24	No product

a Reaction conditions: dibenzalacetone (1 mmol), 2,4-dinitrohydrazine (1 mmol), isoniazid (1 mmol), solvent (10 mL), and 25–30 °C.

b Isolated yield.

c All the reactions were monitored using TLC.

**Table 6:** Optimized reaction conditions for the synthesis of 4i^a^

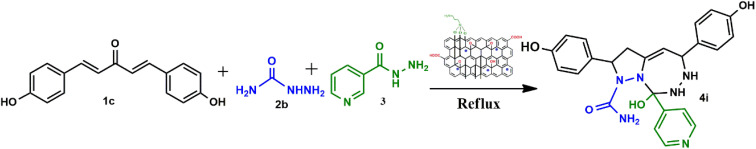					
Entry	Catalyst loading (mg)	Solvent	Temp. (in °C)	Time^c^ (h)	Yield^b^ (in %)
1	20 (CuFe_2_O_4_@GO-NH_2_)	CH_3_CN	r.t.	3	25
2	20 (CuFe_2_O_4_@GO-NH_2_)	CH_3_CN	40	2.5	30
3	20 (CuFe_2_O_4_@GO-NH_2_)	CH_3_CN	Reflux	2	34
4	30 (CuFe_2_O_4_@GO-NH_2_)	CH_3_CN	Reflux	3	32
5	20 (CuFe_2_O_4_@GO-NH_2_)	DCM	Reflux	2	50
6	20 (CuFe_2_O_4_@GO-NH_2_)	DCM	40	3	43
7	30 (CuFe_2_O_4_@GO-NH_2_)	DCM	Reflux	1.5	45
8	20 (CuFe_2_O_4_@GO-NH_2_)	DMF	Reflux	2.5	43
9	30 (CuFe_2_O_4_@GO-NH_2_)	DMF	Reflux	2	46
10	20 (CuFe_2_O_4_@GO-NH_2_)	H_2_O	Reflux	3	35
11	20 (CuFe_2_O_4_@GO-NH_2_)	EtOH	r.t.	3	43
12	20 (CuFe_2_O_4_@GO-NH_2_)	EtOH	40	3	50
13	20 (CuFe_2_O_4_@GO-NH_2_)	EtOH	Reflux	3	63
14	30 (CuFe_2_O_4_@GO-NH_2_)	EtOH	Reflux	3	61
15	60 (CuFe_2_O_4_@GO)	EtOH	Reflux	24	No product
16	80 (GO)	EtOH	Reflux	24	No product
17	65 (CuFe_2_O_4_)	EtOH	Reflux	24	No product

a Reaction conditions: dibenzalacetone (1 mmol), semicarbazide (1 mmol), isoniazid (1 mmol), solvent (10 mL), and 25–30 °C.

b Isolated yield.

c All the reactions were monitored using TLC.

#### 2.1.3. Mechanistic approach

Under catalytic conditions, benzaldehyde derivatives react with acetone through Claisen–Schmidt condensation pathway in which the carbonyl of benzaldehyde derivatives activated with *via* Cu^2+^ and Fe^3+^ of CuFe_2_O_4_ acts as Lewis acid and hydrogen bonding with carboxylic, hydroxy functional groups on catalyst, thus acts as electrophile and active methyl hydrogen of acetone abstract by the basic functionality *i.e.* NH_2_ of CuFe_2_O_4_@GO-NH_2_ and with removal of water molecules dibenzalacetone (1a–g) were synthesized. Furthermore, the reaction is ushered in with CuFe_2_O_4_@GO-NH_2_ activating the carbonyl group of dibenzalacetone (1a–g) with the Brønsted acidic functionality, making the carbonyl carbon more electrophilic, which then undergoes 1,2-nucleophilic addition with enhanced nucleophilicity of the terminal –NH_2_ of hydrazine (2a or 2b) by the basic sites of the catalyst, generating an alkoxide intermediate stabilized at the catalyst surface, followed by the formation of corresponding hydrazone (II) with the removal of water. The presence of dinitro on benzene reduces the lone pair-donating tendency of –NH_2_ of hydrazine, thus making it unsuitable for Michael addition.^41^ Therefore, the basic functionality of the catalyst plays an important role in enhancing nucleophilicity. Moreover, in the presence of the bifunctionality of the catalyst and an increase in temperature forces the hydrazone (II) to undergo cyclization to give intermediate (III), in which proton rearrangement gives ^4,5^ dihydropyrazole intermediate (IV). Now, with the subsequent addition of activated carbonyl of isoniazid (3) *via* acidic sites of catalyst onto stabilized intermediate (IV) on catalyst surface aza-Michael type cyclization takes place to lead to the fully formed adduct, in which the hydrazine (2a or 2b) fragment and aminopyridine (3) remain attached through N–N and C–N linkages, yielding the final products 4a–g with the regeneration of the catalyst, as represented in Scheme 3. Hence, to incorporate the desired intermediates and products, dual acidic and basic sites are required in the catalyst, revealing the importance of the synthesized catalyst, which cannot be achieved with the existing catalysts, GO or CuFe_2_O_4_@GO, which lack basic functionality.

### 2.2. *In vitro* assay-based α-amylase and α-glucosidase inhibition

**Fig. 3:**
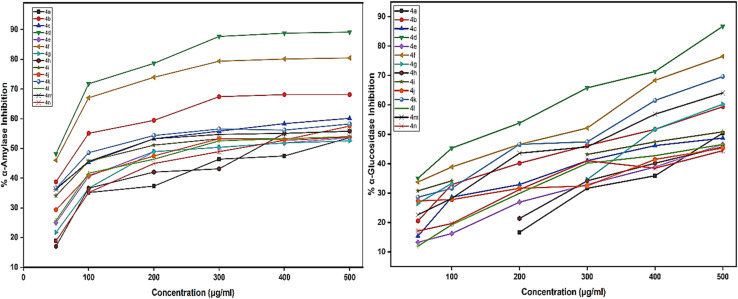
Graphical representations of the inhibition activity of the compounds 4a-4n against the α-amylase and α-glucosidase enzymes.

The substituted fused pyrazole^1,2,4^-triazepine heterocycles (4a–4n) were evaluated for *in vitro* assay-based antidiabetic activity against α-amylase (EC no. 3.2.1.1) and α-glucosidase (EC no. 3.2.1.20), which are responsible for type-II diabetes. The appreciable percentage of the inhibitory activity of compounds (4a-4n) obtained by triplicate experiments (Tables S1 and S2) against α-amylase and α-glucosidase is illustrated in Table 7 and Table 8, respectively. It was found that compound 4d, among all the synthesized compounds, shows a greater extent of inhibition against both enzymes, with an IC_50_ value comparable with the reference drug acarbose reported in literature.^42,43^ For all the compounds, with the increase in concentration, inhibitory activity also increases (Fig. 3). The results reveal that compounds with electron withdrawing substituents show a high value of IC_50_ and less inhibition compared with electron donating substituents and that hydroxy and amine substituents favor more inhibition up to ∼90% at 500 µg mL, while acarbose inhibits only 72%.

**Table 7:** Percentage α-amylase inhibition activity of the compounds (4a–4n) at different concentrations with ±S.D. (standard deviation)

Compounds	% Inhibition (µg mL^−1^)						IC_50_ (µg mL^−1^)
	50 µg mL^−1^	100 µg mL^−1^	200 µg mL^−1^	300 µg mL^−1^	400 µg mL^−1^	500 µg mL^−1^	
4a	18.84 ± 2.26	35.14 ± 1.66	37.32 ± 0.63	46.38 ± 0.63	47.46 ± 0.63	53.62 ± 1.25	247.99
4b	39.13 ± 3.81	55.07 ± 0.63	59.42 ± 0.63	67.39 ± 1.09	68.12 ± 0.63	68.11 ± 0.62	226.3
4c	36.59 ± 3.81	45.29 ± 0.63	53.26 ± 1.09	55.80 ± 0.63	58.33 ± 0.63	60.14 ± 1.25	165.38
4d	48.18 ± 2.73	71.74 ± 1.88	78.62 ± 0.63	87.68 ± 1.66	88.77 ± 0.63	89.13 ± 0.00	123.79
4e	25.00 ± 1.88	40.58 ± 1.66	48.91 ± 1.09	50.36 ± 0.63	51.81 ± 0.63	53.62 ± 1.25	198.68
4f	46.01 ± 1.66	67.03 ± 0.63	73.91 ± 1.09	79.35 ± 1.09	80.07 ± 0.63	80.43 ± 0.00	137.48
4g	21.73 ± 2.17	36.59 ± 1.26	52.54 ± 0.63	48.91 ± 1.09	54.71 ± 5.02	51.81 ± 0.62	237.98
4h	17.02 ± 2.26	42.03 ± 4.39	36.59 ± 0.63	43.12 ± 0.63	55.07 ± 0.63	55.79 ± 0.62	175.39
4i	34.05 ± 3.32	45.65 ± 2.17	51.09 ± 1.09	53.26 ± 1.09	53.26 ± 1.09	53.98 ± 1.25	184.61
4j	29.34 ± 2.87	40.94 ± 1.26	47.46 ± 0.63	53.26 ± 1.09	52.90 ± 0.63	57.49 ± 1.10	237.92
4k	36.59 ± 2.26	48.55 ± 2.51	54.35 ± 1.09	56.52 ± 1.09	56.16 ± 0.63	58.21 ± 0.82	166.97
4l	25.72 ± 2.51	41.67 ± 0.63	46.38 ± 0.63	52.54 ± 0.63	53.26 ± 1.09	53.98 ± 0.62	272.14
4m	36.23 ± 3.32	45.65 ± 1.09	53.26 ± 2.26	54.71 ± 0.63	55.07 ± 0.63	55.79 ± 0.62	173.62
4n	18.84 ± 3.81	35.14 ± 1.66	44.93 ± 0.63	48.91 ± 1.09	52.72 ± 0.77	53.80 ± 0.76	259.53
Concentration	250 µg mL^−1^	500 µg mL^−1^	750 µg mL^−1^	1000 µg mL^−1^	—	—	IC_50_ (µg mL^−1^)
Acarbose^42^	61.5	72.08	82.23	88.19 ± 0.01	—	—	171.8

**Table 8:** Percentage α-glucosidase inhibition activity of the compounds (4a–4n) at different concentrations with ±S.D. (standard deviation)

Compounds	% Inhibition (µg mL^−1^)						IC_50_ (µg mL^−1^)
	50 µg mL^−1^	100 µg mL^−1^	200 µg mL^−1^	300 µg mL^−1^	400 µg mL^−1^	500 µg mL^−1^	
4a	20.51 ± 1.28	—	16.67 ± 1.81	31.62 ± 0.74	35.90 ± 7.80	50.43 ± 28.56	273.42
4b	15.38 ± 1.28	32.91 ± 1.96	40.17 ± 23.04	46.15 ± 1.28	51.71 ± 1.48	59.40 ± 33.80	261.8
4c	35.04 ± 0.74	28.63 ± 1.96	32.91 ± 19.65	41.03 ± 1.28	46.15 ± 1.28	48.72 ± 27.87	272.79
4d	13.25 ± 0.74	45.30 ± 1.96	53.85 ± 31.57	65.81 ± 1.96	71.37 ± 1.96	86.75 ± 49.81	167.49
4e	33.76 ± 0.74	16.24 ± 2.67	26.92 ± 15.37	32.91 ± 1.96	38.89 ± 1.48	46.58 ± 26.35	279.17
4f	26.50 ± 0.74	38.89 ± 1.48	46.58 ± 26.79	52.14 ± 0.74	68.38 ± 3.92	76.50 ± 43.83	199.43
4g	—	33.33 ± 1.28	—	34.62 ± 1.28	51.71 ± 1.48	60.26 ± 34.50	254.56
4h	30.77 ± 1.28	—	21.37 ± 0.91	34.19 ± 0.74	40.17 ± 1.48	45.73 ± 26.35	253.1
4i	27.35 ± 0.74	34.19 ± 1.96	—	43.16 ± 1.96	47.44 ± 2.56	50.85 ± 28.99	199.19
4j	28.63 ± 0.74	27.78 ± 1.96	17.09 ± 9.13	32.48 ± 1.48	41.45 ± 3.23	45.30 ± 25.61	252.46
4k	11.97 ± 0.74	31.62 ± 1.48	46.58 ± 26.35	47.44 ± 1.28	61.54 ± 2.56	69.66 ± 39.74	237.67
4l	22.65 ± 0.74	19.23 ± 1.28	29.91 ± 17.10	40.17 ± 1.48	42.74 ± 1.48	46.58 ± 27.11	261.26
4m	17.09 ± 0.74	28.21 ± 1.28	43.59 ± 24.53	45.73 ± 0.74	56.84 ± 1.96	64.10 ± 36.77	273.29
4n	—	19.66 ± 1.96	31.20 ± 17.77	41.03 ± 2.56	38.46 ± 1.28	44.44 ± 25.28	254.47

### 2.3. *In silico* studies

#### 2.3.1. Molecular docking

**Fig. 4:**
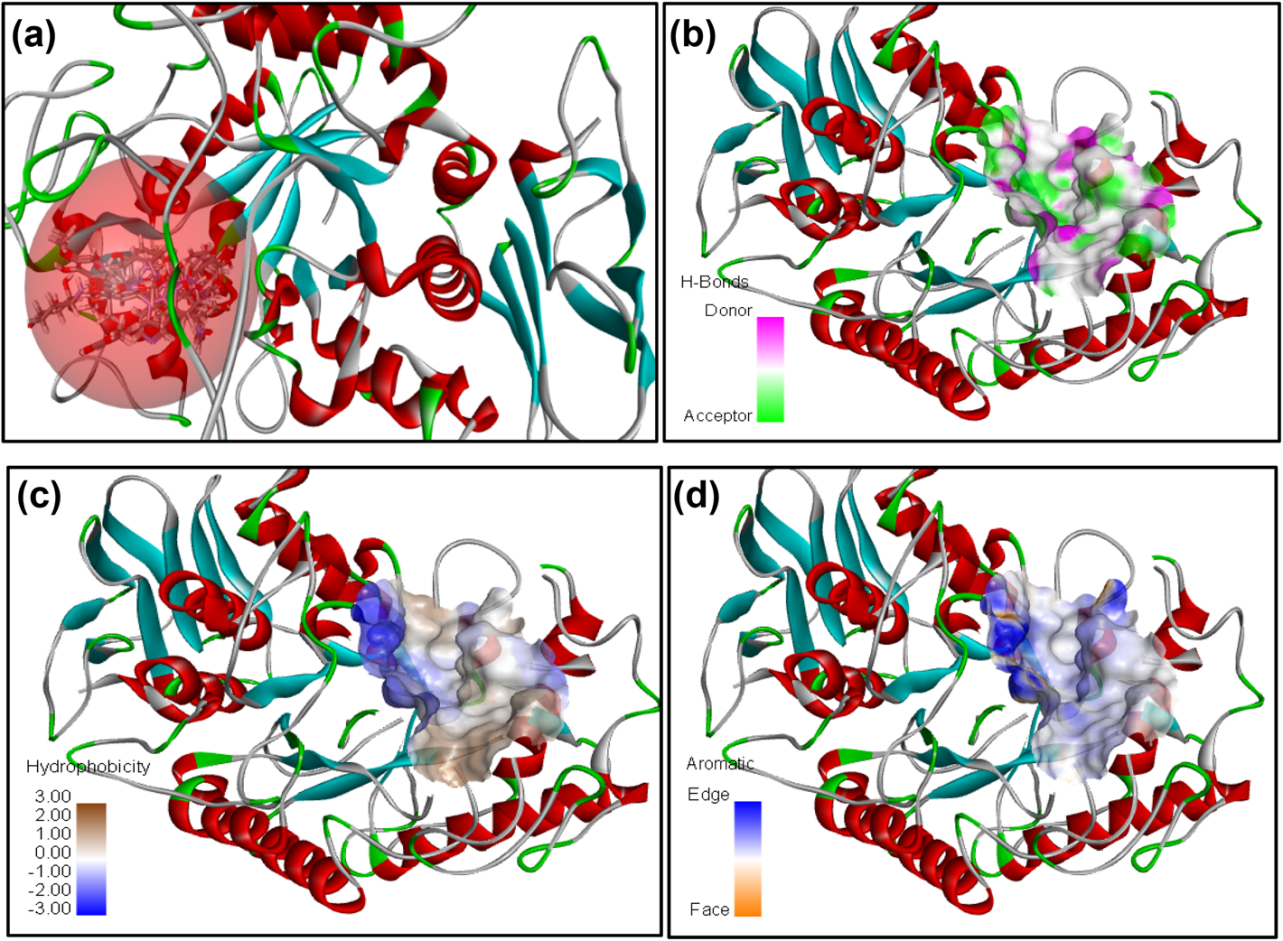
2QV4 receptor grid model (10.53 Å; 48.13, 26.09, and 12.38) with multiple docked ligands (a), associated susceptible regions for hydrogen bonding (b), hydrophobicity (c), and aromaticity (d).

**Fig. 5:**
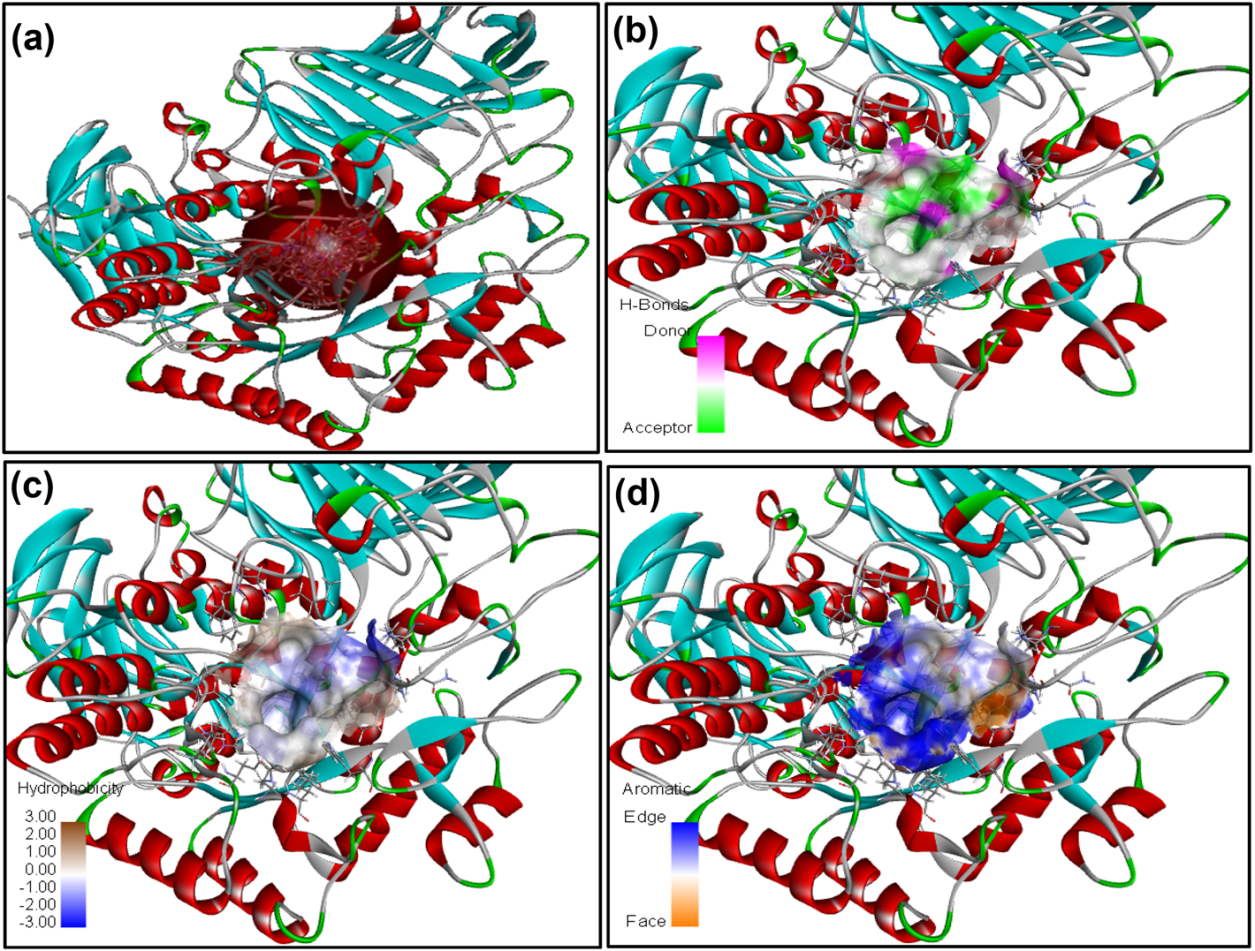
3W37 receptor grid model (9.89 Å; 0.108, −1.91, and −23.05) with multiple docked ligands (a); associated susceptible regions for hydrogen bonding (b), hydrophobicity (c), and aromaticity (d).

**Fig. 6:**
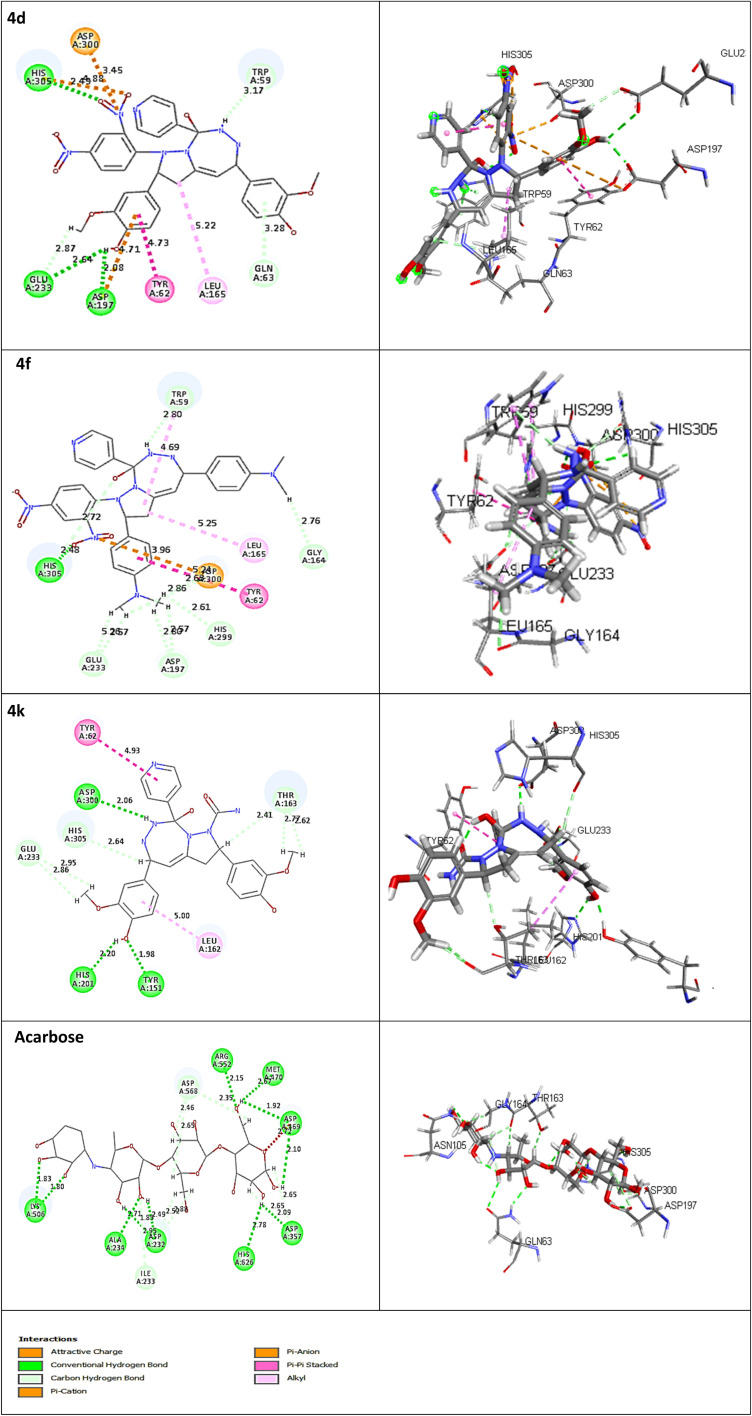
Closest interactions between the active site residues of the α-amylase (2QV4) with the synthesized compounds 4d, 4f, 4k and the reference drug acarbose, respectively, in two-dimensional (2D; left) and three-dimensional (3D; right) representations.

**Fig. 7:**
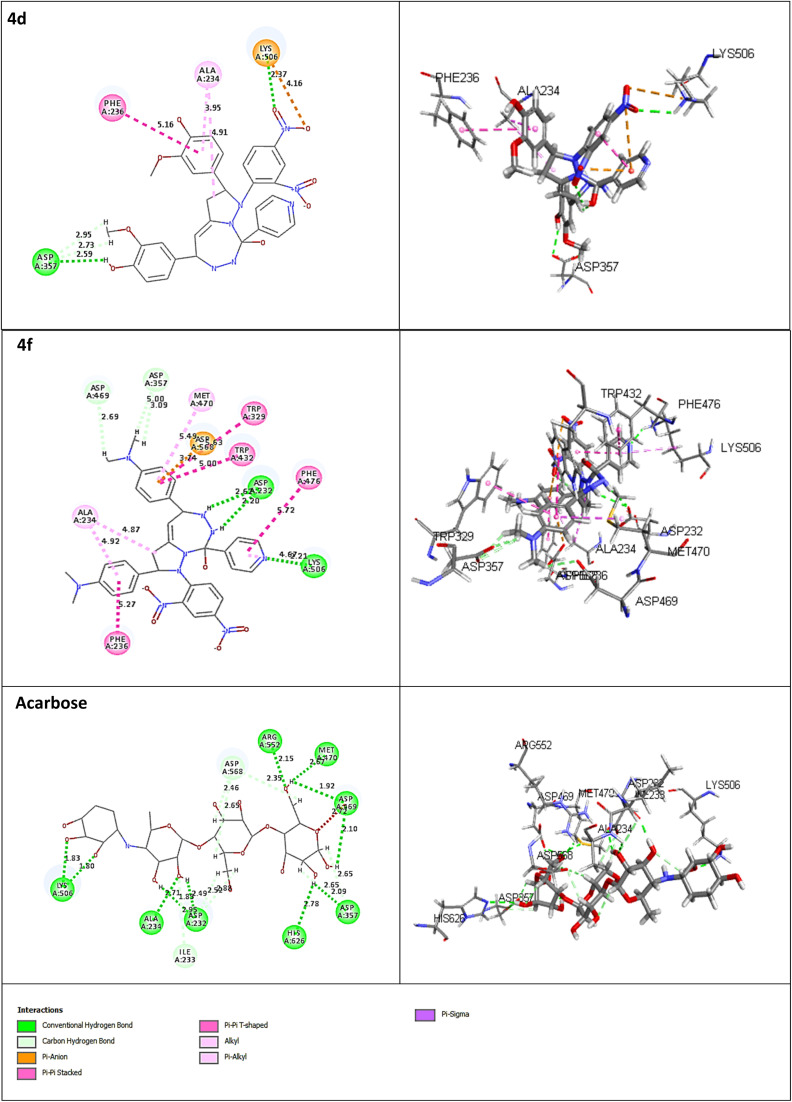
Closest interactions between the active site residues of the α-glucosidase (3W37) with the synthesized compounds 4d, 4f, and the reference drug acarbose, respectively, in two-dimensional (2D; left) and three-dimensional (3D; right) representations.

The receptor protein models with PDB ID: 2QV4 and 3W37, associated with α-amylase and α-glucosidase enzymes, respectively, were validated and used for molecular docking evaluation. To optimize and identify the docking conditions, receptor grid sphere and active binding sites, the reference co-crystal ligand (acarbose) was successfully re-docked with both proteins with acceptable RMSDs of 1.031 Å and 1.026 Å. The same receptor sites were used to disclose the binding interactions of 16 compounds (4a–4p) with 2QV4 (Fig. S48) and 3W37 (Fig. S49) enzyme proteins. The receptor grid sphere models with multiple docked ligands and hydrophobic, aromaticity, and hydrogen bonding susceptible regions of 2QV4 and 3W37 are shown in Fig. 4 and 5, respectively. All the 16 compounds exhibited satisfactory binding affinity with cdocker energy and cdocker interaction energy, as summarized in Tables 9 and 10, respectively. α-Amylase hydrolyses internal α-1,4-linkages of starch (endo action), while glucosidase trims terminal α-glucosidase bond (exo action), as their catalytic residues such as GLU233, ASP300, and ASP197 of 2QV4; ASP469 and ASP568 of 3W37 and substrate recognition residues GLN63, HIS201, TYR62, HIS299, ARG195, TYR151, HIS101, ALA106, THR163, GLY164 of 2QV4; PHE236, ASN237, and SER497 of 3W37 reflect these roles.^44–46^ The interaction with catalytic residues GLU233 and ASP300 of 2QV4 and pocket geometry residues fostering the inhibition activity of 4d and 4k, respectively, against 2QV4, similarly with ASP469, ASP568 of 3W37 in the case of 4d, 4f, and 4m, respectively, against 3W37 provides results comparable with those of acarbose. The stronger inhibition against 2QV4 of acarbose than 4d, and that of 4d and 4f more than 4k is due to the presence of a greater number of hydrogen bonds with the shortest distances of interaction associated with a high-cdocker interaction energy (associated with only protein-ligand binding energy), as depicted in Fig. 6, which is also responsible for a stable protein-ligand complex, as reflected in the high-cdocker energy (associated with interaction energy and ligand strain). Similarly, the closest binding interactions of 4d and 4f against α-glucosidase protein 3W37 are illustrated in Fig. 7. Thus, the *in silico* docking results were in accordance with the *in vitro* assay based experiments.

**Table 9:** Cdocker energy and cdocker interaction energy of the compounds (4a-4n) with the α-amylase protein receptor (2QV4)

Entry	Compounds	-Cdocker energy (kcal mol^−1^)	-Cdocker interaction energy (kcal mol^−1^)
1	4a	−6.04544	43.2612
2	4b	−7.39189	52.7811
3	4c	8.0601	44.6678
4	4d	5.802631	78.3542
5	4e	−3.51015	51.3859
6	4f	−1.87997	67.8439
7	4g	−5.2506	47.4387
8	4h	4.75304	42.9307
9	4i	6.5959	49.1111
10	4j	2.4342	43.698
11	4k	10.9233	60.3177
12	4l	2.73058	43.9564
13	4m	7.70246	45.0517
14	4n	5.21054	39.9079
15	Acarbose	−19.8945	58.0488

**Table 10:** Cdocker energy and cdocker interaction energy of the compounds (4a–4n) with the α-glucosidase protein receptor (3W37)

Entry	Compounds	-Cdocker energy (kcal mol^−1^)	-Cdocker interaction energy (kcal mol^−1^)
1	4a	−7.89474	40.9305
2	4b	−3.98288	45.5413
3	4c	−13.7246	46.7802
4	4d	2.9835	78.5975
5	4e	−2.57054	43.0875
6	4f	2.12069	69.2093
7	4g	8.9162	44.7756
8	4h	4.75304	42.9307
9	4i	6.5959	49.1111
10	4j	2.4342	43.698
11	4k	10.9233	58.3177
12	4l	2.73058	43.9564
13	4m	8.04299	47.3917
14	4n	5.21054	39.9079
15	Acarbose	−5.85001	71.2107

#### 2.3.2. ADMET analysis

**Fig. 8:**
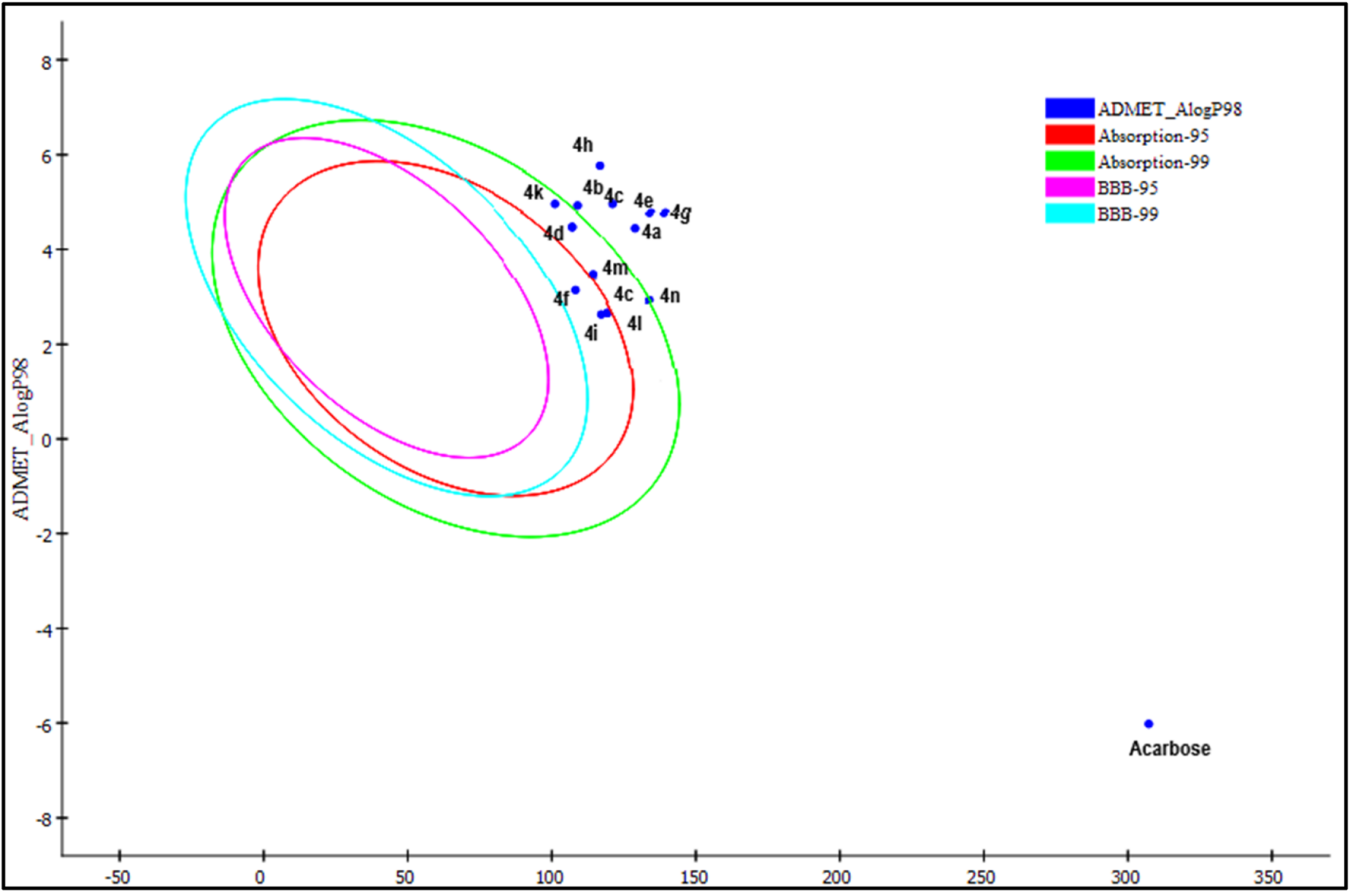
Plot of PSA_2D (two-dimensional polar surface area) *versus* A Log P98 for the compounds 4a-4p and the standard drug acarbose, representing 95% and 99% assurance limits associated with the BBB (blood–brain barrier) and intestinal absorption model.

The plot between A log *P* and PSA reveals much better drug likeness than acarbose (Fig. 8). A log *P* and PSA are the standard descriptors for exploring drug characteristics, like oral absorption, solubility and blood–brain barrier. Acarbose shows poor absorption, while all the compounds (4a–4p) fall within the absorption ellipses and outside the BBB ellipses, indicating favorable oral absorption, while no penetration to the BBB is also advantageous for an antidiabetic drug because for an antidiabetic drug, CNS penetration is not required and helps to eradicate neurological adverse effects. Good solubility supports absorption properties. Some of the compounds show hepatotoxicity (risk of liver toxicity) and CYP2D6 inhibition (risk of drug–drug interactions), but compounds 4d, 4f, 4k, and 4m with good docking and *in vitro* activity exhibit excellent drug likeness over acarbose with no signs of toxicity, as shown in Table 11. The true/false PPB (plasma protein binding) level for compounds divulges the binding/non-binding of drugs with protein binding, *i.e.* if false, then enhancing efficacy due to the presence of the drug in circulation.

**Table 11:** ADMET predictions of the compounds (4a–4**p**) and acarbose

Entry	Compounds	BBB level^a^	Absorption level^b^	Solubility level^c^	Hepatotoxicity	CYP2D6	PPB level	A log P98	PSA_2D
1	4a	4	3	3	True	False	False	4.241	108.242
2	4b	4	1	3	False	False	False	4.466	114.947
3	4c	4	2	3	False	False	False	5.657	119.873
4	4d	4	1	4	False	False	False	4.624	117.733
5	4e	4	2	3	False	False	False	4.63	130.308
6	4f	4	1	4	False	False	False	5.233	135.694
7	4g	4	2	0	True	False	True	4.93	133.888
8	4h	4	0	2	True	False	False	6.223	140.048
9	4i	4	1	3	False	False	False	3.96	121.679
10	4j	4	2	3	False	False	False	4.444	130.048
11	4k	4	1	3	False	False	False	4.773	135.048
12	4l	4	2	1	False	False	True	3.432	101.679
13	4m	4	1	3	False	False	False	5.768	116.753
14	4n	4	1	0	True	False	False	3.727	109.539
15	Acarbose	4	3	1	False	False	False	−6.02	307.246

a 0, 1, 2, 3, and 4 denote very high, high, medium, low, and undefined, respectively.

b 0, 1, 2, and 3 denote good absorption, moderate absorption, low absorption, and very low absorption, respectively.

c 0, 1, 2, 3, 4, and 5 denote extremely low, very low but possible, low, good, optimal, and too soluble, respectively.

#### 2.3.3. Lipinski's rule of five

The molecular properties of 4a–4p are shown in Table 12 and demonstrate that all the compounds follow Lipinski's Rule as per reported literature,^47^ while acarbose violates the rule with respect to low negative A log *P* values and a high number of hydrogen bond acceptors and donors. Thus, these compounds are superior to acarbose.

**Table 12:** Physiochemical molecular properties of the compounds (4a–4p) and acarbose

Entry	Compound	A log P	Molecular weight	Num_H_donor	Num_H_acceptor	Num_rotable_bonds	Num_rings	Num_aromatic_rings	Molecular_FractionalPolarsurface_area
1	4a	4.405	551.553	3	10	6	6	4	0.301
2	4b	3.921	583.552	5	12	6	6	4	0.363
3	4c	4.194	641.548	3	14	8	6	4	0.417
4	4d	3.888	643.603	5	14	8	6	4	0.351
5	4e	3.921	583.552	5	12	6	6	4	0.363
6	4f	4.729	637.688	3	12	8	6	4	0.252
7	4g	5.733	620.443	3	10	6	6	4	0.278
8	4h	1.976	428.486	4	6	3	5	3	0.26
9	4i	1.459	520.537	6	10	5	5	3	0.329
10	4j	1.492	460.485	6	8	3	5	3	0.34
11	4k	2.301	514.622	4	8	5	5	3	0.211
12	4l	3.305	497.376	4	6	3	5	3	0.236
13	4m	1.492	460.485	6	8	3	5	3	0.34
14	4n	1.765	518.481	4	10	5	5	3	0.408
15	Acarbose	−6.02	617.595	13	18	8	4	0	0.526

#### 2.3.4. Toxicity predictions

In designing a drug for therapeutic cure, toxicity prediction by computer-assisted technology (TOPKAT) is the best tool that uses the QSTR model for toxicity evaluation. The prediction is performed by scrutinizing the components within the optimal predictive space (OPS), and the results are considered false positives if they lie outside the range. The results of toxicity predictions against NTP, Carcinogenic Potency TD50, WOE, Skin Irritancy, Ames, Oral LD_50_, Inhalational LC50, and Daphnia EC50 are summarized in Table 13.

**Table 13:** TOPKAT toxicity evaluation of the compounds (4a–4n) and acarbose

Entry	Compound	TOPKAT rat female NTP predictions	TOPKAT rat female NTP score	TOPKAT carcinogenic potency TD50 mouse score	TOPKAT carcinogenic potency TD50 mouse applicability	TOPKAT_Ames prediction	TOP-KAT ames score	TOPKAT rat oral LD_50_	TOPKAT rat oral LD_50_ applicability	TOPKAT rat inhalational LC50	TOPKAT rat inhalational LC50 applicability	TOPKAT skin irritancy	TOPKAT daphnia EC50	TOPKAT daphnia EC50 applicability	TOPKAT WOE	TOPKAT WOE score
1	4a	Non-carcinogen	−0.72634	2.43405	OPS in range	Non-mutagen	−3.80992	1.57155	1.57155	184.898	Out of range	Irritant	0.0831	OPS in range	Non-carcinogen	−5.92504
2	4b	Non-carcinogen	−0.21921	3.76946	OPS in range	Non-mutagen	−2.9017	0.98062	0.98062	167.41	Out of range	Irritant	0.16515	Out of range	Non-carcinogen	−2.94901
3	4c	Carcinogen	1.10536	1.62186	OPS in range	Non-mutagen	−1.09034	0.75881	0.75881	335.971	Out of range	Irritant	0.13944	Out of range	Non-carcinogen	−0.02215
4	4d	Non-carcinogen	−2.99282	0.90761	Out of range	Non-mutagen	−5.95126	0.67771	0.67771	285.705	OPS in range	Non-irritant	0.06869	OPS in range	Non-carcinogen	−2.80805
5	4e	Non-carcinogen	−1.86286	2.20215	OPS in range	Non-mutagen	−5.34796	0.60059	0.60059	89.0316	Out of range	Irritant	0.85948	Out of range	Non-carcinogen	−2.14595
6	4f	Non-carcinogen	−3.94519	0.27827	Out of range	Non-mutagen	−11.7199	0.43574	0.43574	227.559	Out of range	Non-irritant	0.0919	OPS in range	Non-carcinogen	−2.98811
7	4g	Non-carcinogen	−5.63878	3.80829	OPS in range	Non-mutagen	−10.7404	0.45164	0.45164	627.074	Out of range	Irritant	2.11727	Out of range	Non-carcinogen	−3.55772
8	4h	Non-carcinogen	−3.71762	4.13973	OPS in range	Non-mutagen	−11.1007	1.26447	1.26447	393.007	OPS in range	Irritant	1.05806	Out of range	Non-carcinogen	−7.26285
9	4i	Non-carcinogen	−3.55682	3.79609	OPS in range	Non-mutagen	−9.48178	0.86836	0.86836	215.995	OPS in range	Non-irritant	2.31761	Out of range	Non-carcinogen	−4.03981
10	4j	Non-carcinogen	−2.85976	0.28653	Out of range	Non-mutagen	−7.00246	0.41628	0.41628	443.353	Out of range	Irritant	2.41773	Out of range	Non-carcinogen	−0.23001
11	4k	Non-carcinogen	−7.82134	0.92834	Out of range	Non-mutagen	−12.3051	0.29298	0.29298	374.515	Out of range	Non-irritant	0.06976	OPS in range	Non-carcinogen	−3.95053
12	4l	Non-carcinogen	−8.05615	1.80478	OPS in range	Non-mutagen	−18.7953	15.4381	15.4381	2.7057	OPS in range	Irritant	3.22558	Out of range	Non-carcinogen	−6.85731
13	4m	Non-carcinogen	−18.2777	44.9352	OPS in range	Mutagen	7.69559	1.19144	1.19144	806.217	Out of range	Non-irritant	5.7722	Out of range	Non-carcinogen	−3.15278
14	4n	Non-carcinogen	−0.30352	1.76449	OPS in range	Non-mutagen	−6.78923	1.40475	1.40475	289.944	OPS in range	Irritant	0.05515	OPS in range	Non-carcinogen	−2.46574
15	Acarbose	Non-carcinogen	−5.13205	0.27233	Out of range	Non-mutagen	−12.6911	0.83738	0.83738	380.072	Out of range	Non-irritant	0.88399	Out of range	Non-carcinogen	−3.60821

### 2.4. Density functional theory insights

**Fig. 9:**
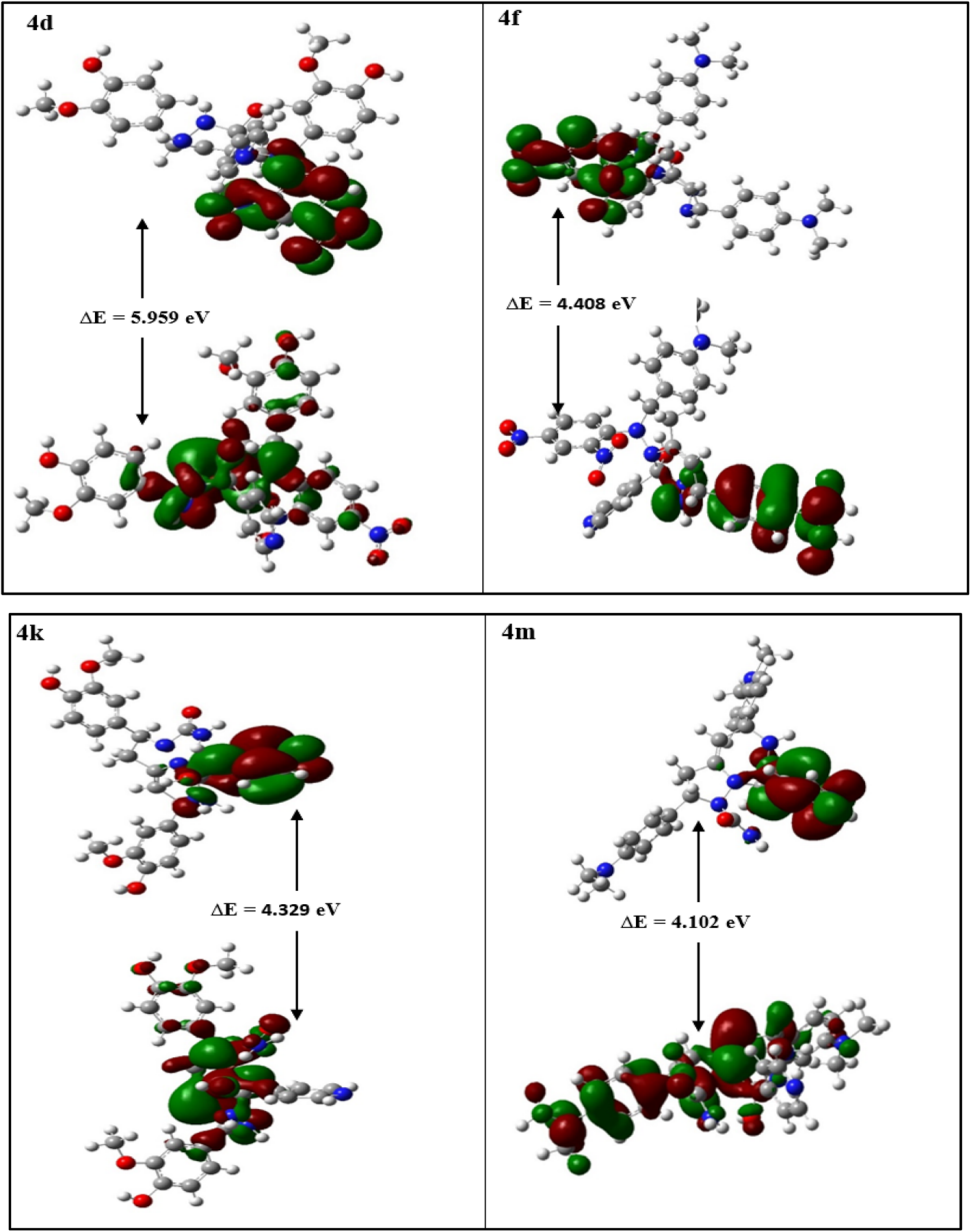
Molecular orbital (HOMO and LUMO) representations of the compounds 4d, 4f, 4k and 4m, respectively, with their corresponding HOMO–LUMO energy gaps.

**Fig. 10:**
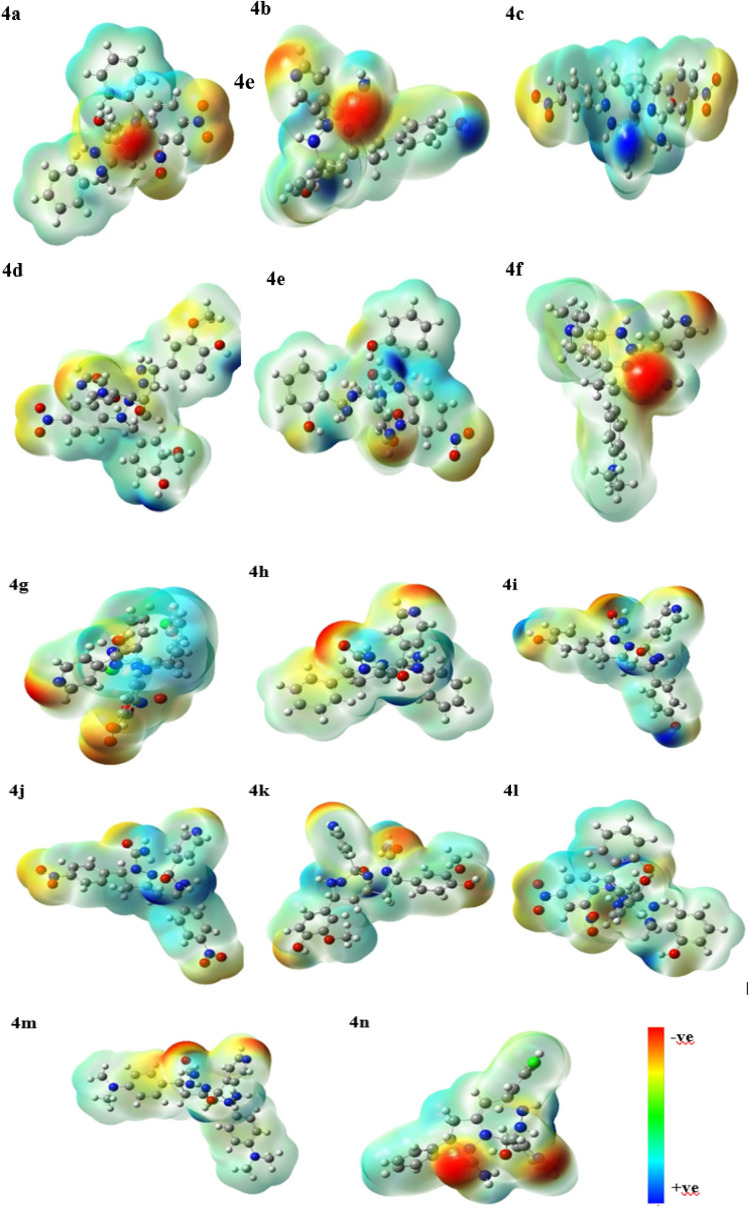
Molecular electrostatic potential (MEP) surface contour maps of the compounds (4a–4n).

In determining the reactivity and stability of the molecules, frontier molecular orbitals are found to be a consequential approach. All the molecules were optimized to produce stable structures without any imaginary frequency. The HOMO and LUMO were drawn for all the molecules, and their energy gap was estimated (Fig. S50). The high energy gap between HOMO–LUMO depicts the inertness of the compound, which is associated with the hardness of compounds.^48^ Thus, a lower value of chemical hardness (*η*) indicates high reactivity. Electrophilicity index (*ω*) gives an idea about the lowering in energy when an electron flows from HOMO to LUMO, thus revealing the energy changes when a molecule is saturated by the addition of an electron, which indicates the reactivity.^49^ The negative of electronegativity is termed the electronic chemical potential (*µ*). The values of the energy gap, *η*, *ω*, and *µ* are summarized in Table 14. Among all the compounds, 4d, 4f, 4k, and 4m were found to be the most reactive with the least value of *η* (Fig. 9) thus in agreement with biological activity. The MEP surface contour is depicted in Fig. 10 with red and blue regions, indicating electronegative and electropositive densities, respectively. A greater extent of red region in the surface contour of 4d and 4k exposes the compound susceptibility towards electrophile, showing efficient hydrogen bonding, which again demonstrates good agreement with biological activity.

**Table 14:** Density functional theory (DFT)-based electronic properties of the compounds (4a–4n)

Entry	Compounds	*E*_HOMO_ (eV)	*E*_LUMO_ (eV)	Δ*E* = E_LUMO_ - E_HOMO_	Chemical hardness *η* = 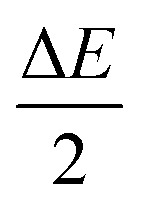	Chemical potential *µ* = 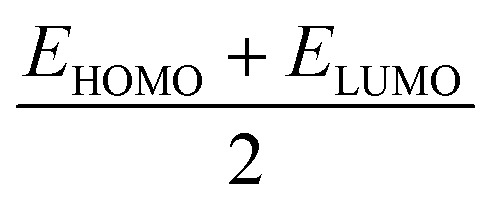	Electrophilicity = 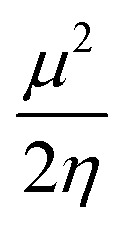
1.	4a	−5.96039	−3.51571	2.444672	1.222336	−4.73805	9.182871
2.	4b	−5.81372	−3.50483	2.308887	1.154444	−4.65927	9.402285
3.	4c	−6.0608	−3.1021	2.958696	1.479348	−4.58145	7.094228
4.	4d	−7.78164	−1.82208	5.959569	2.979784	−4.80186	3.869048
5.	4e	−6.20991	−3.63054	2.579369	1.289684	−4.92023	9.385497
6.	4f	−6.73918	−2.33093	4.408247	2.204123	−4.53505	4.665505
7.	4g	−7.30817	−5.51058	1.797585	0.898793	−6.40937	22.85292
8.	4h	−5.24037	−3.37966	1.860716	0.930358	−4.31001	9.983373
9.	4i	−5.65888	−3.05938	2.599505	1.299753	−4.35913	7.309859
10.	4j	−5.88746	−3.47598	2.411474	1.205737	−4.68172	9.089259
11.	4k	−5.9397	−1.60983	4.329878	2.164939	−3.77477	3.290821
12.	4l	−5.98678	−3.49449	2.492292	1.246146	−4.74063	9.017246
13.	4m	−4.99302	−0.89036	4.102663	2.051331	−2.94169	2.109247
14.	4n	−5.62813	−3.97014	1.657991	0.828995	−4.79914	13.89135

## 3. Conclusions

In summary, pyrazole fused[1,2,4]-triazepine embracing compounds (4a–4n) were synthesized in the presence of as-prepared magnetic nanocatalyst CuFe_2_O_4_@GO-NH_2_, and their structural and morphological properties were corroborated through various spectroscopic measurements. All the compounds exhibit appreciable inhibition activity against dual α-amylase and α-glucosidase enzymes, which is in good agreement with *in silico* molecular docking results. The binding interactions between the receptor proteins and ligand compounds and the stability of the protein-ligand complex due to more efficient hydrogen bonding were clearly elucidated by means of molecular docking. Compound 4d showed satisfactory antidiabetic activity, with an IC_50_ value comparable with the reference drug acarbose. Pharmacokinetic properties determined based on ADMET and Lipinski's Rule of Five for synthesized compounds were more drug-like than those of acarbose. The toxicity of the synthesized compounds was also within the range of the limit with no penetration of compounds to the blood–brain barrier, like acarbose, making them a suitable drug. Additionally, the chemical reactivity, stability and electrophilicity of the synthesized compounds were governed by density functional theory calculations, which are in accordance with biological activity. Thus, the present synthesis, biological activity evaluation and DFT insights with the exploration of pharmacokinetic properties pave the way for synthesized compound 4d as a potent drug candidate.

## 4. Experimental

### 4.1. Chemicals and apparatus

All the chemicals used were of AR grade purity, purchased from Sigma Aldrich and Merck India and used without further purification. The melting point measurements with errors, were carried out in open capillary tubes using a Veego melting point apparatus. The Cole Parmer ultrasonic processor Model CPX 130, with a maximum power of 150 W, operating at an amplitude of 60% and frequency of 20 kHz was used as the ultrasonic radiation source. The spectroscopic ^1^H and ^13^C NMR spectral characterization were performed at 400 and 200 MHz, respectively, in DMSO using a Bruker Advance II 400 NMR spectrometer. Reaction monitoring was performed by means of thin-layer chromatography (TLC) using silica gel 60 F254 precoated aluminum sheets. A UV 1800 benchtop double beam UV-visible spectrophotometer was used to measure absorbances for the evaluation of inhibition activity against enzymes. A Bruker D8 Advance X-ray diffractometer (Cu Kα radiation; *λ* = 0.154 nm) was used to determine the PXRD patterns. FE-SEM images and the corresponding EDAX data were recorded using a JEOL microscope (JSM7100F). Magnetism experiments were carried out using a vibrating sample magnetometer (VSM, JDM-13) at room temperature.

### 4.2. Catalyst synthesis

First, the GO was prepared according to the reported protocol.^50^ Thereafter, 1 g GO was dispersed in 150 mL distilled water under ultrasonic irradiations for 20 min, followed by the addition of FeCl_3_·6H_2_O (169.1 mg) and CuCl_2_·2H_2_O (53.5 mg) to the reaction mixture with the dropwise addition of 30% ammonium hydroxide solution (25 mL) under a nitrogen atmosphere for 3 hours. After completion of the reaction, CuFe_2_O_4_@GO was isolated from solution by means of an external magnetic field and placed in a vacuum oven at a temperature of 80 °C for 14 hours. Then, 1 g CuFe_2_O_4_@GO was dispersed in 20 mL anhydrous toluene under ultrasonication for 2 hours. Moreover, 0.5 mL APTES was added and then allowed to stir for 6 hours *via* reflux. The solid collected was washed with DCM and vacuum dried at 60 °C for 12 hours.

### 4.3. Heterocycle synthesis

#### 4.3.1. Dibenzalacetone (1a–g)

40 mg CuFe_2_O_4_@GO-NH_2_ and 10 mL ethanol were placed in a round bottom flask and sonicated for 15 min to disperse the catalyst. With stirring, benzaldehyde (2 mmol) and acetone (1 mmol) were added to the flask at 0–5 °C (ice bath) for 30 min. The flask was removed from the ice bath and stirred at 25–50 °C depending on the substituents attached to benzaldehyde. When TLC (hexane:EtOAc = 7 : 3) showed complete consumption of benzaldehyde (starting material) and formation of a dibenzalacetone spot, stirring was stopped, and the reaction mixture was cooled to room temperature. The catalyst was removed from the mixture by means of an external magnet or a filter through a Buchner funnel. The cake was washed with EtOH (two times with 5 mL). The filtrate was collected, and its volume was reduced by concentrating it under reduced pressure. 20 mL ethyl acetate was added, and the organic phase was washed with water (two times with 5 mL) and then brine (10 mL). The mixture was dried under Na_2_SO_4_, filtered, evaporated and purified by recrystallization from EtOH/hexane.

##### 4.3.1.1 1,5-Diphenylpenta-1,4-dien-3-one (1a)

Yield: 80%, ^1^H-NMR (400 MHz, DMSO-d_6_): *δ* 7.92 (d, 2H), 7.41–7.21 (m, 10H), 7.18 (d, 2H); ^13^C NMR (400 MHz, DMSO-d_6_): *δ* 191.1 (C-4), 143.1 (C-2, C-6), 135.8 (C-1, C-7), 129.4 (C-11, C-16), 129.0 (C-10, C-12, C-15, C-17), 128.0 (C-3, C-5), 128.1(C-9, C-13, C-14, C-18).

##### 4.3.1.2 1,5-Bis(4-hydroxyphenyl)penta-1,4-dien-3-one (1b)

Yield: 40%, ^1^H-NMR (400 MHz, DMSO-d_6_): *δ* 8.59 (s, 2H), 7.75 (d, 2H), 7.19 (d, 4H), 6.99 (d, 2H), 6.76 (d, 4H); ^13^C NMR (400 MHz, DMSO-d_6_): *δ* 191.1 (C-4), 159.3 (C-11, C-16), 143.1 (C-2, C-6), 129.9 (C-1, C-7), 128.1 (C-3, C-5), 127.6 (C-9, C-13, C-14, C-18), 116.3 (C-10, C-12, C-15, C-17).

##### 4.3.1.3 1,5-Bis(4-nitrophenyl)penta-1,4-dien-3-one (1c)

Yield: 85%, ^1^H-NMR (400 MHz, DMSO-d_6_): *δ* 8.18 (d, 4H), 7.87 (d, 2H), 7.59 (d, 4H), 7.28 (d, 2H); ^13^C NMR (400 MHz, DMSO-d_6_): *δ* 191.1 (C-4), 147.8 (C-11, C-16), 143.1 (C-2, C-6), 142.4 (C-1, C-7), 129.1 (C-3, C-5), 128.1 (C-9, C-13, C-14, C-18), 124.4 (C-10, C-12, C-15, C-17).

##### 4.3.1.4 1,5-Bis(4-hydroxy-3-methoxyphenyl)penta-1,4-dien-3-one (1d)

Yield: 55%, ^1^H-NMR (400 MHz, DMSO-d_6_): *δ* 8.66 (s, 2H), 7.92 (d, 2H), 7.19 (d, 2H), 6.93–6.84 (m, 4H), 6.73 (d,2H), 3.80 (s, 6H); ^13^C NMR (400 MHz, DMSO-d_6_): *δ* 191.1 (C-4), 149.5 (C-11, C-16), 148.5 (C-10, C-17), 143.7 (C-2, C-6), 128.6 (C-3, C-5), 127.5 (C-1, C-7), 123.1 (C-13, C-14), 115.8 (C-12, C-15), 111.7 (C-9,C-18), 56.7 (C-24).

##### 4.3.1.5 1,5-Vis(2-hydroxyphenyl)penta-1,4-dien-3-one (1e)

Yield: 30%, ^1^H-NMR (400 MHz, DMSO-d_6_): *δ* 7.96 (d, 2H), 7.17 (dd, 2H), 7.05 (m, 2H), 6.93–6.65 (m, 6H), 6.82–6.73 (d, 2H); ^13^C NMR (400 MHz, DMSO-d_6_): *δ* 191.1 (C-4), 157.0 (C-9, C-18), 140.8 (C-2, C-6), 131.3 (C-11, C-16), 128.7 (C-3, C-5), 126.5 (C-13, C-14), 121.8 (C-1, C-7), 120.6 (C-12, C-15), 117.7 (C-10, C-17).

##### 4.3.1.6 1,5-Bis(4-(dimethylamino)phenyl)penta-1,4-dien-3-one (1f)

Yield: 35%, ^1^H-NMR (400 MHz, DMSO-d_6_): *δ* 7.72 (d, 2H), 7.15 (d, 4H), 6.93 (d, 2H), 6.59 (d, 4H), 2.89 (s, 12H). ^13^C NMR (400 MHz, DMSO-d_6_): *δ* 191.10 (C-4), 151.12 (C-11, C-16), 143.13 (C-2, C-6), 129.91 (C-1, C-7), 128.11 (C-3, C-5), 123.80 (C-9, C-13, C-14, C-18), 112.70 (C-10, C-12, C-15, C-17), 41.90 (C-21, C-22, C-23, C-24).

##### 4.3.1.7 1,5-Bis(4-chlorophenyl)penta-1,4-dien-3-one(1g)

Yield: 83%, ^1^H-NMR (400 MHz, DMSO-d_6_): *δ* 7.77 (d, 2H, *J* = 12.1 Hz), 7.36–7.21 (m, 8H), 7.03 (d, 2H, *J* = 12.1 Hz); ^13^C NMR (400 MHz, DMSO-d_6_): *δ* 191.10 (C-4), 143.13 (C-11, C-16), 135.42 (C-2, C-6), 135.18 (C-1, C-7), 129.33 (C-3, C-5), 129.16 (C-9, C-13, C-14, C-18), 128.11 (C-10, C-12, C-15, C-17).

#### 4.3.2. [4,5]Dihydropyrazole (IIIab)

To confirm the formation of the cyclized intermediate, a synthetic protocol was set up and isolated for structural elucidation. CuFe_2_O_4_@GO-NH_2_ was dispersed in ethanol under ultrasonic irradiation for 10–15 min, followed by the addition of 2,4-dinitrophenyl hydrazine (2a) (1 mmol) and dibenzalacetone 1,5-bis(4-hydroxyphenyl)penta-1,4-dien-3-one (1b) (1 mmol). The reaction was heated to 40 °C and stirred for 2 h. TLC (hexane : EtOAc = 7 : 3) indicated that compound 1b was consumed, and a new, more polar spot of hydrazone (II) was formed. Now, to achieve the cyclized pyrazoline type intermediate (IIIab), the temperature was increased to 70 °C-80 °C to reflux for 2–3 h with constant stirring. A spot corresponding to a lower polarity in the TLC showed the conversion of hydrazone into a cyclized intermediate. The catalyst was separated through an external magnet and then filtered. The residue was washed with water and ethanol, and the crude product was dried at room temperature.

##### 4.3.2.1 4,4′-(3-(2-(2,4-Dinitrophenyl)hydrazono)penta-1,4-diene-1,5-diyl)diphenol (IIab*)*

Yield: 80% ^1^H-NMR (400 MHz, DMSO-d_6_): *δ* 9.25 (s, 1H, Ar–H), 8.74 (d, 1H, Ar–H), 8.54 (s, 2H, O–H), 7.80 (m, 1H, Ar–H), 7.15 (m, 4H, Ar–H), 6.71 (m, 6H, Ar–H, sp^2^ C–H), 6.55 (m, 2H, sp^2^ C–H); ^13^C NMR (400 MHz, DMSO-d_6_): *δ* 159.3 (C-7, C-26), 142.4 (C-1, C-11), 140.9 (C-14), 135.6 (C-12), 132.3 (C-15), 131.0 (C-15), 129.9 (C-3, C-21), 127.8 (C-5, C-9, C-24, C-28), 127.6 (C-2, C-4, C-22), 123.0 (C-13), 121.8 (C-20), 120.2 (C-16), 116.3 (C-6, C-8, C-25, C-27).

##### 4.3.2.2 4-(1-(3,5-Dinitrophenyl)-3-(4-hydroxystyryl)-4,5-dihydro-1*H*-pyrazol-5-yl)phenol (IIIab)

Yield: 68% ^1^H-NMR (400 MHz, DMSO-d_6_): *δ* 9.01 (t, 1H, Ar–H, *J* = 1.1 Hz), 8.59 (d, 2H, Ar–H, *J* = 1.0 Hz), 8.51 (s, 1H, O–H), 7.15 (m, 4H, Ar–H, *J* = 6.6, 6.1 Hz), 6.82 (m, 2H, Ar–H; 1H, sp^2^ C–H, *J* = 9.0, 3.0 Hz), 6.74 (d, 2H, Ar–H, *J* = 6.0 Hz), 6.09 (d, 1H, sp^2^ C–H*, J* = 12.1 Hz), 4.86 (t, 1H, sp^2^ C–H, *J* = 6.1 Hz), 2.89 (m, 1H, sp^3^ CH_2_, *J* = 10.0, 6.1 Hz), 2.68 (m, 1H, sp^3^ CH_2_, *J* = 9.9, 6.1 Hz); ^13^C NMR (400 MHz, DMSO-d_6_): *δ* 159.3 (C-7), 157.8 (C-26), 156.0 (C-1), 149.7 (C-15, C-17), 141.8 (C-19), 132.9 (C-3), 129.9 (C-5, C-9), 128.8 (C-24, C-28), 127.6 (C-4), 127.5 (C-22), 116.3 (C-6, C-8), 115.7 (C-14), 115.7 (C-25, C-27), 114.4 (C-16, C-18), 70.1 (C-13), 40.5 (C-12).

#### 4.3.3. Fused triazepine (4a–n)

Without isolating the cyclized intermediate (III) and catalyst. The following synthetic protocols were performed in the same reaction mixture leading to the final product (4). Therefore, after mixing of dibenzalacetone (1) and hydrazine (2) in ethanol with stirring under reflux for 3–4 h at 70 °C-80 °C, the mixture was cooled to 40 °C and then isoniazid (3) was added along with 0.1 mL acetic acid directly into the flask with heating to reflux for 2–3 h with constant stirring, followed by the dropwise addition of *N*,*N*-diisopropylethylamine (1.25 mmol, ∼0.22 mL). The progress of the reaction was monitored *via* TLC (EtOAc : hexane = 6 : 4). The appearance of the more polar spot (final product 4) and disappearance of the intermediate evidenced the completion of the reaction. The catalyst was removed using an external magnet, and the remaining solids were filtered and washed with ethanol. 20 mL saturated NaHCO_3_ was added to the filtrate and transferred to a separate funnel, and the organic layer was extracted. The organic layer was washed sequentially with 0.5 M HCl (15 mL) to remove the base, followed by brine (15 mL). The mixture was dried over Na_2_SO_4_, filtered, and concentrated under reduced pressure. The crude was purified by silica column chromatography (hexane:EtOAc = 7 : 3).

##### 4.3.3.1 1-(2,4-Dinitrophenyl)-2,5-diphenyl-8-(pyridin-4-yl)-2,3,5,6,7,8-hexahydro-1*H*-pyrazolo[1,5-d][1,2,4]triazepin-8-ol (4a)

Yield: 53%, m.p.: 275–276 °C; ^1^H-NMR (400 MHz, DMSO-d_6_): *δ* 9.01 (s,1H, Ar–H), 8.59 (s, 1H, Ar–H), 8.51 (m, 2H, pyridine Ar–H), 7.64 (d, 2H, pyridine Ar–H), 7.44–7.08 (m, 11H, Ar–H), 6.25 (s, 1H, O–H), 4.87 (m, 1H, sp^2^ C–H), 4.79 (m, 1H, sp^3^ C–H), 4.14 (t, 1H, sp^3^ C–H), 3.00 (s, 1H, N–H), 2.72 (d, 1H, CH_2_), 2.61 (m, 1H, N–H), 2.36 (d, 1H, CH_2_); ^13^C NMR (400 MHz, DMSO-d_6_): *δ* 151.4 (C-27, C-29), 146.1 (C-13), 144.0 (C-11), 139.4 (C-5), 138.6 (C-26, C-30), 137.8 (C-21), 137.1 (C-22), 132.0 (C-24), 130.7 (C-23), 128.8 (C-16, C-20, C-31, C-37), 128.2 (C-17, C-19, C-32, C-34), 127.8 (C-18, C-33), 124.4 (C-15), 123.7 (C-14), 122.1 (C-25), 101.0 (C-10), 70.9 (C-2, C-6, C-9), 47.8 (C-1); elemental analysis calculated for C_29_H_25_N_7_O_5_: C, 63.15; H, 4.57; N, 17.78; O, 14.50. Found: C, 63.56; H, 4.39; N, 17.56; O, 14.07.

##### 4.3.3.2 4,4′-(1-(2,4-Dinitrophenyl)-8-hydroxy-8-(pyridin-4-yl)-2,3,5,6,7,8-hexahydro-1*H*-pyrazolo[1,5-d][1,2,4]triazepine-2,5-diyl)diphenol (4b)

Yield: 65%; m.p.: 285–286 °C; ^1^H NMR (400 MHz, DMSO-d_6_): *δ* 9.00 (d, 1H, Ar–H), 8.64 (d, 2H, pyridine Ar–H), 8.51–8.43 (m, 2H, O–H), 8.18 (s, 1H, Ar–H), 7.58 (d, 2H, pyridine Ar–H), 7.28 (m, 3H, Ar–H), 7.15 (d, 2H, Ar–H), 6.83 (d, 2H, Ar–H), 6.77 (d, 2H, Ar–H), 4.87 (m, 1H, sp^2^ C–H), 4.83–4.76 (m, 2H, O–H, sp^3^ C–H), 4.14 (t, 1H, sp^3^ C–H), 2.61 (d, 1H, CH_2_), 2.41–2.32 (m, 3H, N–H, CH_2_); ^13^C NMR (400 MHz, DMSO-d_6_): *δ* 157.8 (C-18), 157.4 (C-33), 151.5–151.3 (C-27, C-29), 146.1 (C-13), 144.0 (C-11), 139.4 (C-5), 137.8 (C-21), 132.0 (C-22), 130.7 (C-23), 130.0 (C-14), 129.8 (C-15), 129.3 (C-24), 128.9–128.7 (C-25), 127.5 (C-17, C-19), 124.4 (C-32, C-34), 123.9–123.5 (C-16, C-20), 122.1 (C-26, C-30), 115.7–115.0 (C-31, C-37), 101.0 (C-10), 70.9 (C-2, C-6, C-9), 49.0 (C-1); elemental analysis calculated for C_29_H_25_N_7_O_7_: C, 59.69; H, 4.32; N, 16.80; O, 19.19. Found: C, 59.56; H, 4.39; N, 16.56; O, 19.87.

##### 4.3.3.3 1-(2,4-Dinitrophenyl)-2,5-bis(4-nitrophenyl)-8-(pyridin-4-yl)-2,3,5,6,7,8-hexahydro-1*H*-pyrazolo[1,5-d][1,2,4]triazepin-8-ol (4c)

Yield: 43%; m.p.: 279–280 °C; ^1^H NMR (400 MHz, DMSO-d_6_): *δ* 9.00 (d, 1H, Ar–H), 8.65 (d, 2H, pyridine Ar–H), 8.50 (m, 1H, Ar–H), 8.29 (d, 2H, pyridine Ar–H), 8.18 (d, 2H, Ar–H), 7.67 (m, 4H, Ar–H), 7.50 (d, 2H, Ar–H), 7.30 (d, 1H, Ar–H), 5.97 (s, 1H, O–H), 4.87 (m, 1H, sp^2^ C–H), 4.79 (m, 1H, sp^3^ C–H), 4.14 (t, 1H, sp^3^ C–H), 2.61 (m, 1H, CH_2_), 2.50 (s, 2H, N–H), 2.36 (m, 1H, CH_2_); ^13^C NMR (400 MHz): *δ* 151.4 (C-27, C-29), 147.4 (C-18), 146.6 (C-33), 146.1 (C-13), 145.0 (C-14), 144.0 (C-11), 142.2 (C-15), 139.4 (C-5), 137.8 (C-21), 132.0 (C-24), 130.7 (C-23), 128.2 (C-16, C-20), 127.1 (C-31, C-37), 125.3 (C-32, C-34), 124.4 (C-22), 124.2 (C-17, C-19), 123.7 (C-26, C-30), 122.1 (C-25), 101.0 (C-10), 70.9 (C-2, C-6, C-9)), 41.5 (C-1); elemental analysis calculated for C_29_H_23_N_9_O_9_: C, 54.29; H, 3.61; N, 19.65; O, 22.44. Found: C, 53.96; H, 3.39; N, 19.46; O, 22.07.

##### 4.3.3.4 4,4′-(1-(2,4-Dinitrophenyl)-8-hydroxy-8-(pyridin-4-yl)-2,3,5,6,7,8-hexahydro-1*H*-pyrazolo[1,5-d][1,2,4]triazepine-2,5-diyl)bis(2-methoxyphenol) (4d)

Yield: 55%; m.p.: 284–285 °C; ^1^H NMR (400 MHz, DMSO-d_6_): *δ* 9.16 (s, 1H, Ar–H), 9.02–8.95 (m, 2H, pyridine Ar–H), 8.62 (d, 2H, pyridine Ar–H), 8.47 (dd, 1H, Ar–H), 7.71 (d, 2H, Ar–H), 7.25 (d, 1H, O–H), 6.97–6.85 (m, 2H, Ar–H), 6.84–6.75 (m, 4H, Ar–H, O–H), 5.97 (s, 1H, N–H), 4.87 (m, 1H, sp^2^ C–H), 4.79 (m, 1H, sp^3^ C–H), 4.14 (t, 1H, sp^3^ C–H), 3.81 (d, 6H, sp^3^ C–H), 2.65–2.54 (d, 1H, CH_2_), 2.42–2.31 (m, 2H, N–H, CH_2_); ^13^C NMR (400 MHz, DMSO-d_6_): *δ* 151.4 (C-27, C-29), 147.8 (C-34), 147.4 (C-18), 147.0 (C-17), 146.6 (C-33), 146.1 (C-13), 144.0 (C-11), 139.4 (C-5), 137.8 (C-21), 132.0 (C-24), 130.7 (C-23), 130.2 (C-14), 129.3 (C-15), 124.4 (C-22), 123.7 (C-31), 122.1 (C-25), 120.9 (C-20), 120.5 (C-26, C-30), 115.4 (C-19), 114.8 (C-32), 111.5 (C-37), 111.3 (C-16), 101.0 (C-10), 70.9 (C-2, C-6, C-9), 56.7 (C-40, C-41), 49.3 (C-1); elemental analysis calculated for C_31_H_29_N_7_O_9_ C, 57.85; H, 4.54; N, 15.23; O, 22.37. Found: C, 57.96; H, 4.49; N, 15.36; O, 22.26

##### 4.3.3.5 2,2′-(1-(2,4-Dinitrophenyl)-8-hydroxy-8-(pyridin-4-yl)-2,3,5,6,7,8-hexahydro-1*H*-pyrazolo[1,5-*d*][1,2,4]triazepine-2,5-diyl)diphenol (4e)

Yield: 42%; m.p.: 289 °C-290 °C; ^1^H NMR (400 MHz, DMSO-d_6_): *δ* 8.80 (d, 1H, Ar–H), 8.44 (d, 2H, pyridine Ar–H), 8.26 (m, 1H, Ar–H), 8.09 (s, 1H, Ar–H), 7.49 (m, 1H, O–H), 7.14 (m, 2H, pyridine Ar–H), 7.05 (m, 3H, Ar–H), 6.43–6.65 (m, 5H), 6.79–6.58 (broad, 2H, O–H), 4.76 (m, 1H, sp^2^ C–H), 4.68 (d, 1H, sp^3^ C–H), 4.22 (s, 1H, N–H), 4.08–4.00 (t, 1H, sp^3^ C–H), 2.44 (m, 1H, CH_2_), 2.35–2.26 (m, 2H, N–H, CH_2_); ^13^C NMR (400 MHz, DMSO-d_6_): *δ* 157.7 (C-31), 156.8 (C-16), 151.4 (C-27, C-29), 146.1 (C-13), 144.0 (C-11), 141.3 (C-5), 137.8 (C-21), 132.0 (C-24), 130.7 (C-23), 129.9 (C-20), 129.7 (C-37), 129.4 (C-18), 128.1 (C-33), 124.4 (C-22), 123.7 (C-26, C-30), 123.7–123.6 (C-14), 122.1 (C-25), 121.0 (C-34), 120.6 (C-19), 119.9 (C-15), 118.4 (C-32), 116.0 (C-17), 101.7 (C-10), 75.0 (C-5, C-9), 65.4 (C-2), 40.9 (C-1); elemental analysis calculated for C_29_H_25_N_7_O_7_ C, 59.69; H, 4.32; N, 16.80; O, 19.19. Found: C, 59.56; H, 4.29; N, 16.46; O, 19.15.

##### 4.3.3.6 2,5-bis(4-(Dimethylamino)phenyl)-1-(2,4-dinitrophenyl)-8-(pyridin-4-yl)-2,3,5,6,7,8-hexahydro-1*H*-pyrazolo[1,5-d][1,2,4]triazepin-8-ol (4f)

Yield: 58%; m.p.: 283–284 °C; ^1^H NMR (400 MHz, DMSO-d_6_): *δ* 9.00 (d, 1H, Ar–H), 8.65 (m, 2H, pyridine Ar–H), 8.46 (m, 1H, Ar–H), 7.65 (d, 2H, pyridine Ar–H), 7.38–7.21 (m, 3H, Ar–H), 7.13 (m, 2H, Ar–H), 6.68 (m, 2H, Ar–H), 6.60 (m, 2H, Ar–H), 4.87 (m, 1H, sp^2^ C–H), 4.79 (m, 2H, O–H, sp^3^ C–H), 4.14 (t, 1H, sp^3^ C–H), 2.88 (d, 12H), 2.65–2.56 (m, 2H, N–H, CH_2_), 2.43–2.32 (m, 2H, N–H, CH_2_); ^13^C NMR (400 MHz, DMSO-d_6_): *δ* 152.1 (C-18), 151.4 (C-27, C-29), 150.6 (C-33), 146.1 (C-13), 144.0 (C-11), 139.4 (C-5), 137.8 (C-21), 132.0 (C-24), 130.7 (C-23), 128.9 (C-31, C-37), 128.3 (C-16, C-20), 126.5 (C-14), 124.4 (C-22), 123.7 (C-26, C-30), 123.0 (C-15), 122.1 (C-25), 114.2 (C-32, C-34), 112.7 (C-17, C-19), 101.0 (C-10), 70.9 (C-2, C-5, C-9), 50.3 (C-40, C-41, C-42, C-43), 49.8 (C-1); elemental analysis calculated for C_33_H_35_N_9_O_5_ C, 62.15; H, 5.53; N, 19.77; O, 12.54. Found: C, 62.04; H, 5.12; N, 19.23; O, 12.34.

##### 4.3.3.7 2,5-Bis(4-chlorophenyl)-1-(2,4-dinitrophenyl)-8-(pyridin-4-yl)-2,3,5,6,7,8-hexahydro-1*H*-pyrazolo[1,5-*d*][1,2,4]triazepin-8-ol (4g)

Yield: 48%; m.p.: 276–277 °C; ^1^H NMR (400 MHz, DMSO-d_6_): *δ* 8.99 (d, 1H, Ar–H), 8.60 (d, 2H, pyridine Ar–H), 8.47–8.40 (m, 1H, Ar–H), 7.81 (m, 1H, Ar–H), 7.73 (d, 2H, pyridine Ar–H), 7.31–7.15 (m, 8H, Ar–H), 4.87 (m, 1H, sp^2^ C–H), 4.76 (m, 1H, sp^3^ C–H), 4.14 (t, 1H, sp^3^ C–H), 3.44 (s, 1H, O–H), 2.71 (m,1H, N–H), 2.61 (m, 1H, CH_2_), 2.46 (m, 1H, N–H), 2.36 (m, 1H, CH_2_); ^13^C NMR (400 MHz, DMSO-d_6_): *δ* 151.4 (C-27, C-29), 146.1 (C-13), 144.0 (C-11), 139.4 (C-5), 137.8 (C-21), 137.4 (C-14), 136.2 (C-15), 133.4 (C-18), 133.1 (C-33), 132.0 (C-24), 130.7 (C-23), 130.0 (C-16, C-20), 129.6 (C-31, C-37), 129.2 (C-32, C-34), 128.7 (C-17, C-19), 124.4 (C-22), 123.7 (C-26, C-30), 122.1 (C-25), 101.0 (C-10), 70.9 (C-2, C-6, C-9), 41.5 (C-1); elemental analysis calculated for C_29_H_23_Cl_2_N_7_O_5_ C, 56.14; H, 3.74; Cl, 11.43; N, 15.80; O, 12.89. Found: C, 57.04; H, 4.12; N, 16.14; O, 13.01.

##### 4.3.3.8 8-Hydroxy-2,5-diphenyl-8-(pyridin-4-yl)-2,3,5,6,7,8-hexahydro-1*H*-pyrazolo[1,5-*d*][1,2,4] triazepine-1-carboxamide (4h)

Yield: 53%; m.p.: 273–274 °C; ^1^H NMR (400 MHz, DMSO-d_6_): *δ* 8.59 (d, 2H, pyridine Ar–H), 7.60 (d, 2H, pyridine Ar–H), 7.41–7.09 (m, 10H, Ar–H), 6.18 (s, 2H, N–H), 4.95–4.84 (m, 2H, sp^2^ C–H, sp^3^ C–H), 4.79 (d, 1H, sp^3^ C–H), 3.28 (s, 1H, O–H), 2.63 (m, 1H), 2.38 (m, 1H), 2.16 (s, 1H); ^13^C NMR (400 MHz, DMSO-d_6_): *δ* 158.8 (C-11), 151.4 (C-22, C-24), 146.1 (C-13), 139.5 (C-5), 138.6 (C-14), 137.1 (C-15), 128.8 (C-17, C-19, C-27, C-29), 128.2 (C-16, C-20), 127.8 (C-26, C-30), 127.8 (C-18, C-28), 123.7 (C-21, C-25), 101.6 (C-10), 65.6 (C-2, C-6, C-9), 47.0 (C-1); elemental analysis calculated for C_24_H_24_N_6_O_2_ C, 67.27; H, 5.65; N, 19.61; O, 7.47. Found: C, 67.06; H, 5.45; N, 19.23; O, 7.21.

##### 4.3.3.9 8-Hydroxy-2,5-bis(4-hydroxyphenyl)-8-(pyridin-4-yl)-2,3,5,6,7,8-hexahydro-1*H*-pyrazolo[1,5-*d*][1,2,4]triazepine-1-carboxamide (4i)

Yield: 65%; m.p.: 286–287 °C; ^1^H NMR (400 MHz, DMSO-d_6_): *δ* 8.55 (d, 2H, pyridine Ar–H), 8.44 (s, 1H, sp^2^ O–H), 7.50 (d, 2H, pyridine Ar–H), 7.22–7.14 (m, 4H, Ar–H), 7.09 (s, 2H, Ar–H), 6.82 (m, 4H, NH_2,_ Ar–H), 6.56 (s, 1H, O–H), 4.94–4.84 (m, 2H, sp^2^ C–H, sp^3^ C–H), 4.79 (s, 1H, sp^3^ C–H), 2.96 (s, 1H, N–H), 2.67–2.57 (m, 2H, N–H, CH_2_), 2.38 (m, 1H, CH_2_); ^13^C NMR (400 MHz, DMSO-d_6_): *δ* 158.8 (C-11), 157.8 (C-18), 157.4 (C-28), 151.4 (C-22, C-24), 146.1 (C-13), 139.5 (C-5), 129.9 (C-16, C-20), 129.3 (C-14), 128.8 (C-26, C-30), 127.5 (C-15), 123.7 (C-21, C-25), 115.7 (C-27, C-29), 115.0 (C-17, C-19), 101.6 (C-10), 65.6 (C-2, C-6, C-9), 48.67 (C-1); elemental analysis calculated for C_24_H_24_N_6_O_4_ C, 62.60; H, 5.25; N, 18.25; O, 13.90. Found: C, 63.16; H, 5.23; N, 18.32; O, 13.12.

##### 4.3.3.10 8-Hydroxy-2,5-bis(4-nitrophenyl)-8-(pyridin-4-yl)-2,3,5,6,7,8-hexahydro-1*H*-pyrazolo[1,5-*d*][1,2,4]triazepine-1-carboxamide (4j)

Yield: 43%; m.p.: 286–287 °C; ^1^H NMR (400 MHz, DMSO-d_6_): *δ* 8.62 (d, 2H, pyridine Ar–H), 8.27 (d, 2H, pyridine Ar–H), 8.23 (d, 2H, Ar–H), 7.68–7.52 (m, 6H, Ar–H), 5.94 (s, 2H, NH_2_), 5.44 (s, 1H, O–H), 4.95–4.84 (m, 2H, sp^2^ C–H, sp^3^ C–H), 4.79 (d, 1H, sp^3^ C–H), 2.69–2.58 (m, 2H, N–H, CH_2_), 2.55 (s, 1H, N–H), 2.38 (m, 1H, CH_2_); ^13^C NMR (400 MHz, DMSO-d_6_): *δ* 158.8 (C-11), 151.4 (C-22, C-24), 147.4 (C-18), 146.6 (C-28), 146.1 (13), 145.0 (C-14), 142.2 (C-15), 139.5 (C-5), 128.2 (C-16, C-20), 127.1 (C-26, C-30), 125.3 (C-27, C-29), 124.2 (C-17, C-19), 123.7 (C-21, C-25), 101.6 (C-10), 65.6 (C-2, C-6, C-9), 47.8 (C-1); elemental analysis calculated for C_24_H_22_N_8_O_6_ C, 55.60; H, 4.28; N, 21.61; O, 18.51. Found: C, 55.32; H, 4.16; N, 21.43; O, 18.33.

##### 4.3.3.11 8-Hydroxy-2,5-bis(4-hydroxy-3-methoxyphenyl)-8-(pyridin-4-yl)-2,3,5,6,7,8-hexahydro-1*H*-pyrazolo[1,5-*d*][1,2,4]triazepine-1-carboxamide (4k)

Yield: 55%; m.p.: 277–278 °C; ^1^H NMR (400 MHz, DMSO-d_6_): *δ* 8.95 (s, 1H, Ar–H), 8.62 (d, 2H, pyridine Ar–H), 8.30 (s, 1H, Ar–H), 7.57 (d, 2H, pyridine Ar–H), 7.04–6.89 (m, 2H, O–H), 6.89–6.67 (m, 7H, Ar–H, NH_2_), 4.96–4.84 (m, 2H, sp^2^ C–H, sp^3^ C–H), 4.79 (d, 1H, sp^3^ C–H), 3.87–3.79 (m, 6H, CH_3_), 3.16 (s, 1H, N–H), 2.63 (m, 1H, CH_2_), 2.62 (m, 1H, N–H), 2.38 (m, 1H, CH_2_); ^13^C NMR (400 MHz, DMSO-d_6_): *δ* 158.8 (C-11), 151.4 (C-22, C-24), 147.8 (C-29), 147.4 (C-l8), 147.0 (C-28), 146.6 (C-17), 146.1 (C-13), 139.5 (C-5), 130.2 (C-14), 129.3 (C-15), 123.7 (C-21, C-25), 120.9 (C-20), 120.5 (C-26), 115.4 (C-19), 114.8 (C-27), 111.5 (C-16), 111.3 (C-30), 101.6 (C-10), 64.8 (C-2, C-6, C-9), 56.7 (C-33, C-34), 47.2 (C-1); elemental analysis calculated for C_26_H_28_N_6_O_6_ C, 59.99; H, 5.42; N, 16.14; O, 18.44. Found: C, 59.15; H, 4.65; N, 16.32; O, 18.57.

##### 4.3.3.12 8-Hydroxy-2,5-bis(2-hydroxyphenyl)-8-(pyridin-4-yl)-2,3,5,6,7,8-hexahydro-1*H*-pyrazolo[1,5-*d*][1,2,4]triazepine-1-carboxamide (4l)

Yield: 42%; m.p.: 281–282 °C; ^1^H NMR (400 MHz, DMSO-d_6_): yield: 45%; m.p.: 281–282 °C; ^1^H NMR (400 MHz, DMSO-d_6_): *δ* 8.49 (d, 2H, pyridine Ar–H), 8.34 (s, 1H, O–H), 7.49 (d, 2H, Ar–H), 7.20–7.04 (m, 4H, Ar–H), 6.93–6.80 (m, 3H, Ar–H), 6.76 (d, 1H, O–H), 5.91 (s, 2H, NH_2_), 4.95–4.84 (m, 2H, sp^2^ C–H, sp^3^ C–H), 4.79 (d, 1H, sp^3^ C–H), 3.13 (s, 1H, N–H), 2.63 (m, 1H, CH_2_), 2.55 (s, 1H, N–H), 2.38 (m, 1H, CH_2_); ^13^C NMR (400 MHz, DMSO-d_6_): *δ* 158.8 (C-11), 157.7 (C-26), 156.8 (C-16), 151.4 (C-22, C-24), 146.1 (C-13), 141.5 (C-5), 129.9 (C-20), 129.7 (C-30), 129.4 (C-18), 128.1 (C-28), 123.7 (C-21, C-25), 123.7–123.6 (C-14), 121.0 (C-29), 120.6 (C-19), 119.9 (C-15), 118.4 (C-27), 116.0 (C-17), 102.3 (C-10), 64.1 (C-2, C-6, C-9), 47.0 (C-1); elemental analysis calculated for C_26_H_28_N_6_O_6_ C, 59.99; H, 5.42; N, 16.14; O, 18.44. Found: C, 59.43; H, 5.44; N, 16.32; O, 18.66.

##### 4.3.3.13 2,5-Bis(4-(dimethylamino)phenyl)-8-hydroxy-8-(pyridin-4-yl)-2,3,5,6,7,8-hexahydro-1*H*-pyrazolo[1,5-*d*][1,2,4]triazepine-1-carboxamide (4m)

Yield: 58%; m.p.: 284–285 °C; ^1^H NMR (400 MHz, DMSO-d_6_): *δ* 8.61 (d, 2H, pyridine Ar–H), 7.53 (d, 2H, pyridine Ar–H), 7.17 (d, 2H, Ar–H), 7.09–6.98 (m, 4H, Ar–H), 6.67 (d, 2H, NH_2_), 6.60 (d, 3H, Ar–H, O–H), 4.96–4.84 (m, 2H, sp^2^ C–H, sp^3^ C–H), 4.79 (d, 1H, sp^3^ C–H), 3.17 (s, 1H, N–H), 2.88 (d, 12H, CH_3_), 2.63 (d, 1H, CH_2_), 2.38 (d, 1H, CH_2_); ^13^C NMR (400 MHz DMSO-d_6_): NMR (400 MHz) *δ* 158.8 (C-11), 152.1 (C-18), 151.4 (C-22, C-24), 150.6 (C-28), 146.1 (C-13), 139.5 (C-5), 128.9 (C-16, C-20), 128.3 (C-26, C-30), 126.5 (C-14), 123.7 (C-21, C-25), 123.0 (C-15), 114.2 (C-27, C-29), 112.7 (C-17, C-19), 101.6 (C-10), 65.6 (C-2, C-6, C-9), 49.7 (C-35, C-36, C-37, C-38), 48.6 (C-1); elemental analysis calculated for C_28_H_34_N_8_O_2_ C, 65.35; H, 6.66; N, 21.77; O, 6.22. Found: C, 65.11; H, 6.78; N, 22.34; O, 7.36.

##### 4.3.3.14 2,5-Bis(4-chlorophenyl)-8-hydroxy-8-(pyridin-4-yl)-2,3,5,6,7,8-hexahydro-1*H*-pyrazolo[1,5-*d*][1,2,4]triazepine-1-carboxamide (4n)

Yield: 48%; m.p.: 273–274 °C; ^1^H NMR (400 MHz, DMSO-d_6_): *δ* 8.55 (d, 2H, pyridine Ar–H), 7.55 (d, 2H, pyridine Ar–H), 7.43–7.22 (m, 8H, Ar–H), 7.13–7.02 (m, 2H, NH_2_), 6.64 (s, 1H, O–H), 5.57 (s, 1H, N–H), 4.96–4.71 (m, 3H, sp^2^ C–H, sp^3^ C–H, sp^3^ C–H), 2.95 (s, 1H, N–H), 2.63 (d, 1H, CH_2_), 2.38 (d, 1H, CH_2_).^13^C NMR (400 MHz, DMSO-d_6_): *δ* 158.8 (C-11), 151.4 (C-22, C-24), 146.1 (C-13), 139.5 (C-5), 137.4 (C-14), 136.2 (C-15), 133.4 (C-28), 133.1 (C-18), 130.0 (C-16, C-20), 129.6 (C-26, C-30), 129.2 (C-27, C-29), 128.7 (C-17, C-19), 123.7 (C-21, C-25), 101.6 (C-10), 65.6 (C-2, C-6, C-9), 47.4 (C-1); elemental analysis calculated for C_24_H_22_Cl_2_N_6_O_2_ C, 57.96; H, 4.46; Cl, 14.26; N, 16.90; O, 6.43. Found: C, 57.22; H, 4.36; N, 16.76; O, 7.53.

### 4.4. Biological assays

The α-amylase and α-glucosidase enzyme assays were conducted according to reported literature protocols.^33,34^ The optical densities were measured by applying a UV-visible spectrophotometer.

### 4.5. *In silico* docking studies

The molecular docking studies were conducted in Biovia Discovery Studio (2019). All the optimized ligands (4a-4p) and proteins went through energy minimization using a CHARMm (Chemistry at Harvard Macromolecular Mechanics)-based smart minimizer. Minimization took place by performing 2000 steps of steepest descent, followed by 1000 steps of the conjugate gradient with an RMSD gradient of 0.01 kcal mol^−1^. The X-ray crystal structures of the protein with PDB ID: 2QV4 and 3W37 were retrieved from RCSB Protein Data Bank (https://www.rcsb.org/). The proteins were prepared by removing water molecules and adding appropriate charges by following the standard protocols.^42^ The receptor grid was identified by redocking the co-crystal ligand with the receptor protein. The binding site sphere was set with a radius of 10.5387 Å and *x*, y, and *z* dimensions of 48.1361 Å, 26.092 Å, and 12.3847 Å for 2QV4 and a radius of 0.108773 Å and *x*, *y*, and *z* dimensions of −1.91698 Å, −23.0532 Å, and 9.89006 Å, respectively. The docking was performed using a cdocker (charm-based DOCKER) algorithm. Out of the 10 docked binding poses, the best mode was chosen for each compound, and interactions were analyzed using 2D and 3D protein-ligand complex diagrams. The binding energies and complex stability were compared with the reference co-crystal ligand acarbose.

### 4.6. Pharmacokinetic properties

Drug-likeness profiles of the synthesized compounds and acarbose were screened by Lipinski's Rule of Five using molecular properties. All these molecules with the best pose were subjected to TOPKAT descriptor toxicity and ADMET analysis using the Biovia Discovery Studio (2019).

### 4.7. Density functional theory studies

The molecules were optimized at the B3LYP level of theory with a 6-311G++(d,p) basis set using the Gaussian 09 program suite. The energies of HOMO and LUMO were determined. The reactivity and stability of the compounds were appraised by calculating the following parameters such as chemical hardness (*η*), chemical potential (*µ*), and electrophilicity (*ω*). Furthermore, for a broad level understanding of electronic density dispersion, the molecular electrostatic potential (MEP) surface contour was determined.

## Conflicts of interest

There are no conflicts to declare.

## Supplementary Material

RA-016-D6RA00293E-s001

## Data Availability

All data supporting the findings of this study, including the molecular docking results, assay-based *in vitro* analyses, *ab initio* calculations, pharmacokinetics properties and experimental characterization data (NMR, IR, and XRD) are provided in the electronic supplementary information (SI). Supplementary information is available. See DOI: https://doi.org/10.1039/d6ra00293e.
