# Supplementary material for: GO/APTES-mediated bifunctional CuFe2O4@GO-NH2-facilitated synthesis of pyrazolo-triazepine scaffolds as a potent post-prandial antidiabetic agent against dual α-amylase and α-glucosidase enzymes
Source: RSC Adv. 2026 May 18;16(29):26249–72. doi: 10.1039/d6ra00293e (PMC13185714; doi:10.1039/d6ra00293e)
Supplement: RA-016-D6RA00293E-s001 [file RA-016-D6RA00293E-s001.pdf]

Supplementary Information (SI) for RSC Advances.
This journal is © The Royal Society of Chemistry 2026

# Supporting information

## GO/APTES mediated bifunctional $CuFe_2O_4$@GO-$NH_2$ facilitated synthesis of pyrazolo-triazepine scaffolds as potent post prandial antidiabetic agent against dual α-amylase and α-glucosidase enzymes

Romica Jain, Ashok Kumar, Pratibha Sharma*
*School of Chemical Science, Devi Ahilya University, Indore 452001 (M.P.), India*
*Corresponding author email-* drpratibhasharma@yahoo.com

# Table Contents

**-Figure S1-S28. The spectra of pyrazole fused [1,2,4]triazepine scaffolds (4a-n)**

**-Figure S29-S42. The spectra of dibenzalacetone (1a-g)**

**-Figure S43-44. The spectra of [4,5]dihydropyrazole (IIIab)**

**-Figure S45-46. The spectra of hydrazone i.e. 4,4'-(3-(2-(2,4-dinitrophenyl)hydrazono)penta-1,4-diene-1,5-diyl)diphenol (IIab)**

**-Figure S47. 2D (left) and 3D (right) representations of closest binding interaction between synthesized ligands (4a-4n) and receptor protein 2QV4**

**-Figure S48. 2D (left) and 3D (right) representations of closest binding interaction between synthesized ligands (4a-4n) and receptor protein 3W37**

**-Figure S49. Molecular orbitals diagram (HOMO; bottom and LUMO; top) with HOMO-LUMO energy gap.**

**-Table S1. Absorbance and corresponding % Inhibition of the tested compounds (4a-4n) against α-amylase at different concentrations.**

**-Table S2. Absorbance and corresponding % Inhibition of the tested compounds (4a-4n) against α-amylase at different concentrations.**

**Figure S1.** $^{1}$H NMR of 1-(2,4-dinitrophenyl)-2,5-diphenyl-8-(pyridin-4-yl)-2,3,5,6,7,8-hexahydro-1H-pyrazolo[1,5-d][1,2,4]triazepin-8-ol (4a)

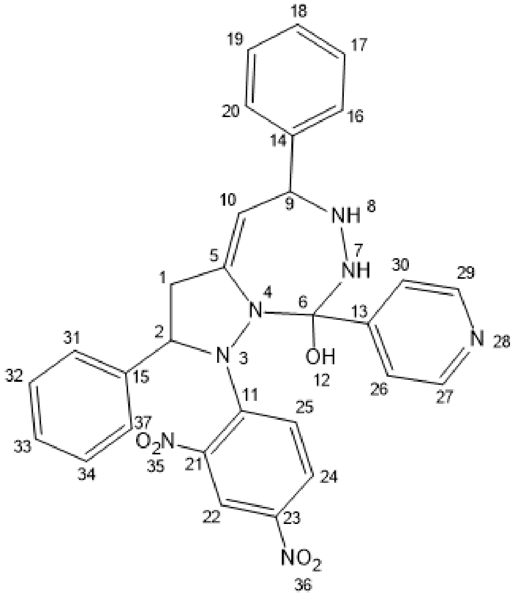


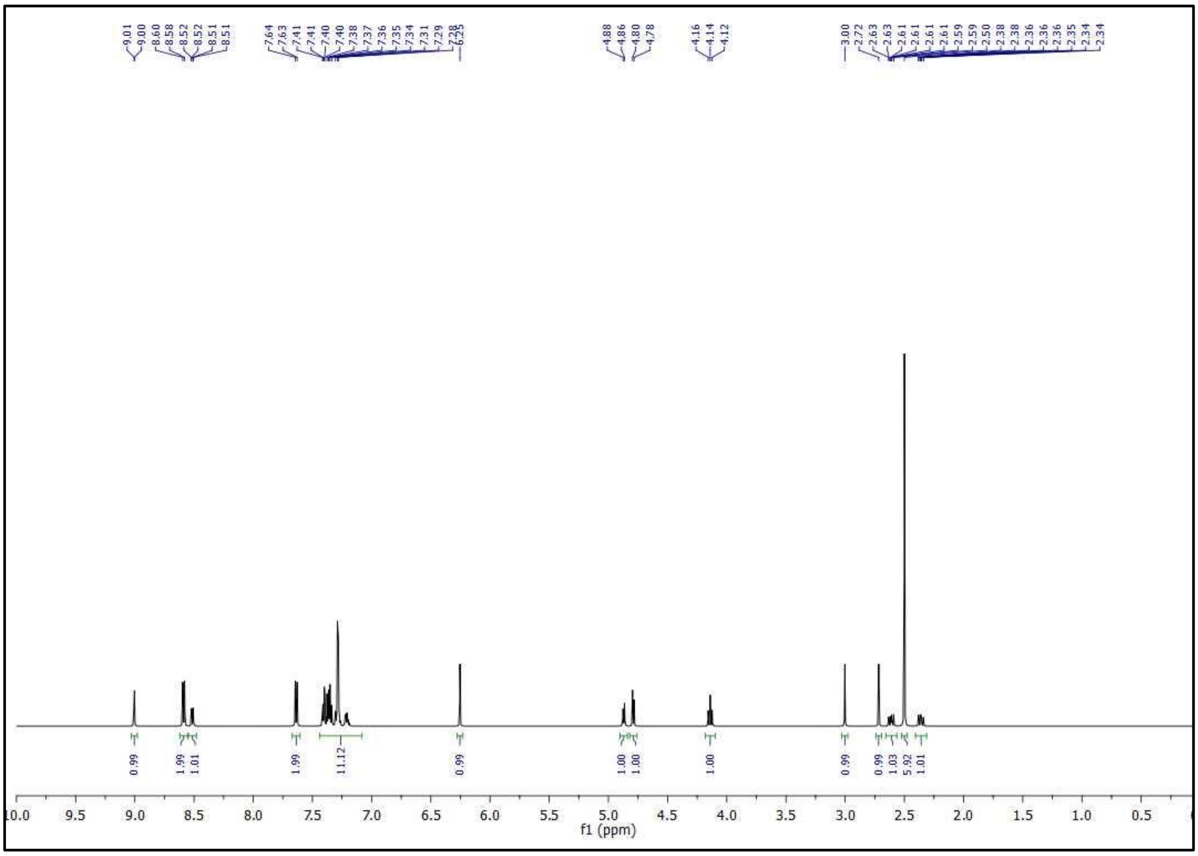


**Figure S2.** $^{13}$C NMR of 1-(2,4-dinitrophenyl)-2,5-diphenyl-8-(pyridin-4-yl)-2,3,5,6,7,8-hexahydro-1H-pyrazolo[1,5-d][1,2,4]triazepin-8-ol (4a)

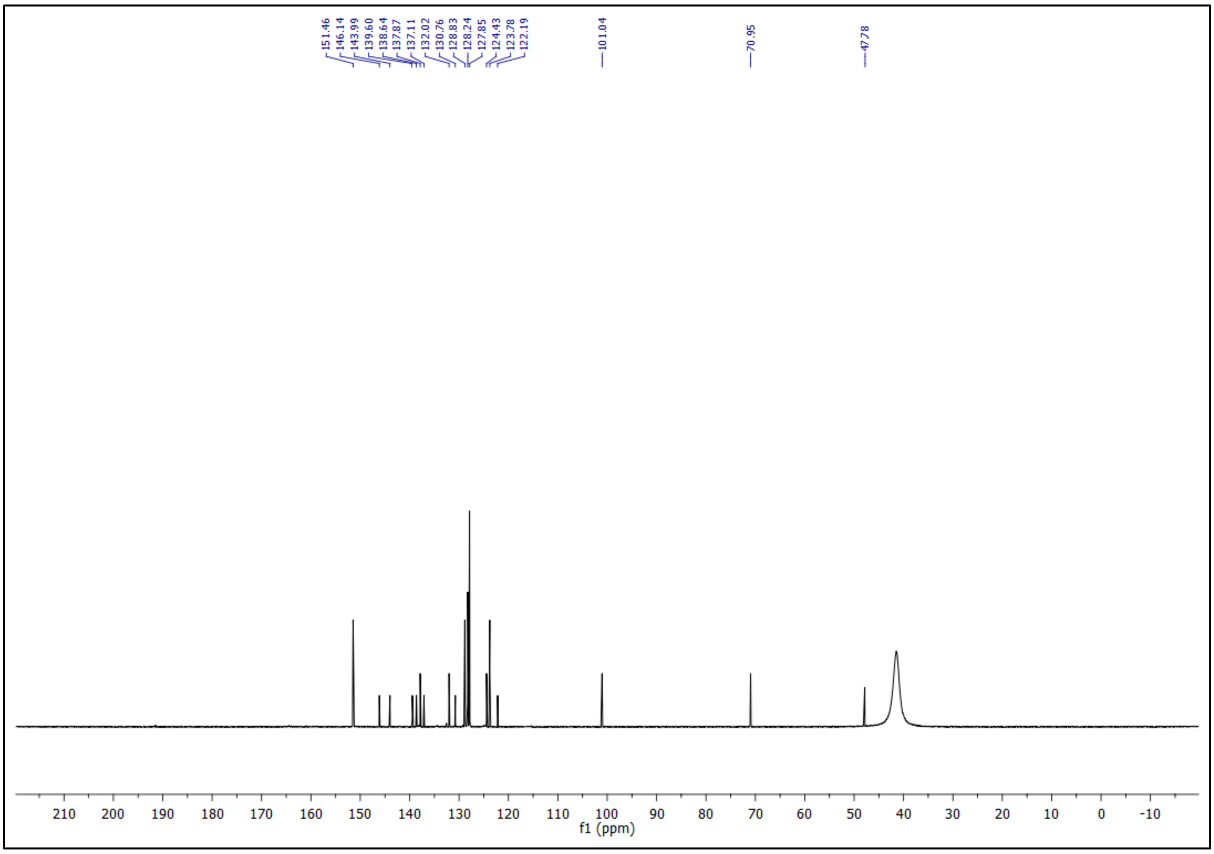


**Figure S3.** $^{1}$H NMR of 4,4'-(1-(2,4-dinitrophenyl)-8-hydroxy-8-(pyridin-4-yl)-2,3,5,6,7,8-hexahydro-1H-pyrazolo[1, 5-d][1,2,4]triazepine-2,5-diyl)diphenol (4b)

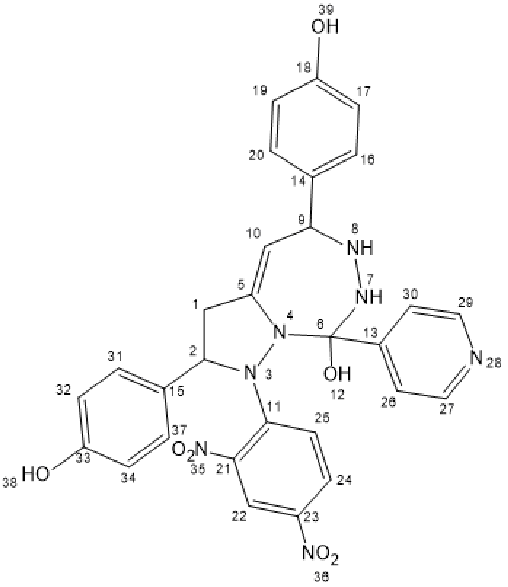

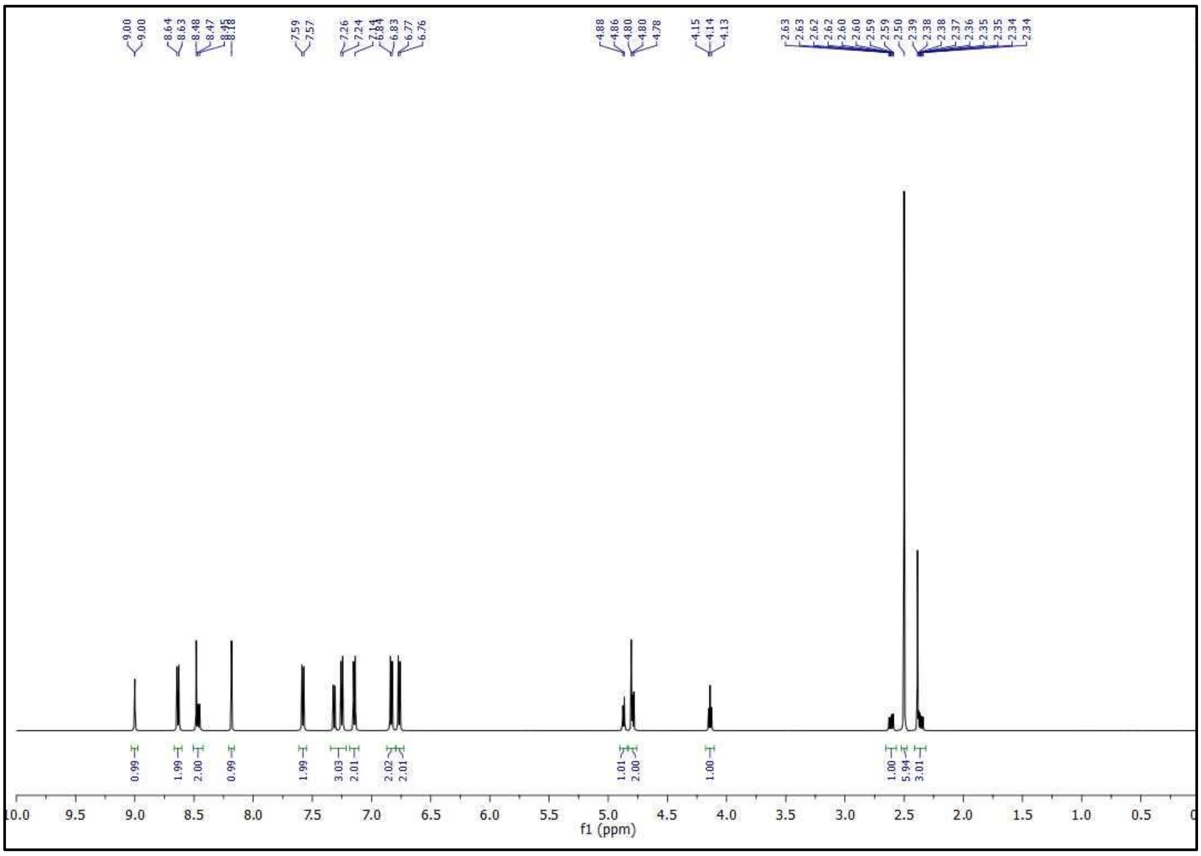


**Figure S4.** $^{13}$C NMR of 4,4'-(1-(2,4-dinitrophenyl)-8-hydroxy-8-(pyridin-4-yl)-2,3,5,6,7,8-hexahydro-1H-pyrazolo[1, 5-d][1,2,4]triazepine-2,5-diyl)diphenol (4b)

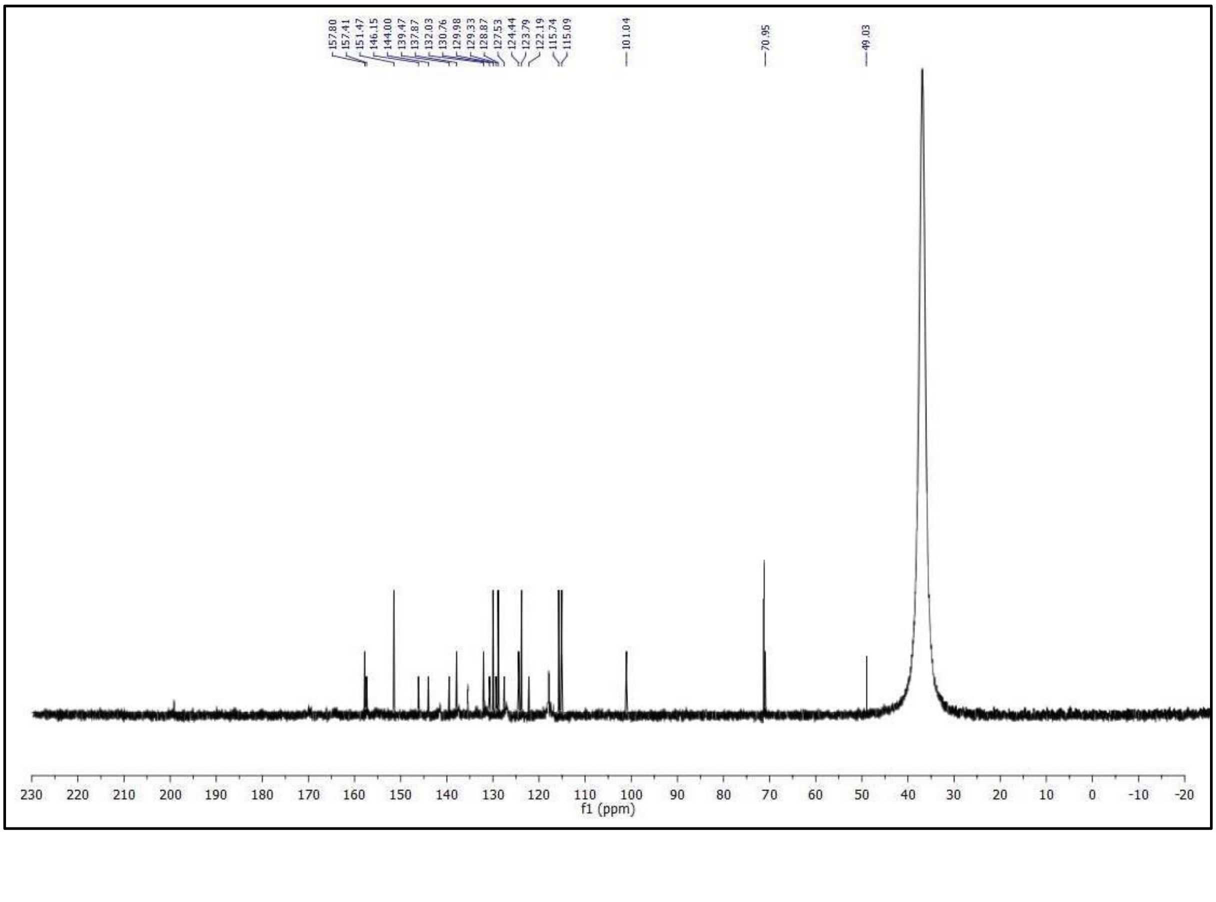

**Figure S5.** $^{1}$H NMR of 1-(2,4-dinitrophenyl)-2,5-bis(4-nitrophenyl)-8-(pyridin-4-yl)-2,3,5,6,7,8-hexahydro-1H-pyrazolo[1,5-d][1,2,4]triazepin-8-ol (4c)

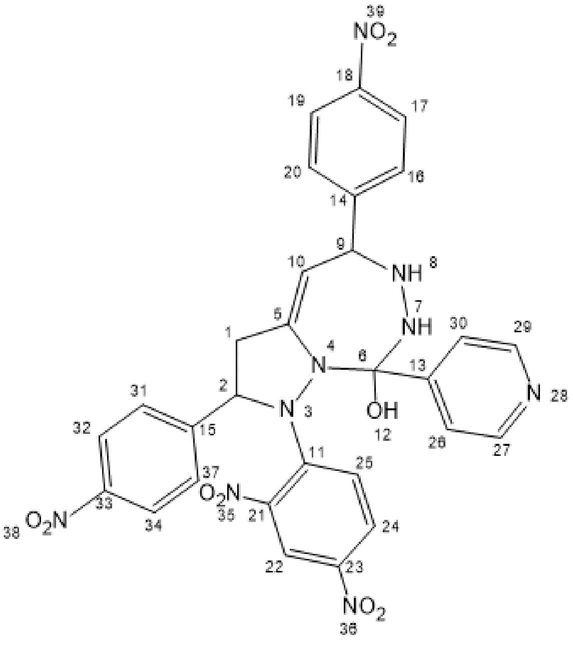


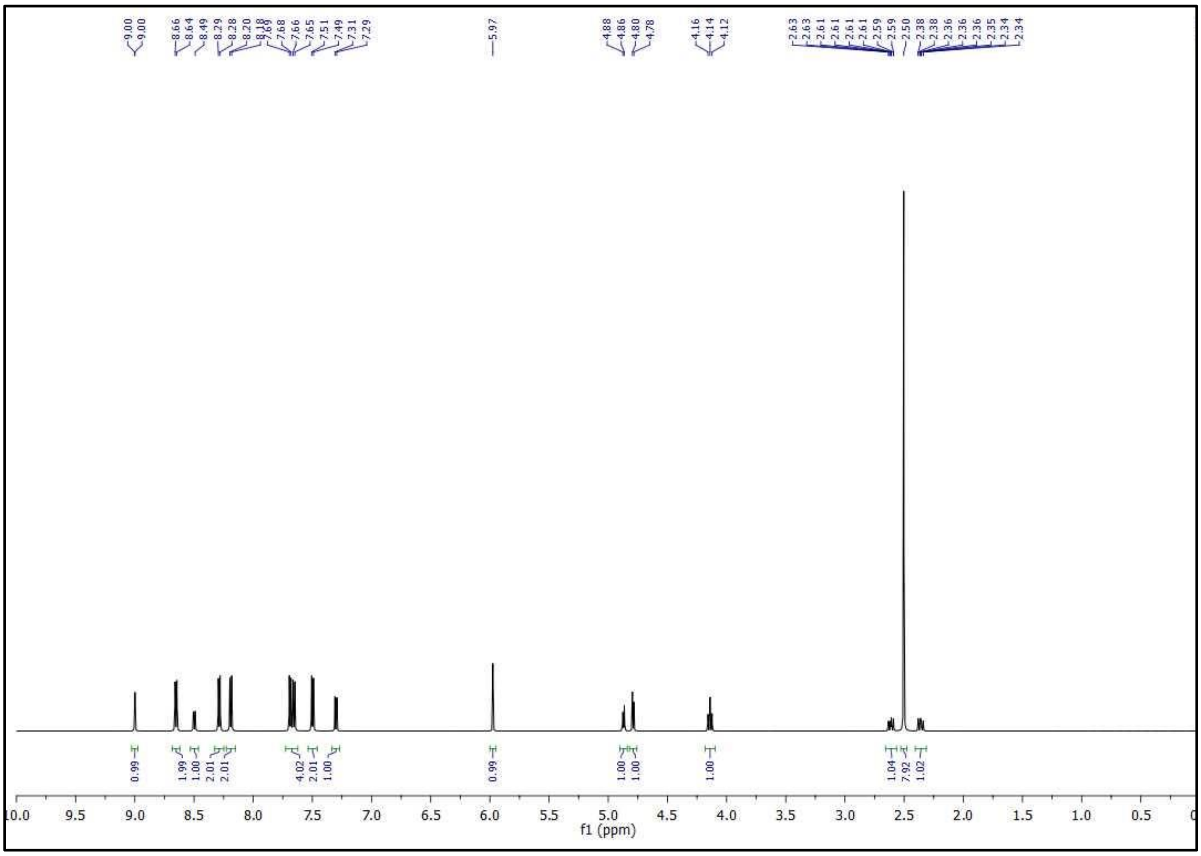


**Figure S6.** $^{13}$C NMR of 1-(2,4-dinitrophenyl)-2,5-bis(4-nitrophenyl)-8-(pyridin-4-yl)-2,3,5,6,7,8-hexahydro-1H-pyrazolo[1,5-d][1,2,4]triazepin-8-ol (4c)

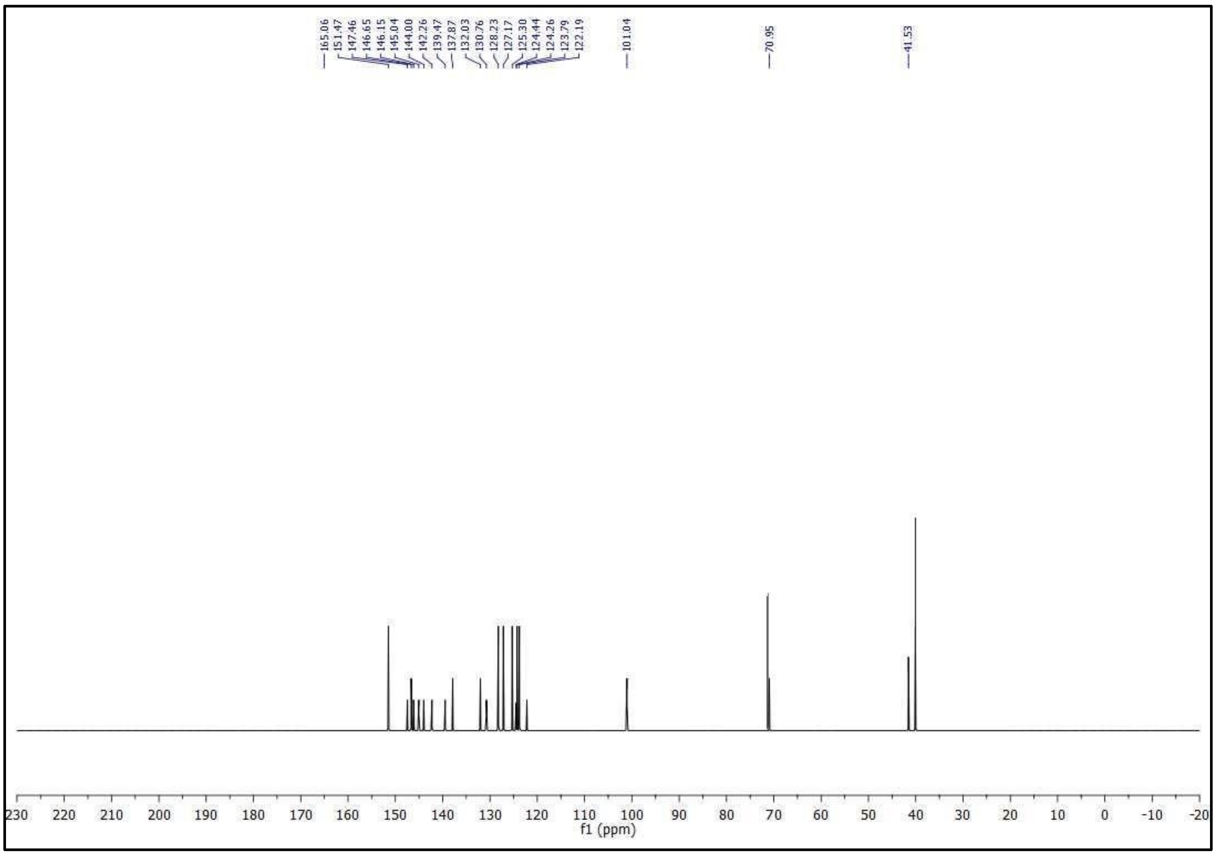


**Figure S7.** $^{1}$H NMR of 4,4'-(1-(2,4-dinitrophenyl)-8-hydroxy-8-(pyridin-4-yl)-2,3,5,6,7,8-hexahydro-1H-pyrazolo[1, 5-d][1,2,4]triazepine-2,5-diyl)bis(2-methoxyphenol) (4d)

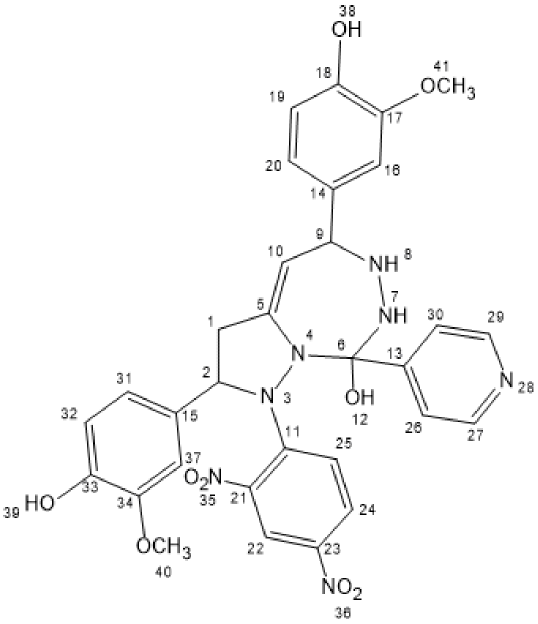

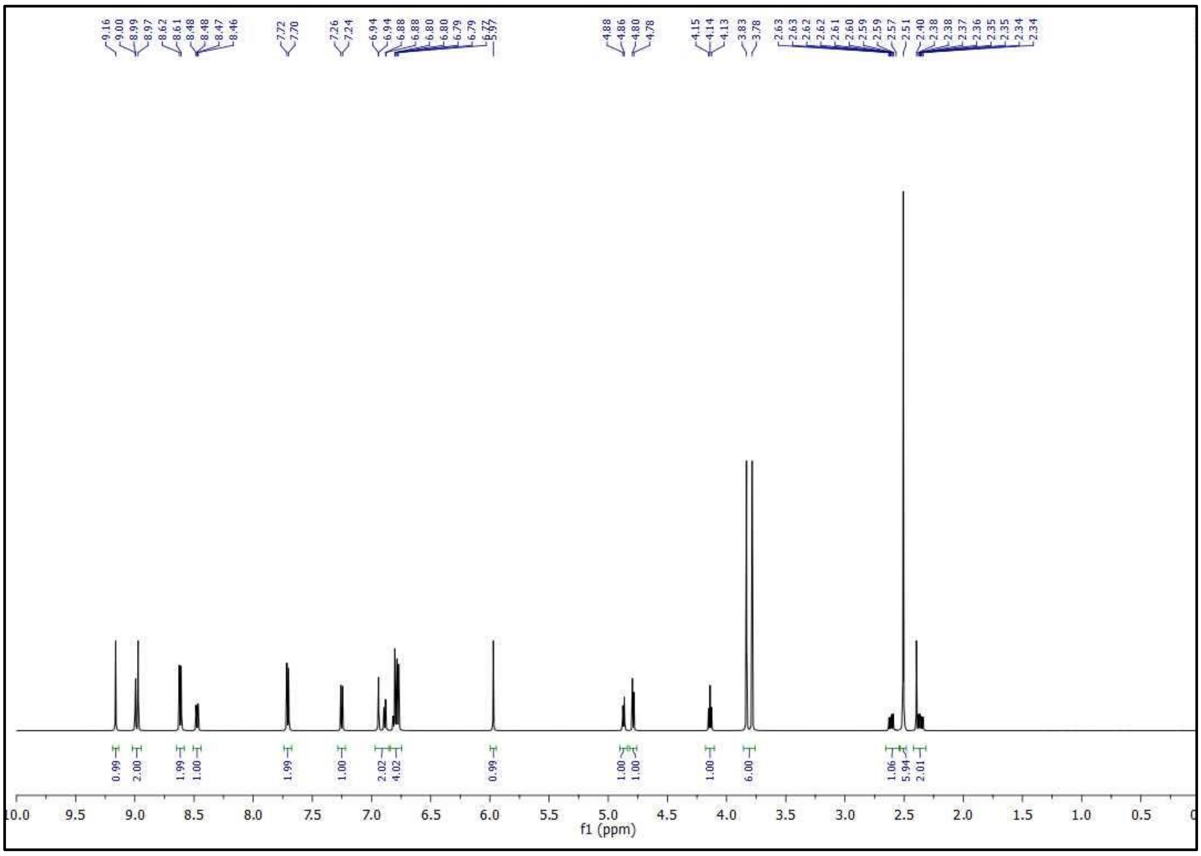

**Figure S8.** $^{13}C$ NMR of 4,4'-(1-(2,4-dinitrophenyl)-8-hydroxy-8-(pyridin-4-yl)-2,3,5,6,7,8-hexahydro-1H-pyrazolo[1, 5-d][1,2,4]triazepine-2,5-diyl)bis(2-methoxyphenol) (4d)

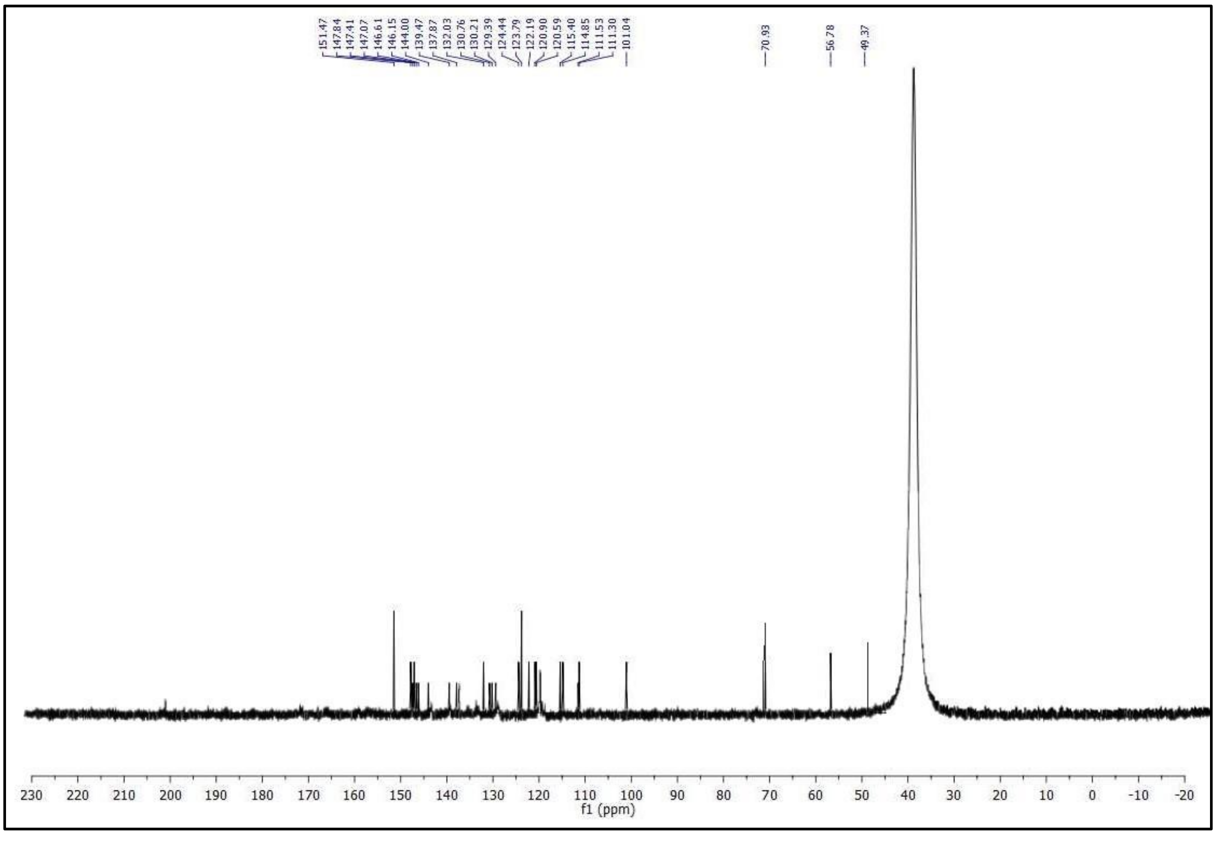

**Figure S9.** $^{1}H$ NMR of 2,2'-(1-(2,4-dinitrophenyl)-8-hydroxy-8-(pyridin-4-yl)-2,3,5,6,7,8-hexahydro-1H-pyrazolo[1 ,5-d][1,2,4]triazepine-2,5-diyl)diphenol (4e)

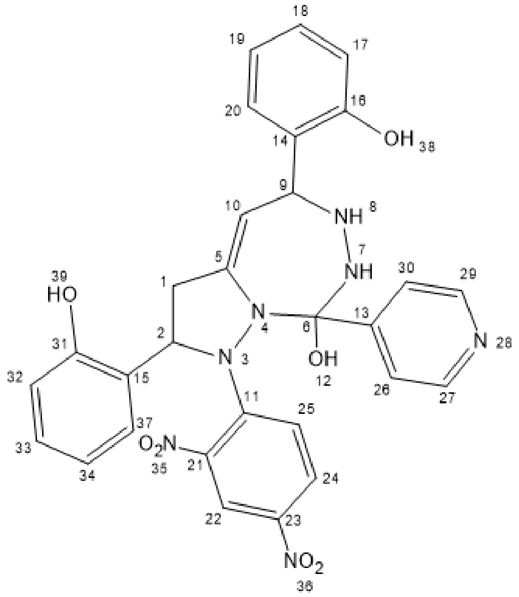


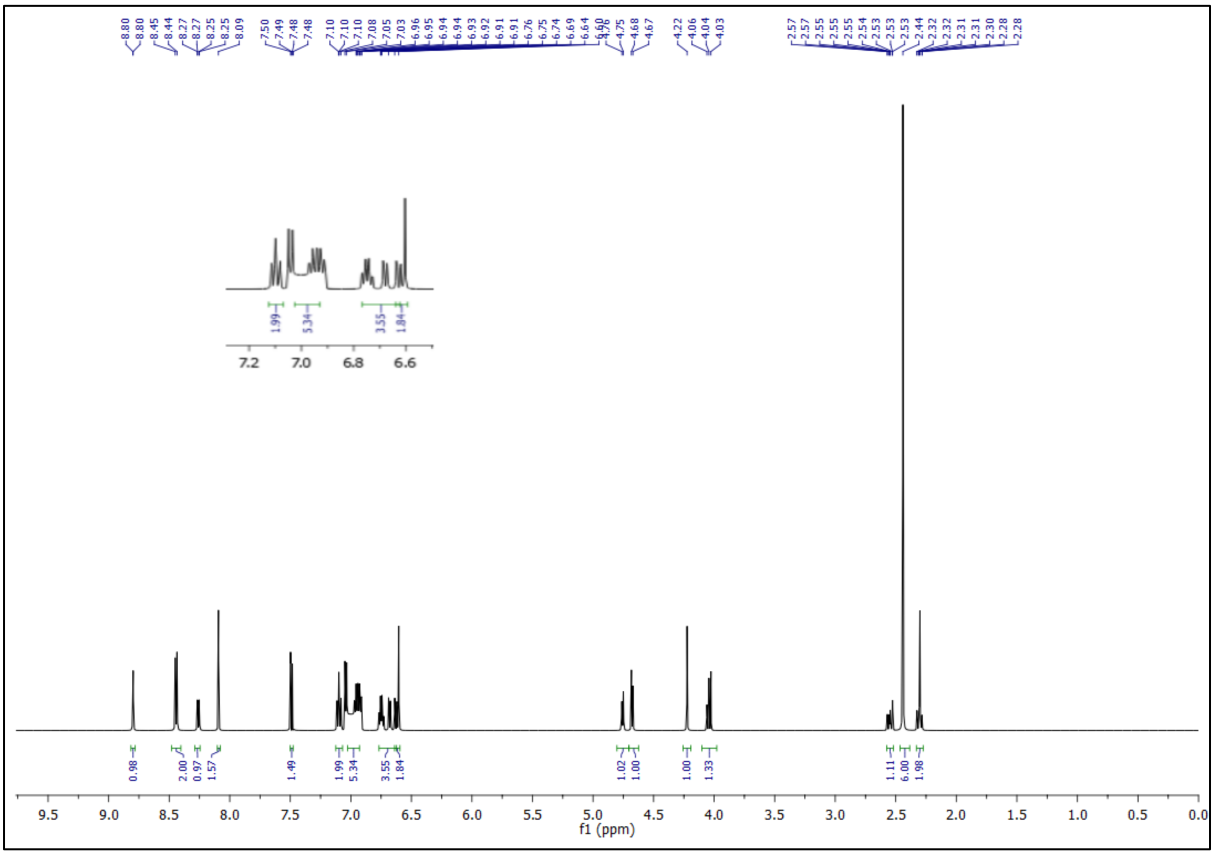


**Figure S10.** $^{13}C$ NMR of 2,2'-(1-(2,4-dinitrophenyl)-8-hydroxy-8-(pyridin-4-yl)-2,3,5,6,7,8-hexahydro-1H-pyrazolo[1 ,5-d][1,2,4]triazepine-2,5-diyl)diphenol (4e)

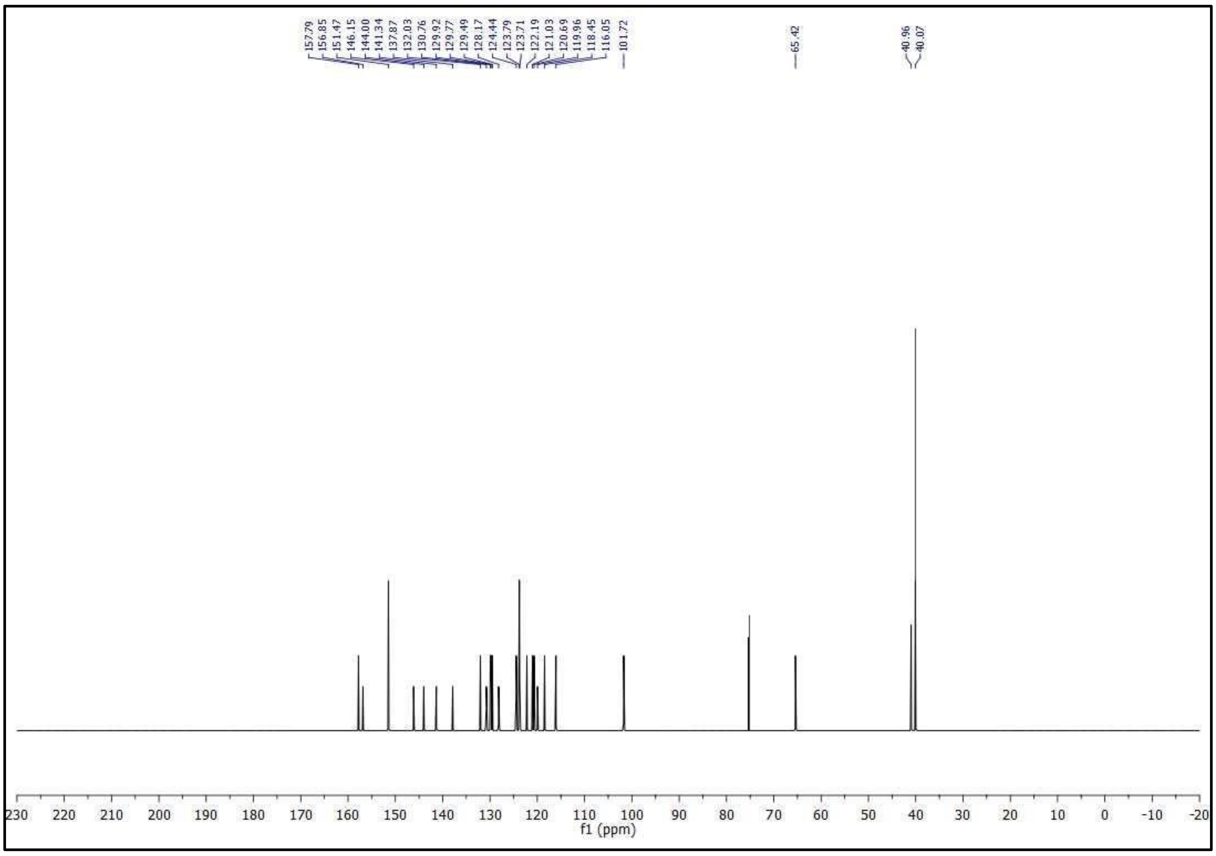


**Figure S11.** $^1$H NMR of 2,5-bis(4-(dimethylamino)phenyl)-1-(2,4-dinitrophenyl)-8-(pyridin-4-yl)-2,3,5,6,7,8-hexahydro-1H-pyrazolo[1,5-d][1,2,4]triazepin-8-ol (4f)

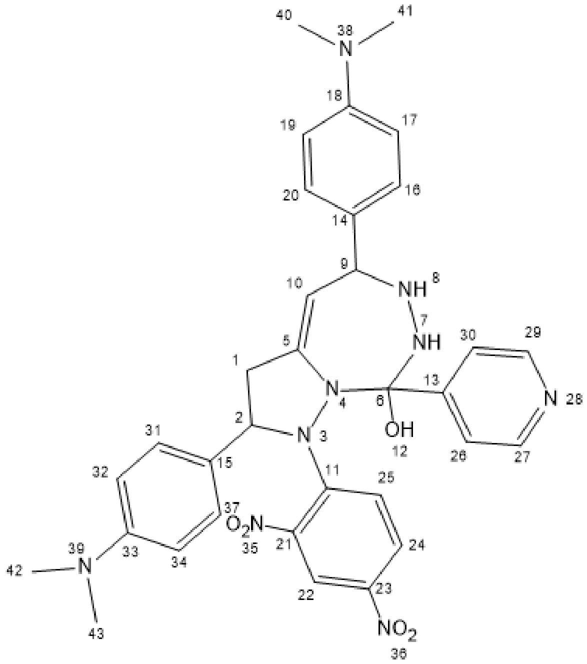

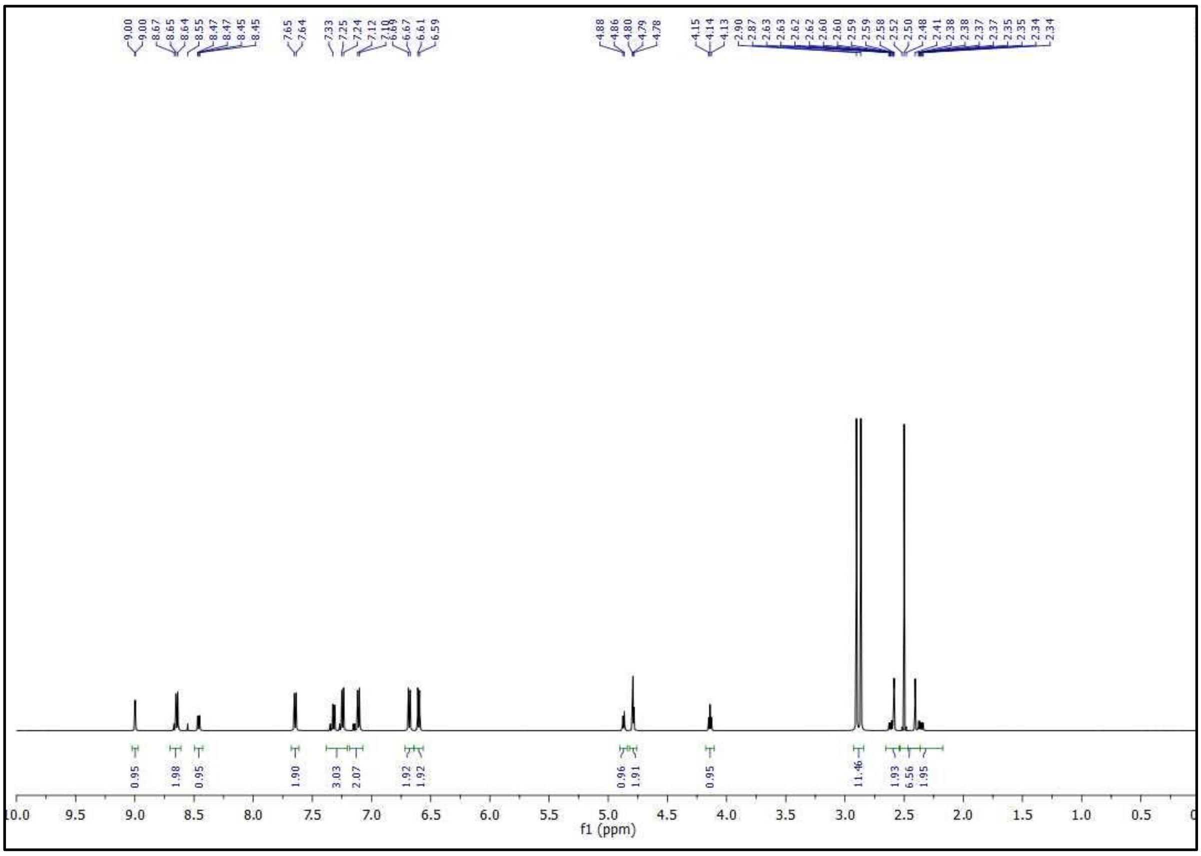


**Figure S12.** $^{13}$C NMR of 2,5-bis(4-(dimethylamino)phenyl)-1-(2,4-dinitrophenyl)-8-(pyridin-4-yl)-2,3,5,6,7,8-hexahydro-1H-pyrazolo[1,5-d][1,2,4]triazepin-8-ol (4f)

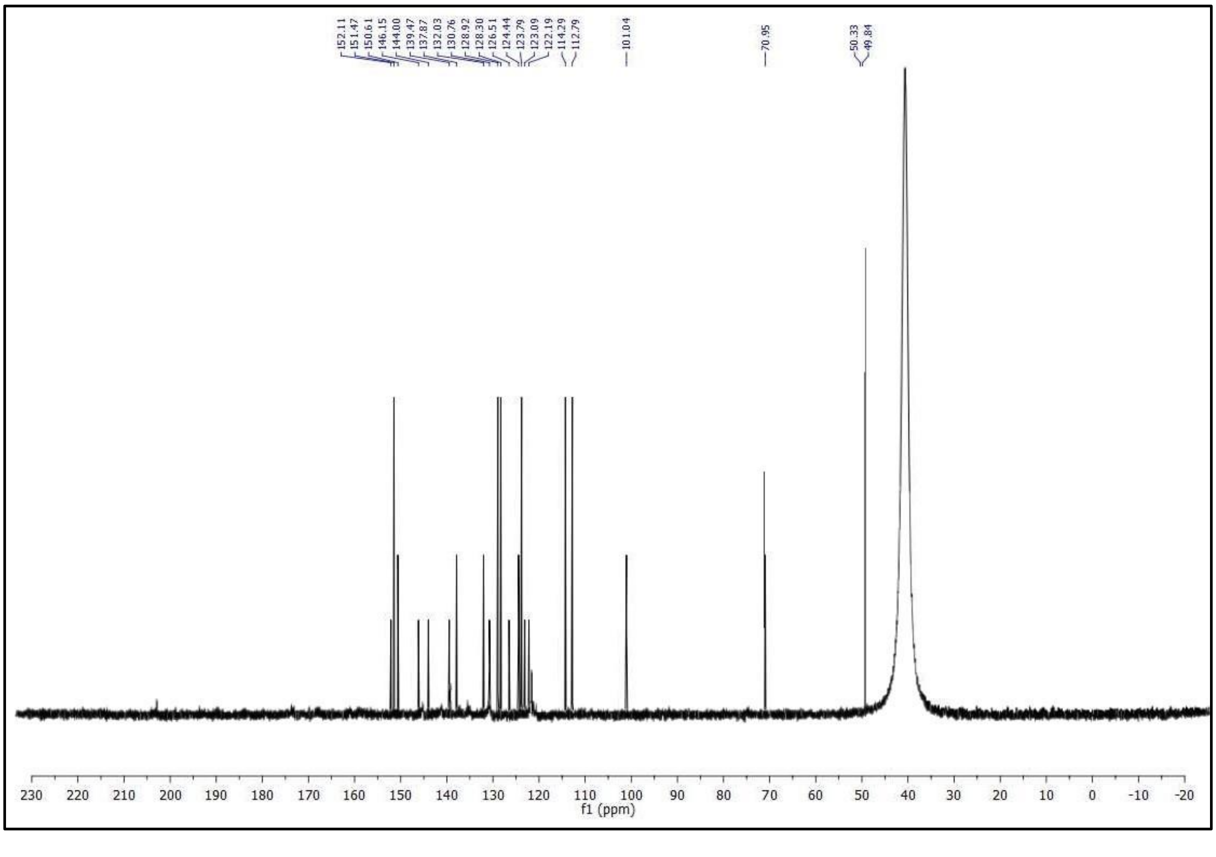

**Figure S13.** $^{1}$H NMR of2,5-bis(4-chlorophenyl)-1-(2,4-dinitrophenyl)-8-(pyridin-4-yl) 2,3,5,6,7,8-hexahydro-1H-pyrazolo[1,5-d][1,2,4]triazepin-8-ol (4g)

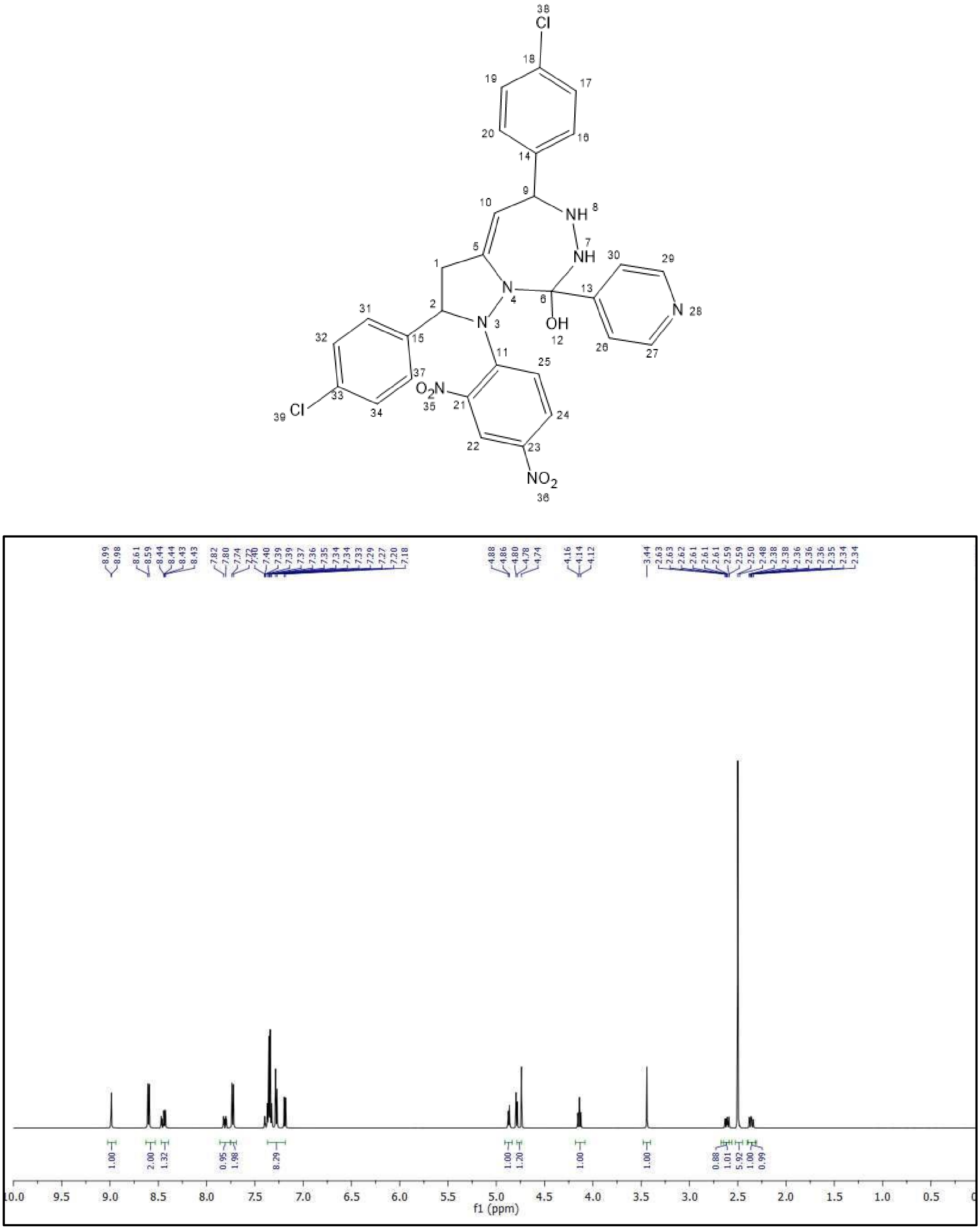


**Figure S14.** $^{13}$C NMR of 2,5-bis(4-chlorophenyl)-1-(2,4-dinitrophenyl)-8-(pyridin-4-yl)-2,3,5,6,7,8-hexahydro-1H-pyrazolo[1,5-d][1,2,4]triazepin-8-ol (4g)

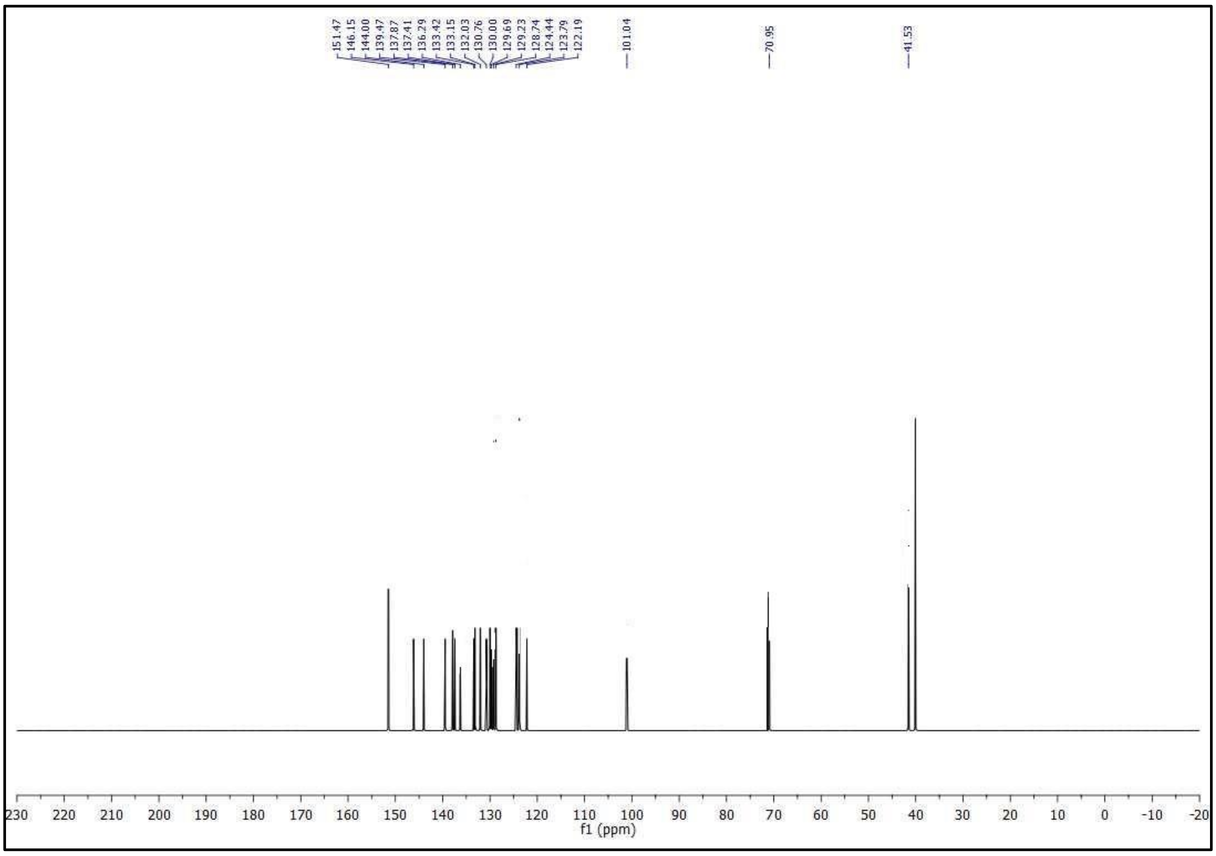


**Figure S15.** $^{1}$H NMR of 8-hydroxy-2,5-diphenyl-8-(pyridin-4-yl)-2,3,5,6,7,8-hexahydro-1H-pyrazolo[1,5-d][1,2,4] triazepine-1-carboxamide (4h)

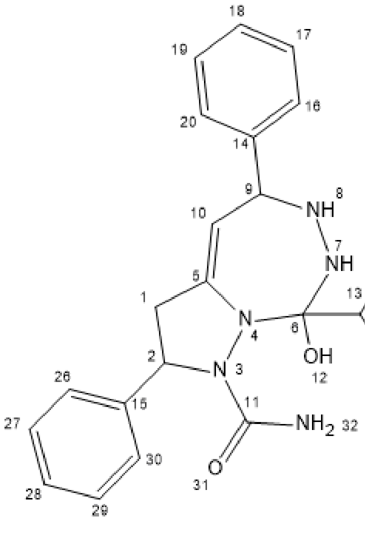

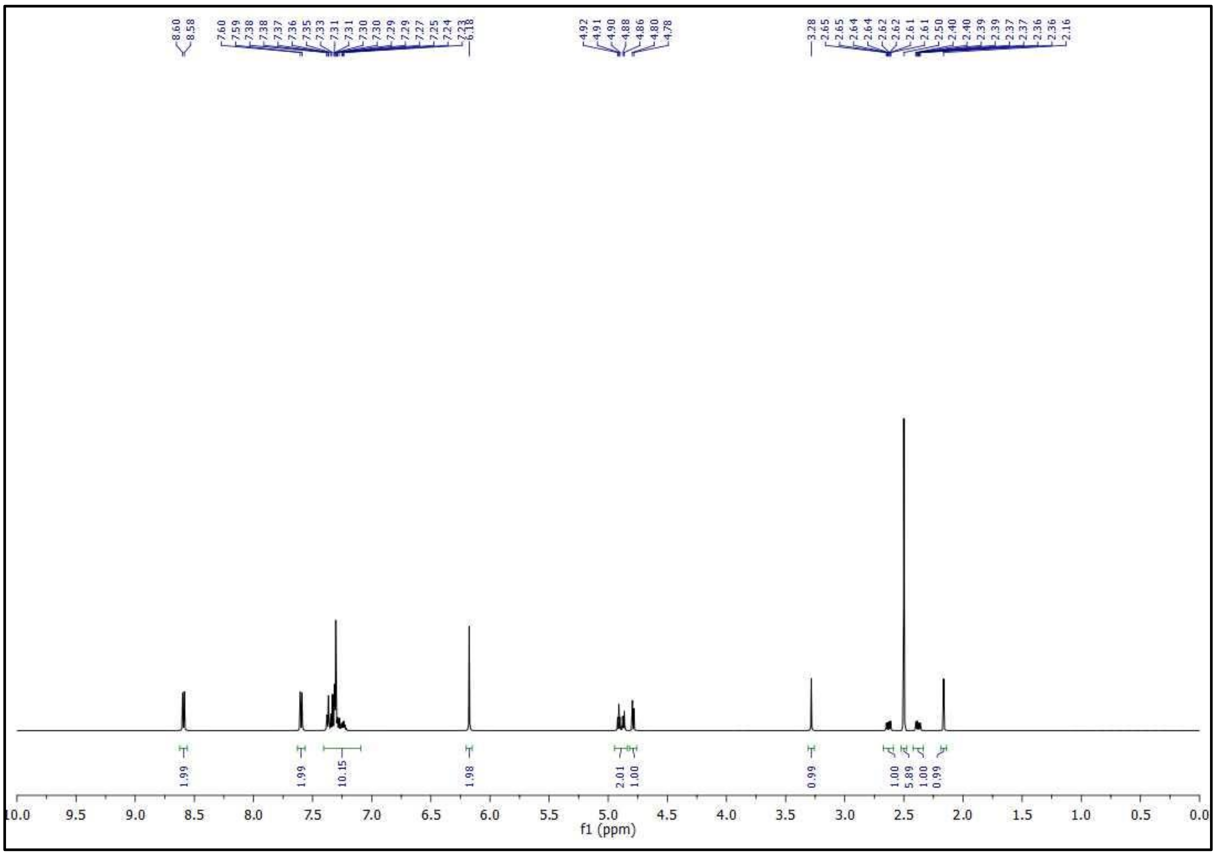


**Figure S16.** $^{13}$C NMR of 8-hydroxy-2,5-diphenyl-8-(pyridin-4-yl)-2,3,5,6,7,8-hexahydro-1H-pyrazolo[1,5-d][1,2,4] triazepine-1-carboxamide (4h)

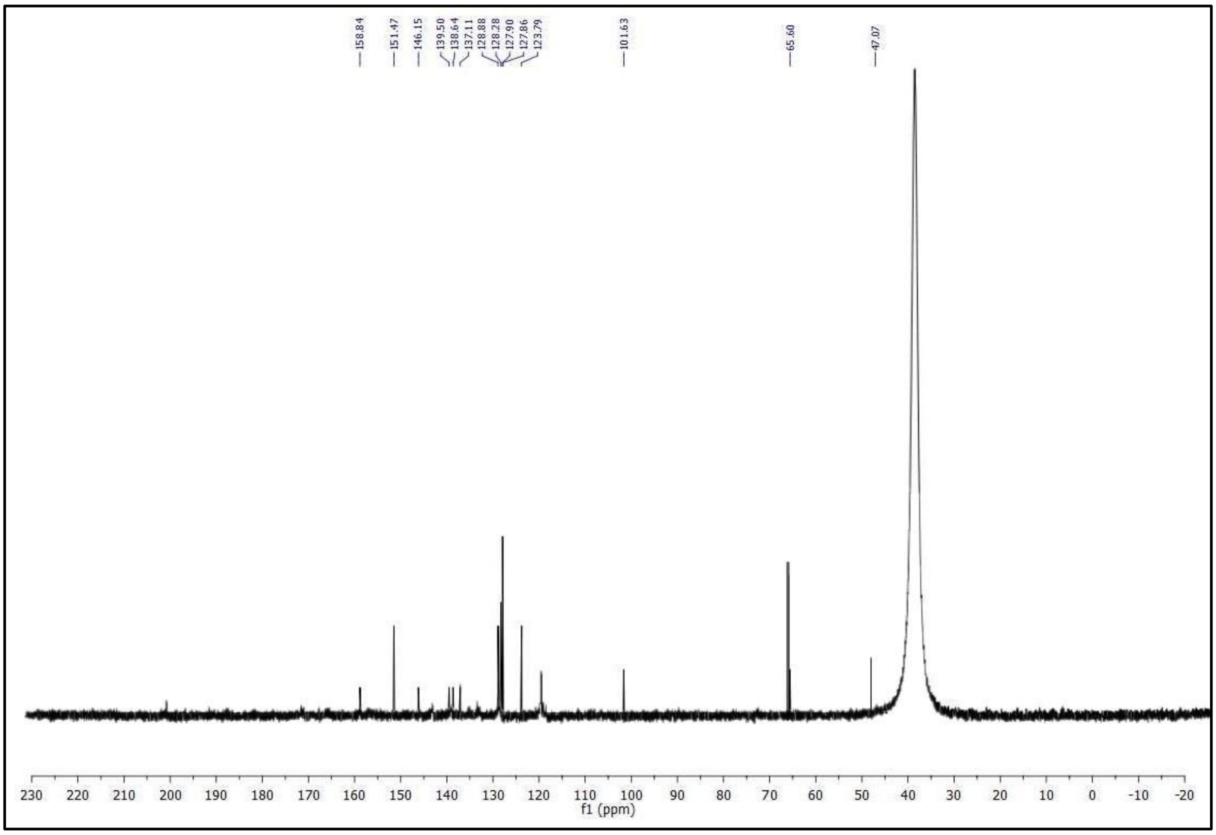

**Figure S17.** $^{1}$H NMR of 8-hydroxy-2,5-bis(4-hydroxyphenyl)-8-(pyridin-4-yl)-2,3,5,6,7,8-hexahydro-1H-pyrazolo[1,5-d][1,2,4]triazepine-1-carboxamide (4i)

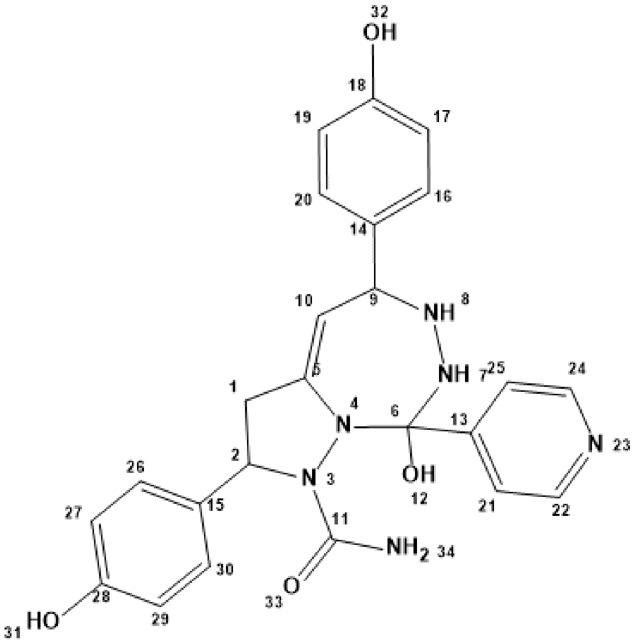


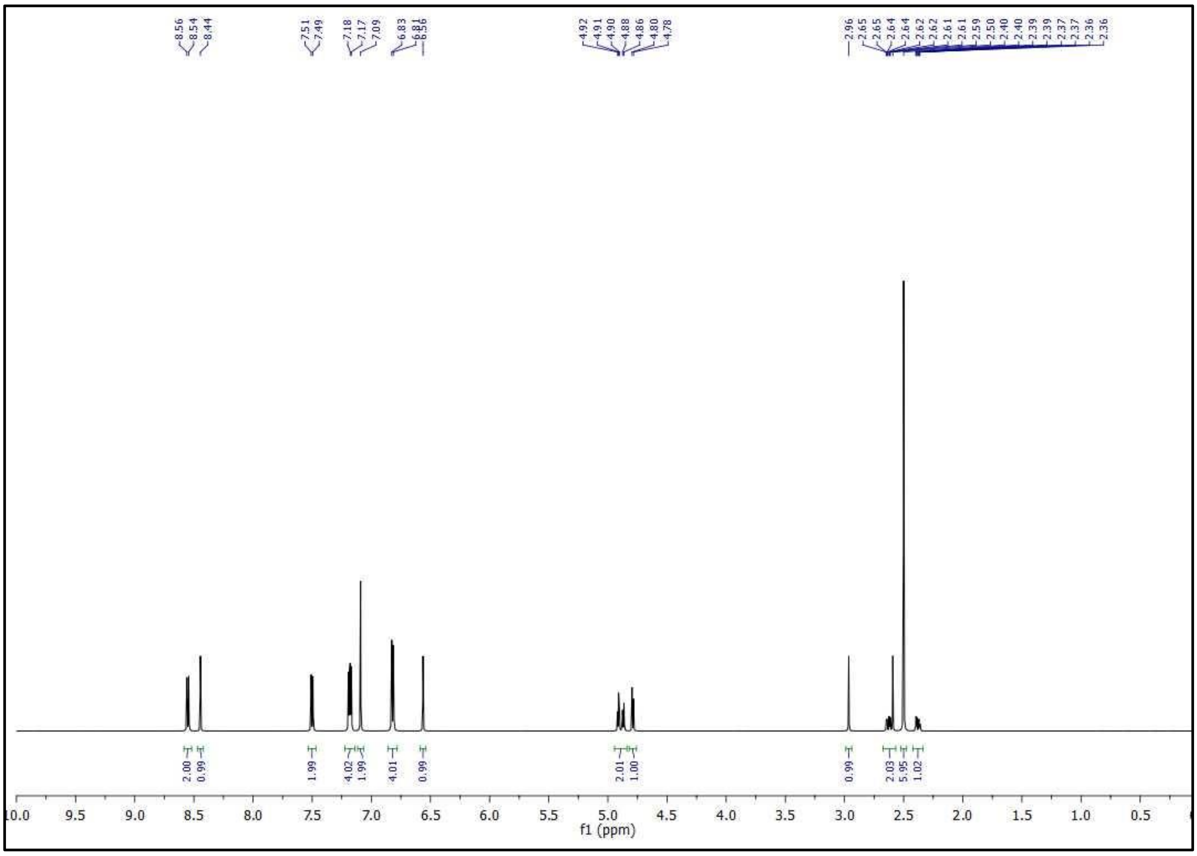


**Figure S18.** $^{13}$C NMR of 8-hydroxy-2,5-bis(4-hydroxyphenyl)-8-(pyridin-4-yl)-2,3,5,6,7,8-hexahydro-1H-pyrazolo[1,5-d][1,2,4]triazepine-1-carboxamide (4i)

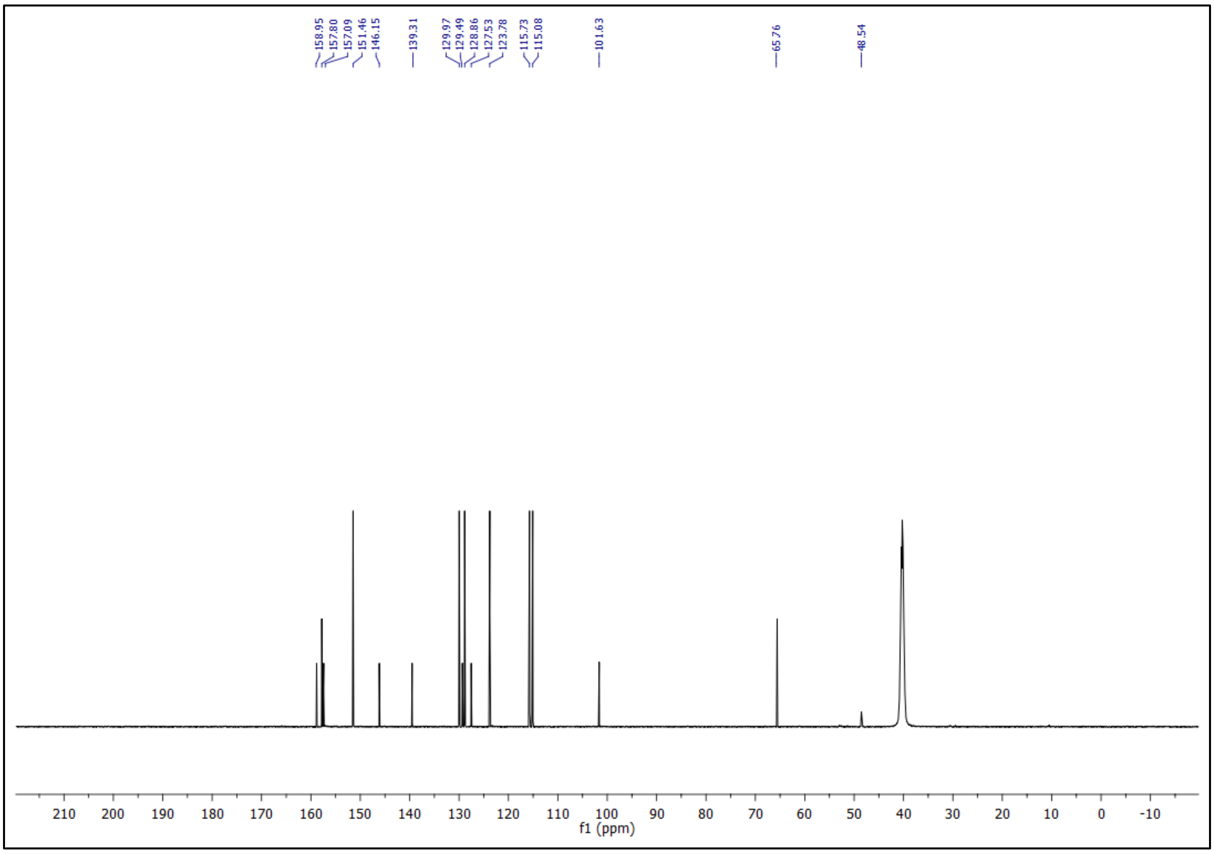


**Figure S19.** $^{1}$H NMR of 8-hydroxy-2,5-bis(4-nitrophenyl)-8-(pyridin-4-yl)-2,3,5,6,7,8-hexahydro-1H-pyrazolo[1,5-d][1,2,4]triazepine-1-carboxamide (4j)

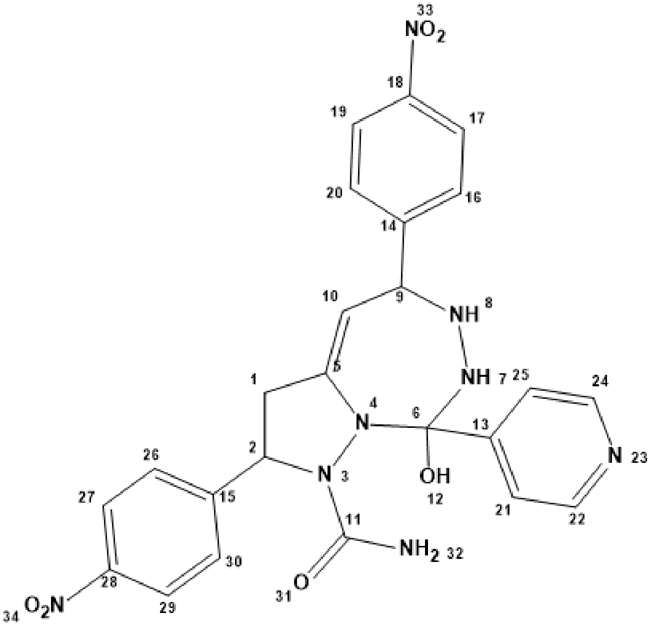

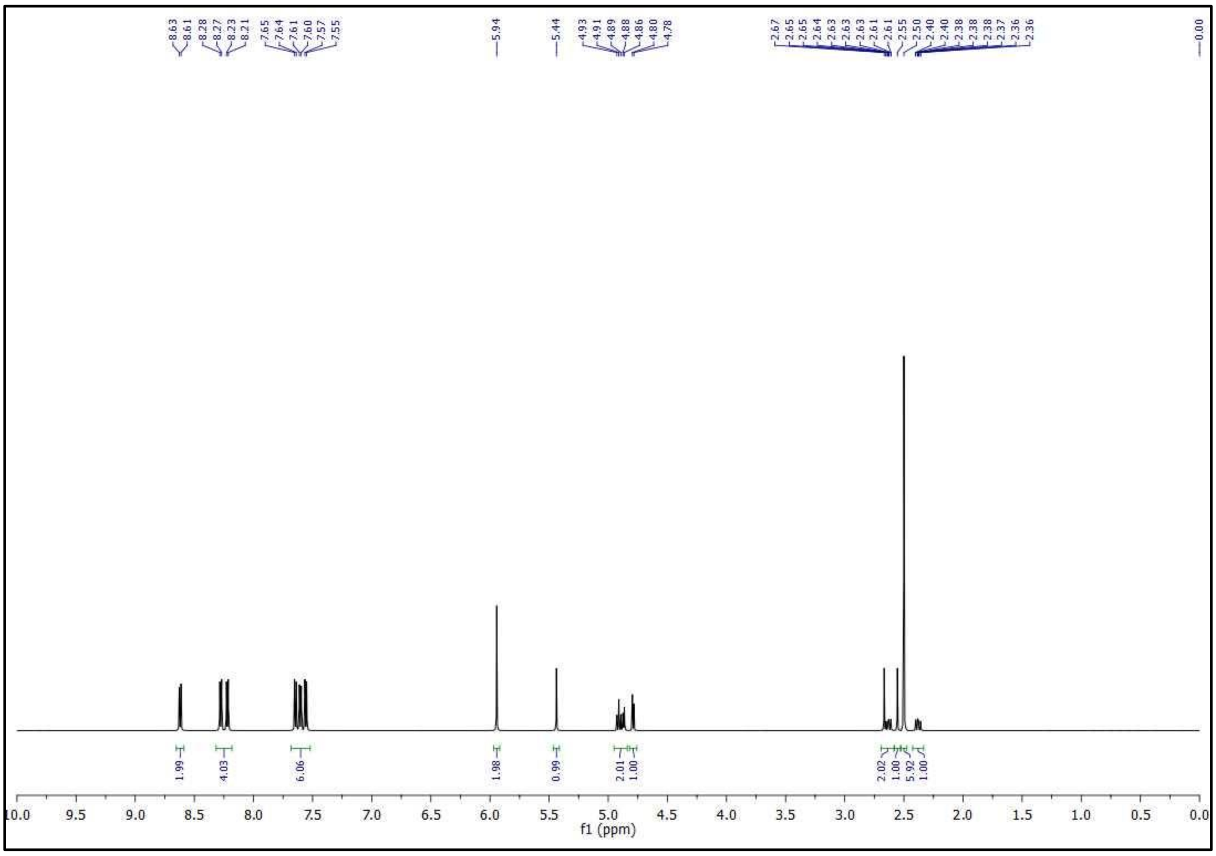


**Figure S20.** $^{13}$C NMR of 8-hydroxy-2,5-bis(4-nitrophenyl)-8-(pyridin-4-yl)-2,3,5,6,7,8-hexahydro-1H-pyrazolo[1,5-d][1,2,4]triazepine-1-carboxamide (4j)

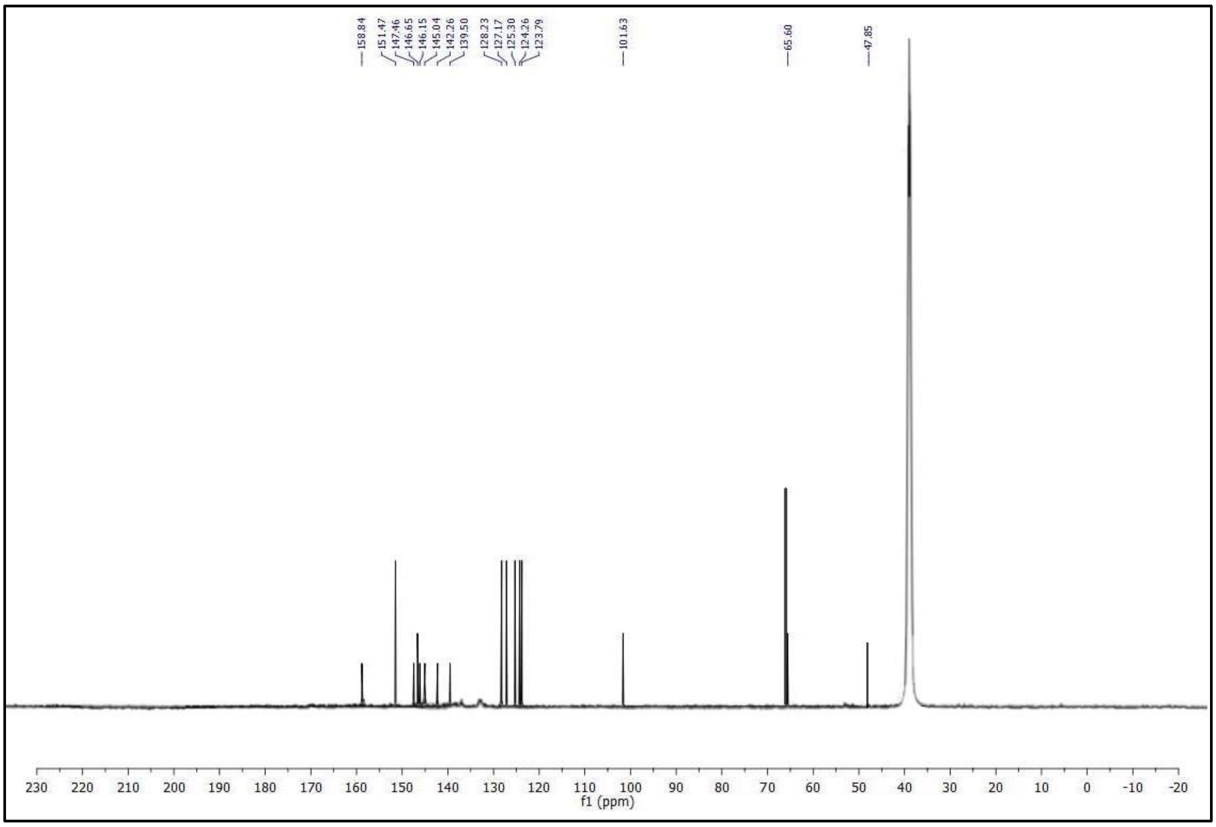

**Figure S21.** $^{1}H$ NMR of 8-hydroxy-2,5-bis(4-hydroxy-3-methoxyphenyl)-8-(pyridin-4-yl)-2,3,5,6,7,8-hexahydro-1H-pyrazolo[1,5-d][1,2,4]triazepine-1-carboxamide (4k)

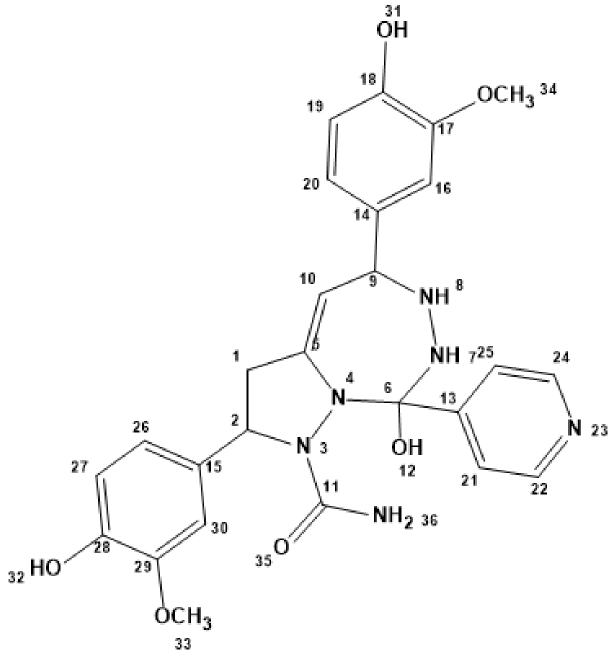


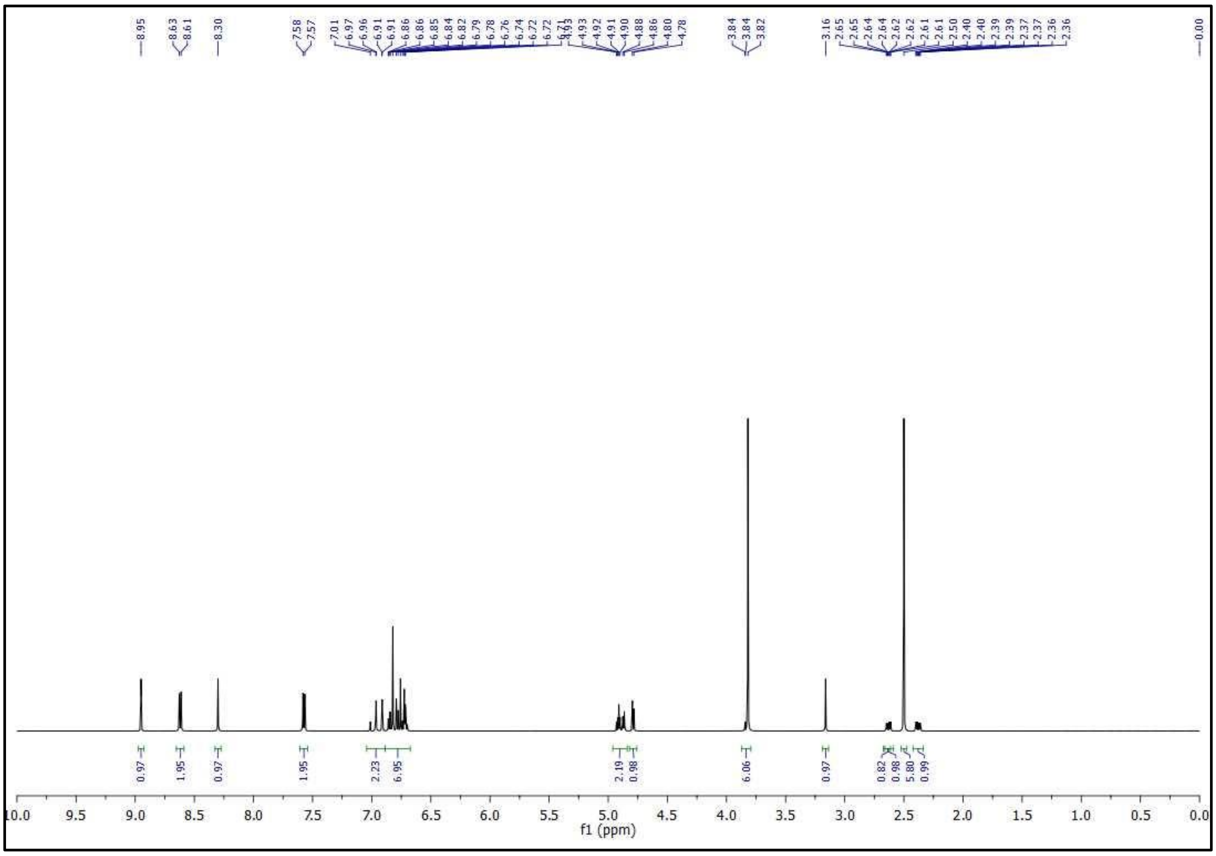


**Figure S22.** $^{13}C$ NMR of 8-hydroxy-2,5-bis(4-hydroxy-3-methoxyphenyl)-8-(pyridin-4-yl)-2,3,5,6,7,8-hexahydro-1H-pyrazolo[1,5-d][1,2,4]triazepine-1-carboxamide (4k)

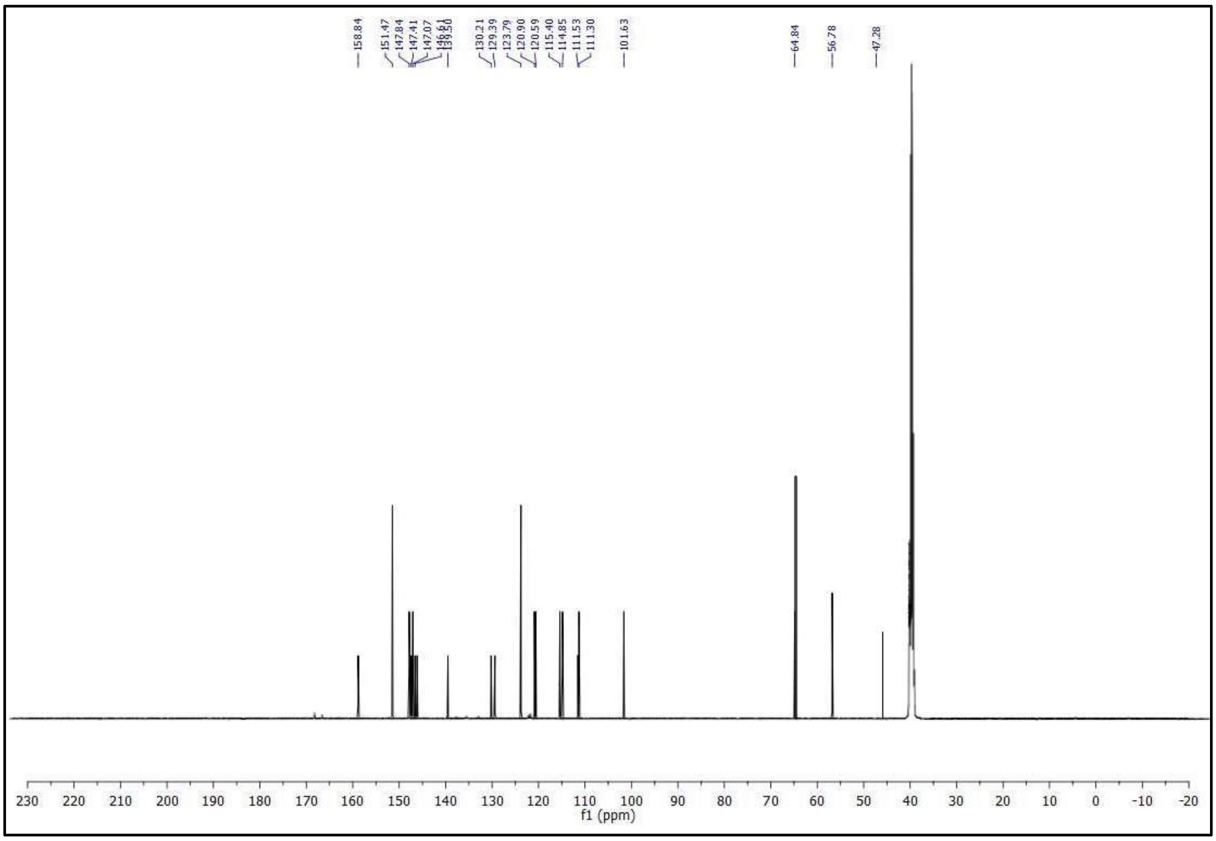


**Figure S23.** $^{1}$H NMR of 8-hydroxy-2,5-bis(2-hydroxyphenyl)-8-(pyridin-4-yl)-2,3,5,6,7,8-hexahydro-1H-pyrazolo[1,5-d][1,2,4]triazepine-1-carboxamide (4l)

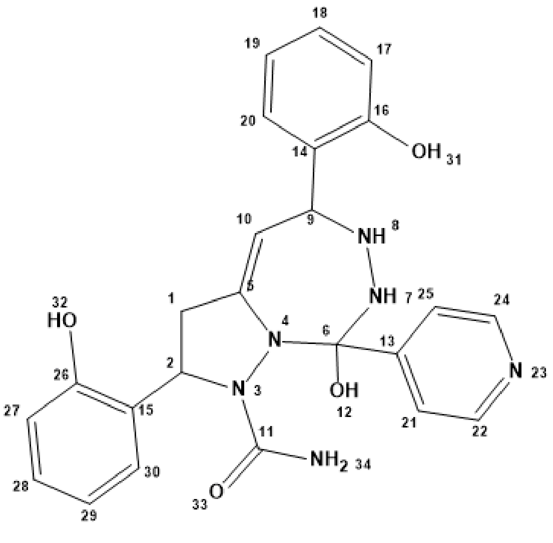

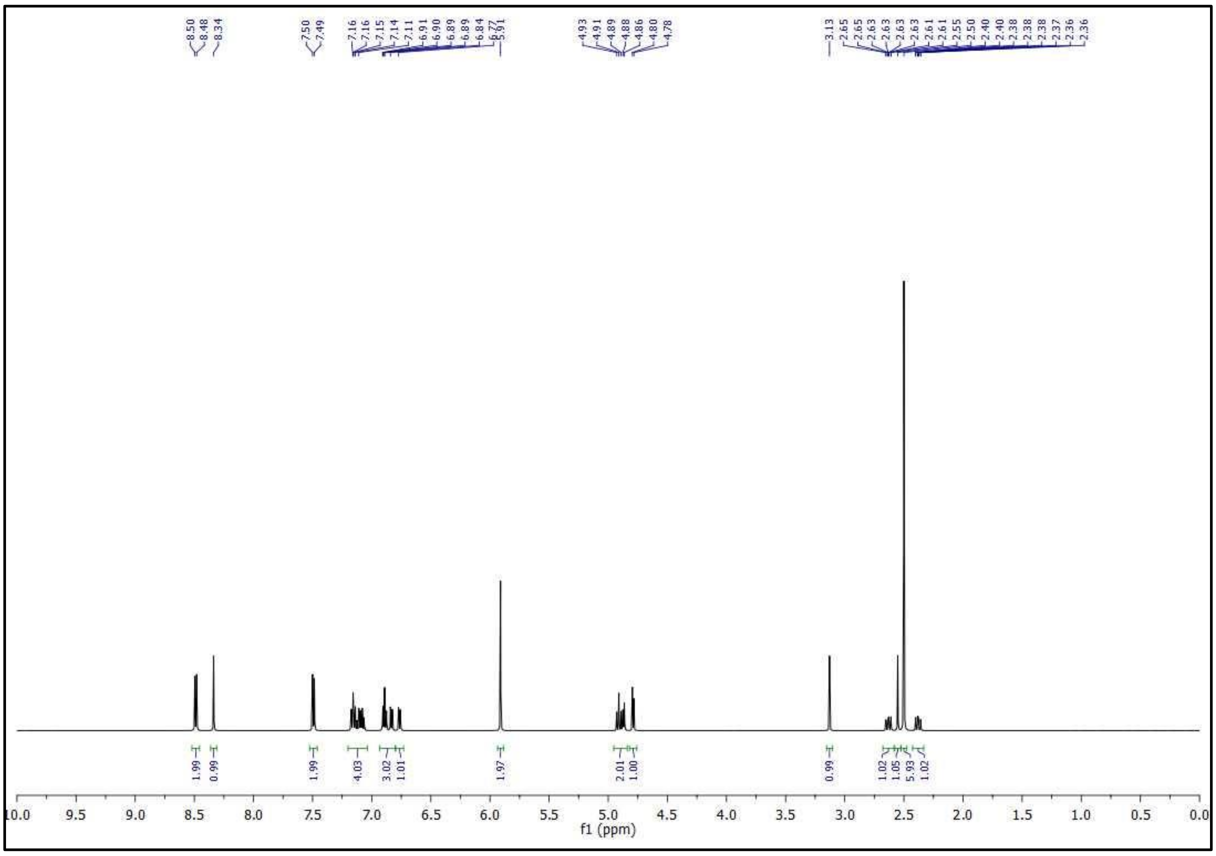

**Figure S24.** $^{13}C$ NMR of 8-hydroxy-2,5-bis(2-hydroxyphenyl)-8-(pyridin-4-yl)-2,3,5,6,7,8-hexahydro-1H-pyrazolo[1,5-d][1,2,4]triazepine-1-carboxamide (4l)

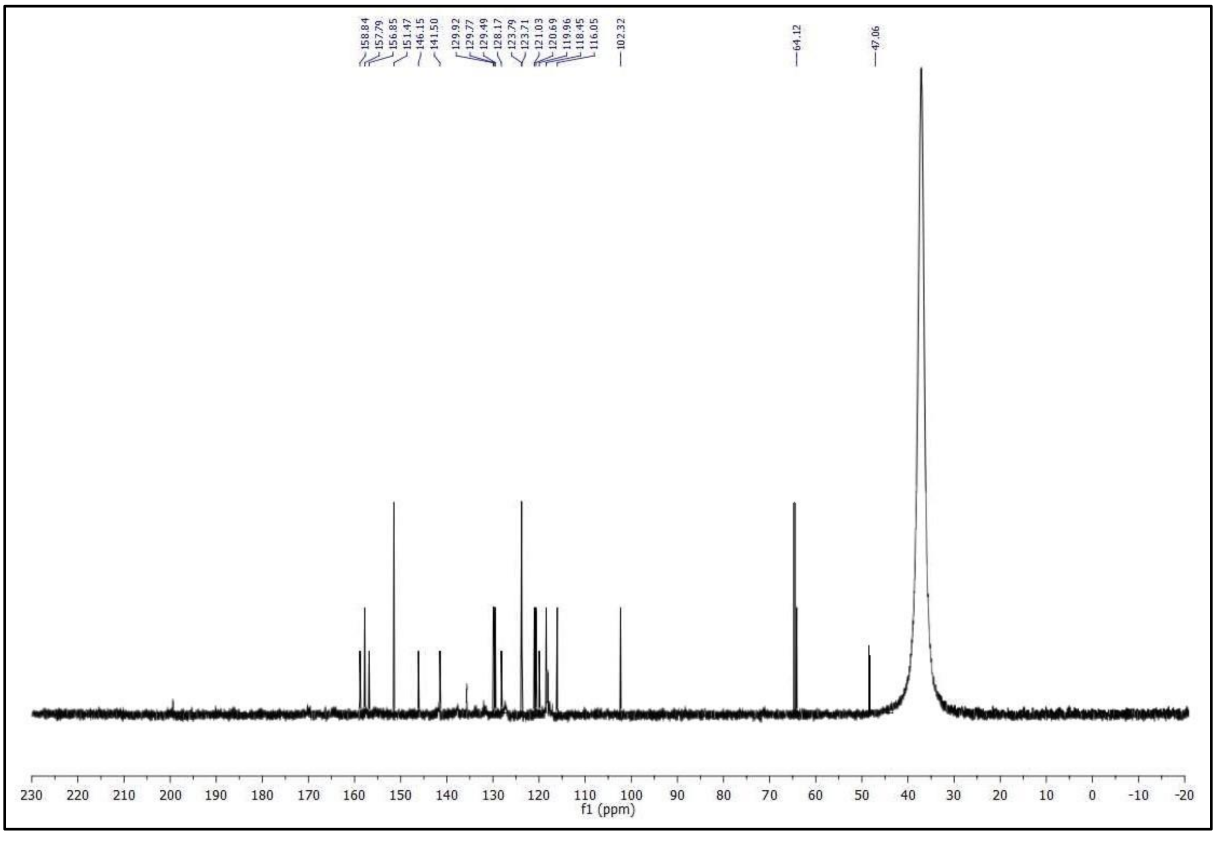

**Figure S25.** $^{1}H$ NMR of 2,5-bis(4-(dimethylamino)phenyl)-8-hydroxy-8-(pyridin-4-yl)-2,3,5,6,7,8-hexahydro-1H-pyrazolo[1,5-d][1,2,4]triazepine-1-carboxamidecarboxamide (4m)

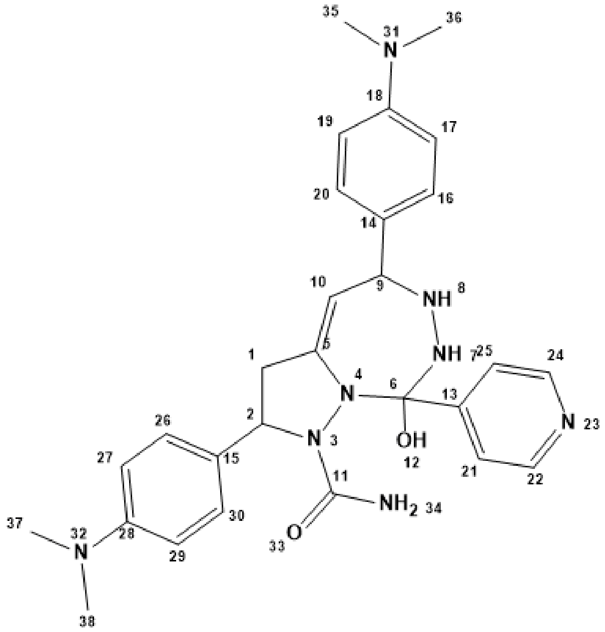

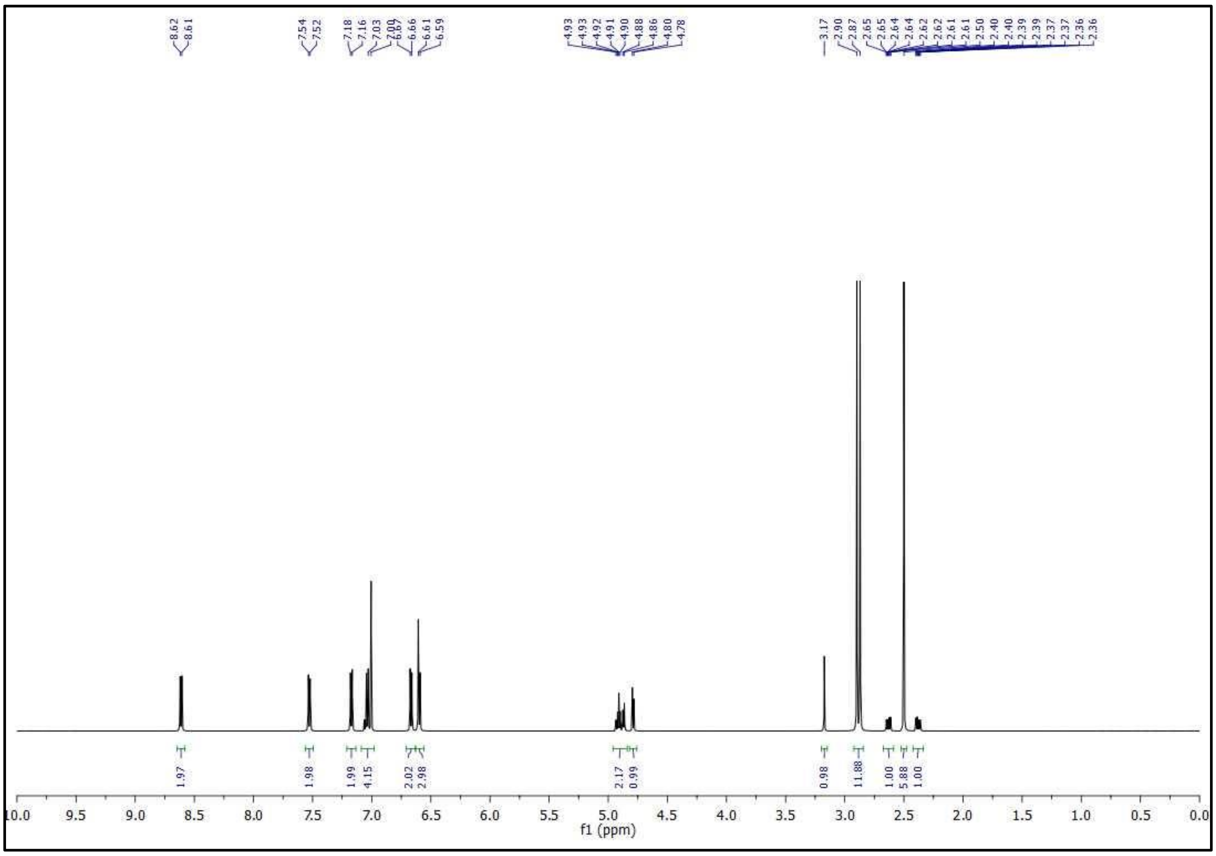


**Figure S26.** $^{13}C$ NMR of 2,5-bis(4-(dimethylamino)phenyl)-8-hydroxy-8-(pyridin-4-yl)-2,3,5,6,7,8-hexahydro-1H-pyrazolo[1,5-d][1,2,4]triazepine-1-carboxamide (4m)

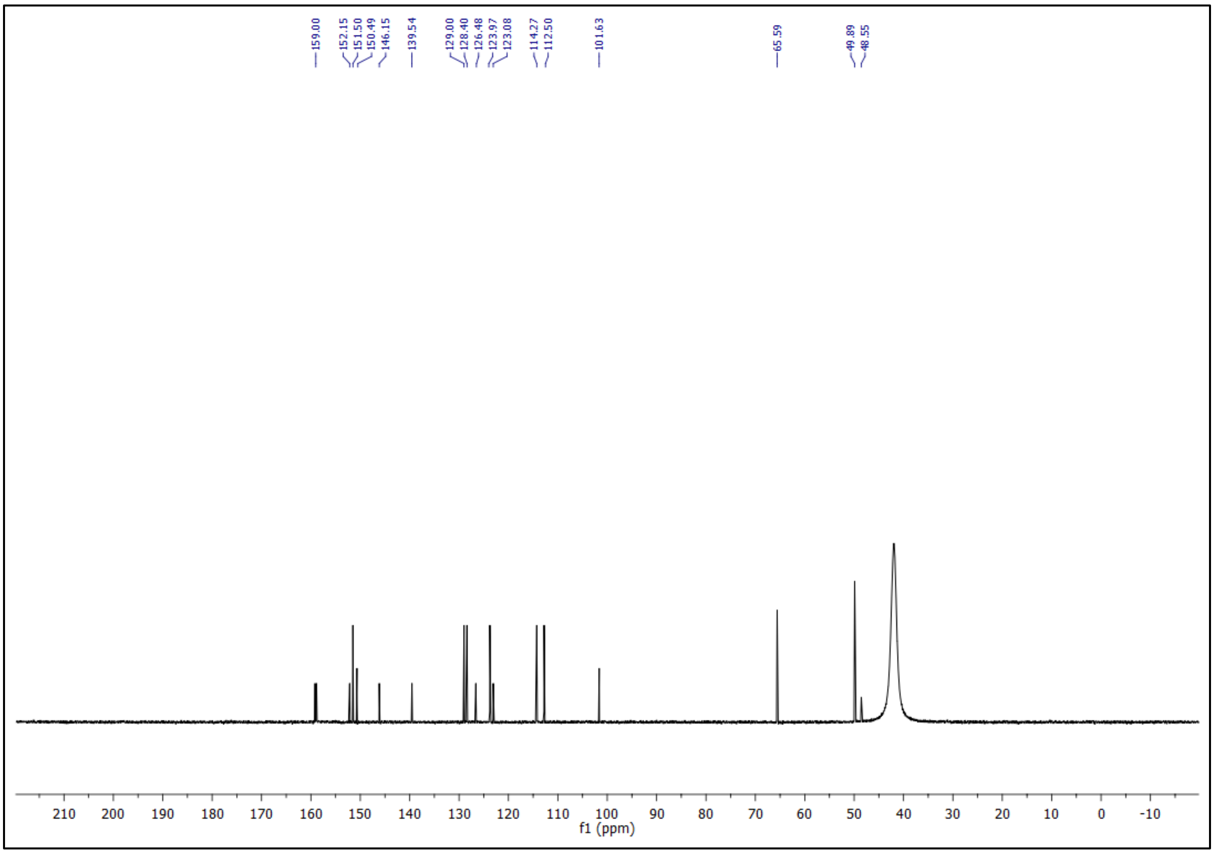


**Figure S27.** $^{1}$H NMR of 2,5-bis(4-chlorophenyl)-8-hydroxy-8-(pyridin-4-yl)-2,3,5,6,7,8-hexahydro-1H-pyrazolo[1,5-d][1,2,4]triazepine-1-carboxamide (4n)

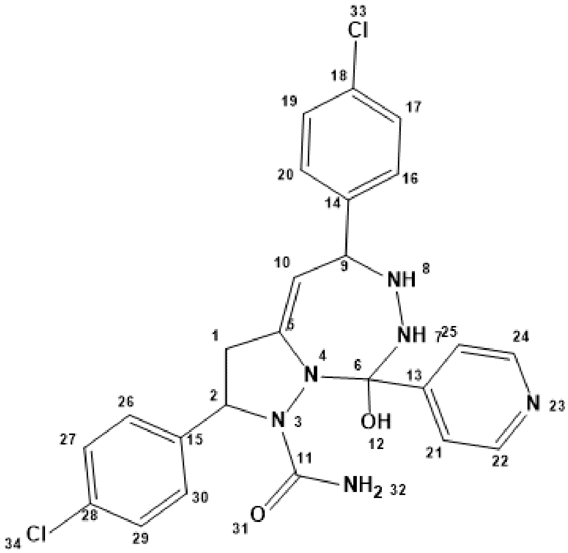

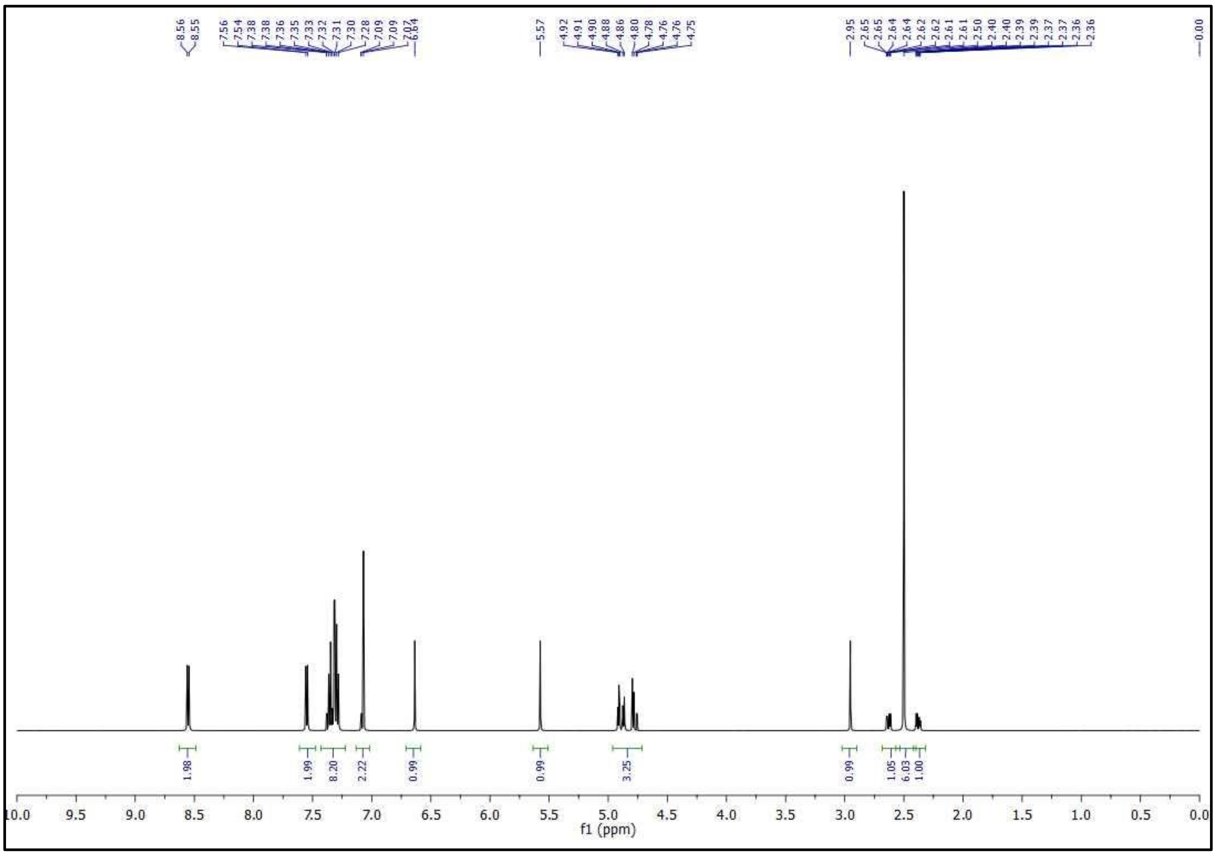


**Figure S28.** $^{13}$C NMR of 2,5-bis(4-chlorophenyl)-8-hydroxy-8-(pyridin-4-yl)-2,3,5,6,7,8-hexahydro-1H-pyrazolo[1,5-d][1,2,4]triazepine-1-carboxamide (4n)

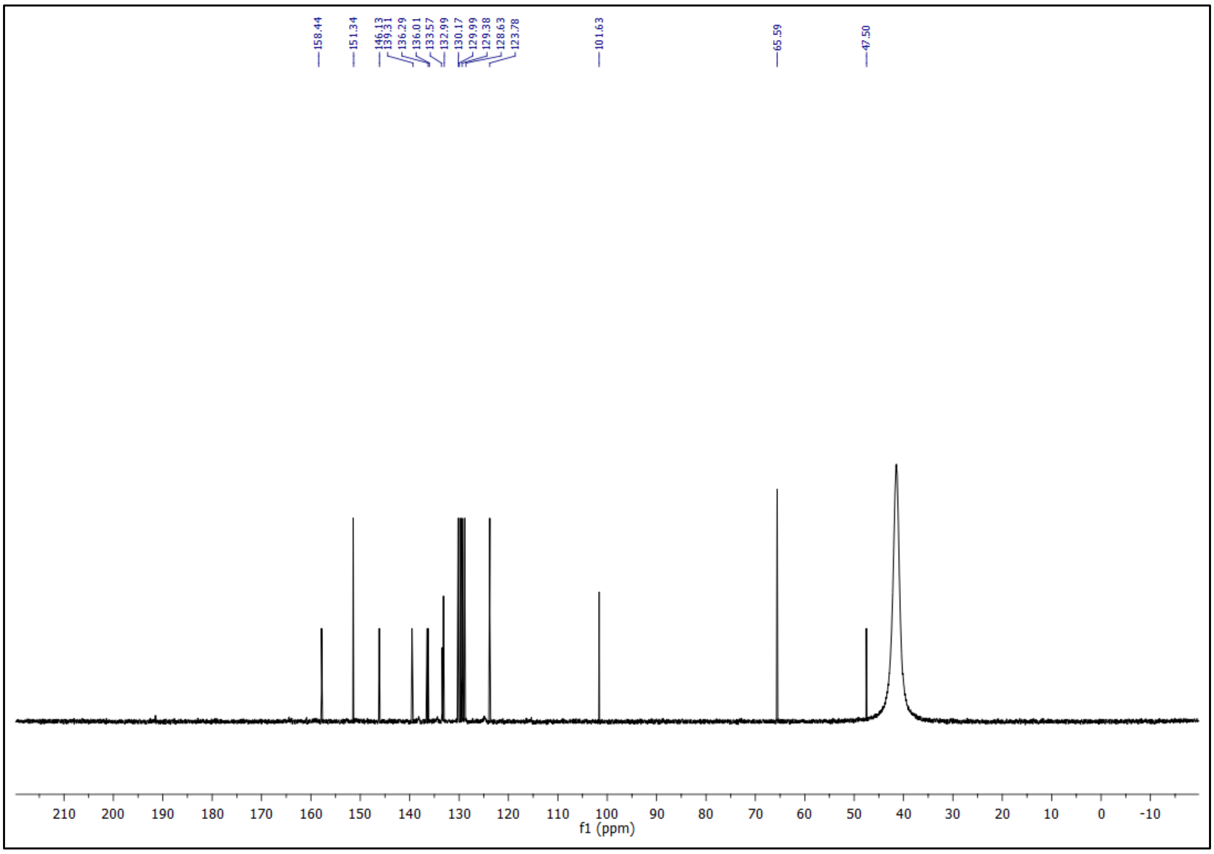

**Figure S29.** $^{1}$H NMR of 1,5-diphenylpenta-1,4-dien-3-one (1a)

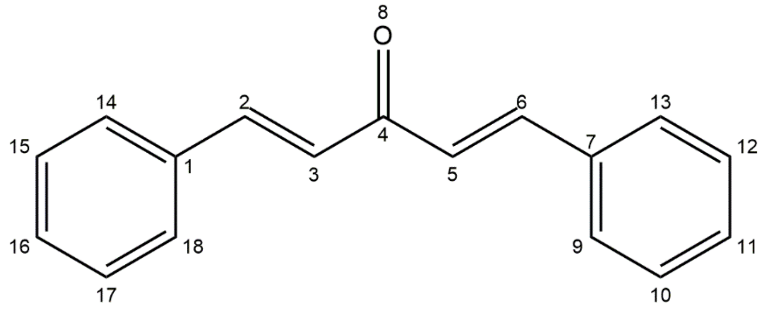


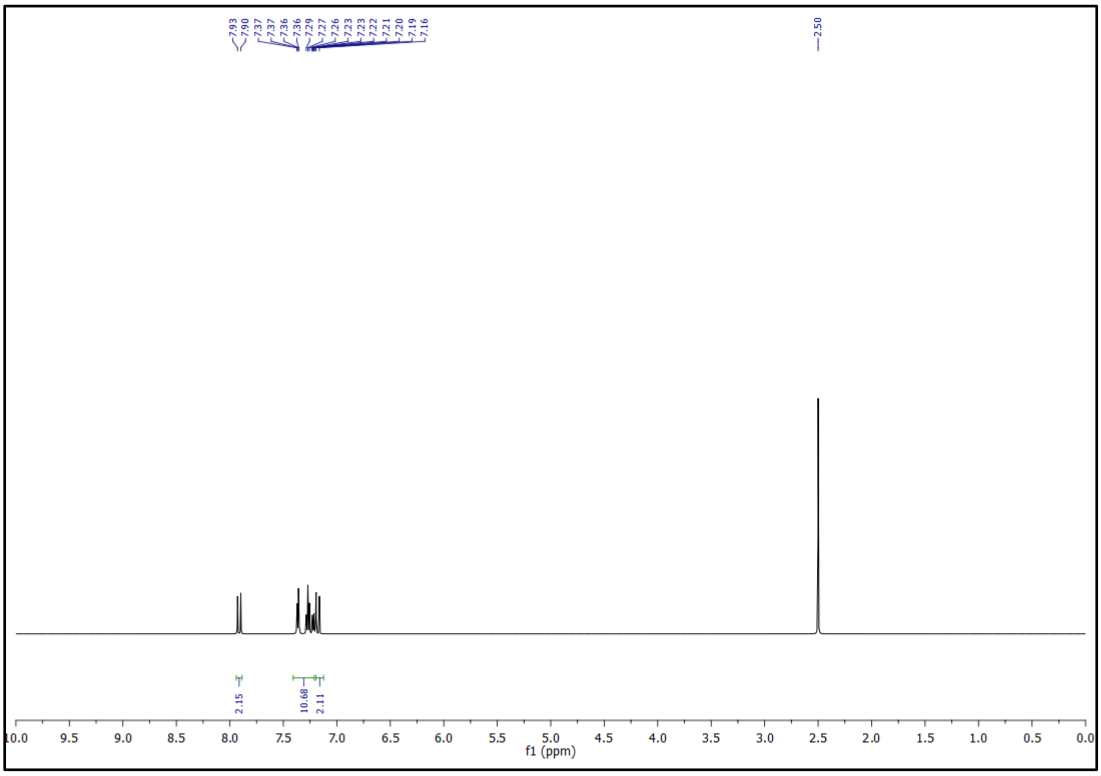

**Figure S30.** $^{13}C$ NMR of 1,5-diphenylpenta-1,4-dien-3-one (1a)

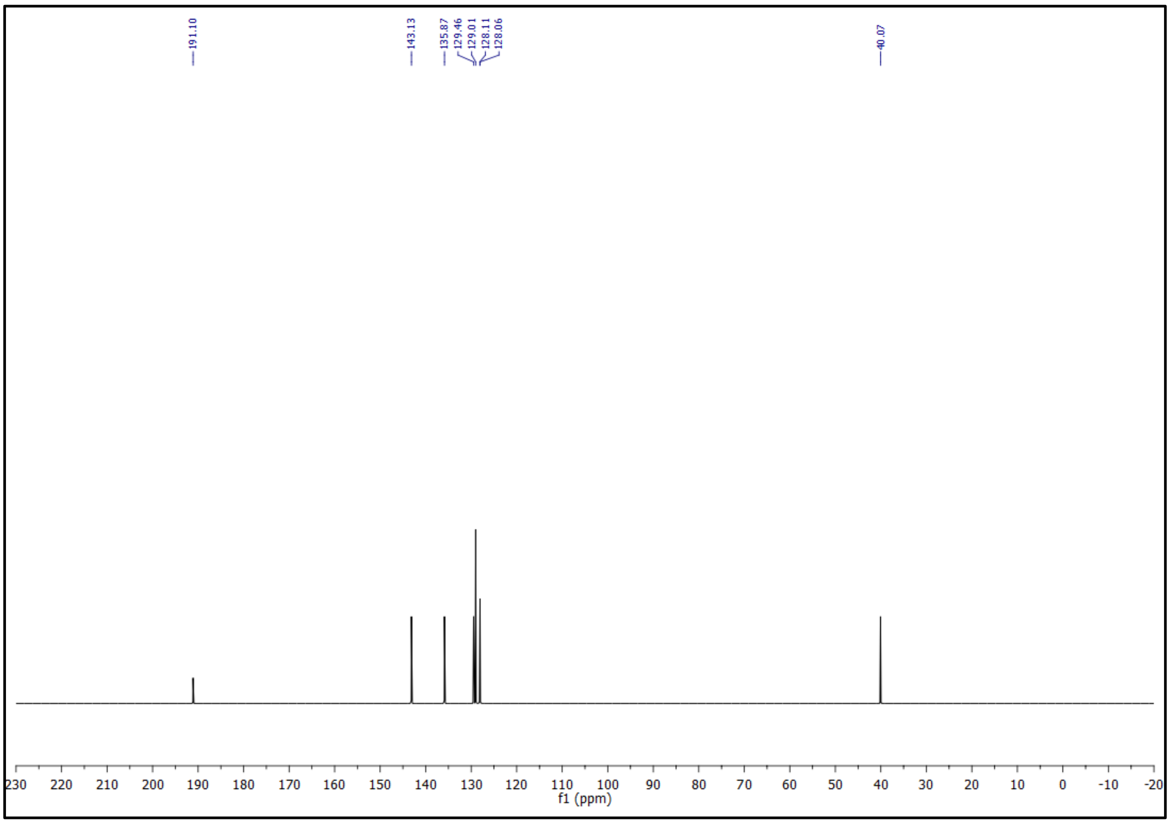

**Figure S31.** $^{1}$H NMR of 1,5-bis(4-hydroxyphenyl)penta-1,4-dien-3-one (1b)

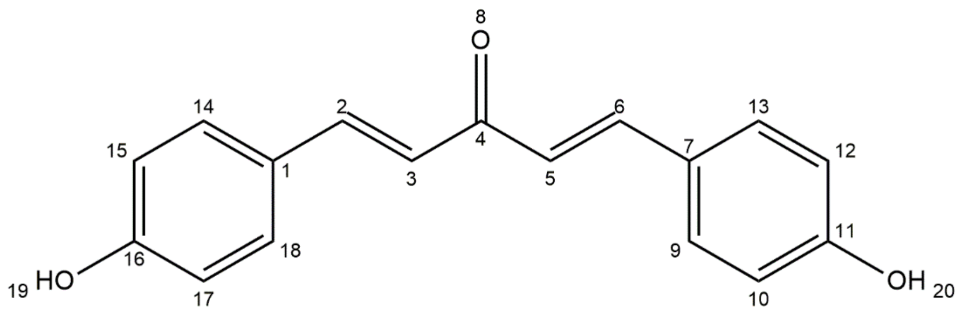


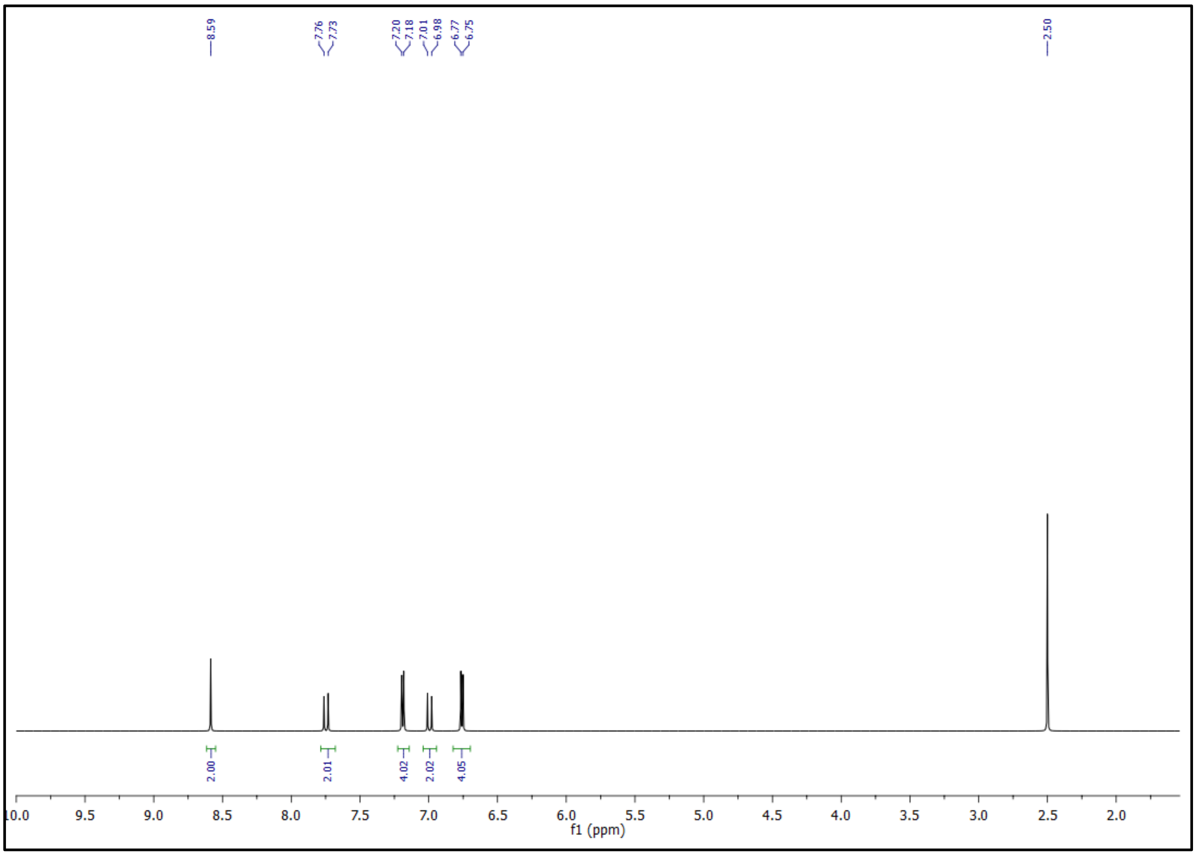

**Figure S32.** $^{13}C$ NMR of 1,5-bis(4-hydroxyphenyl)penta-1,4-dien-3-one (1b)

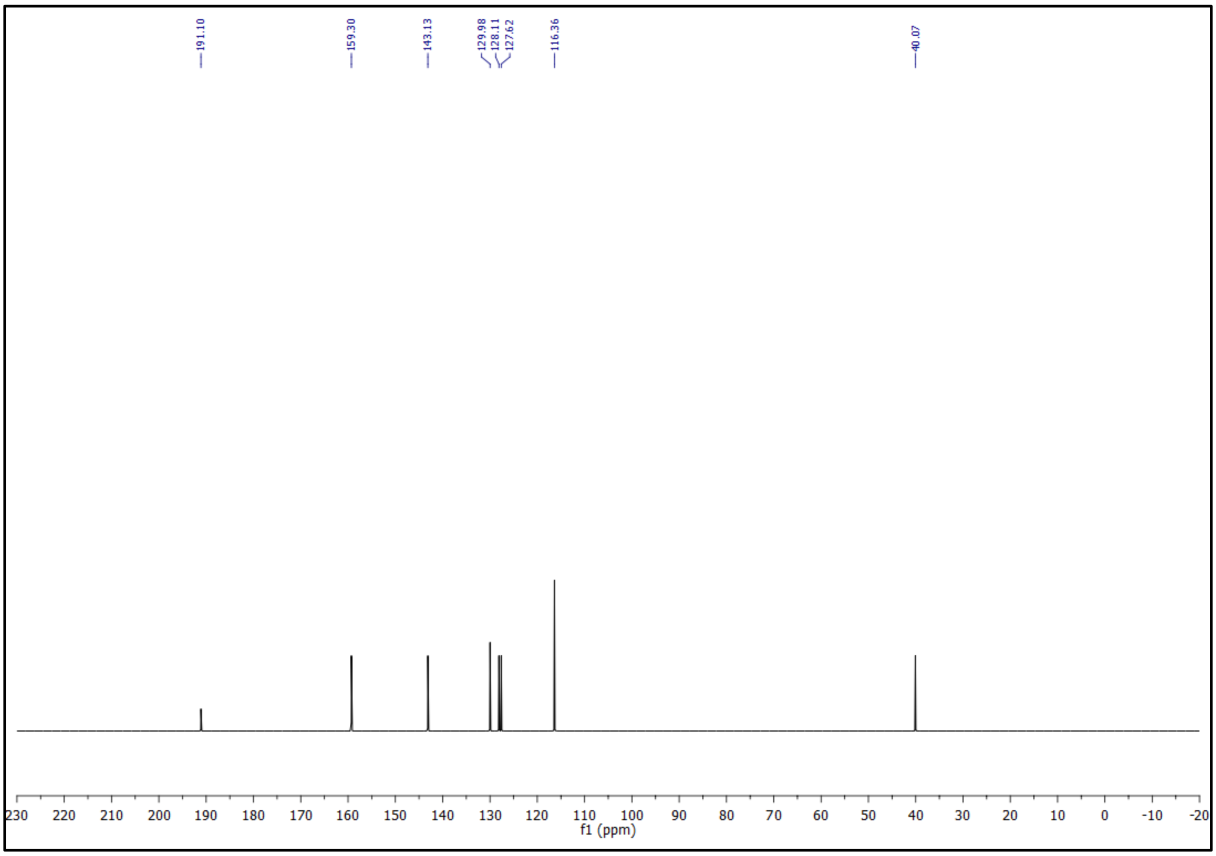

**Figure S33.** $^{1}$H NMR of 1,5-bis(4-nitrophenyl)penta-1,4-dien-3-one (1c)

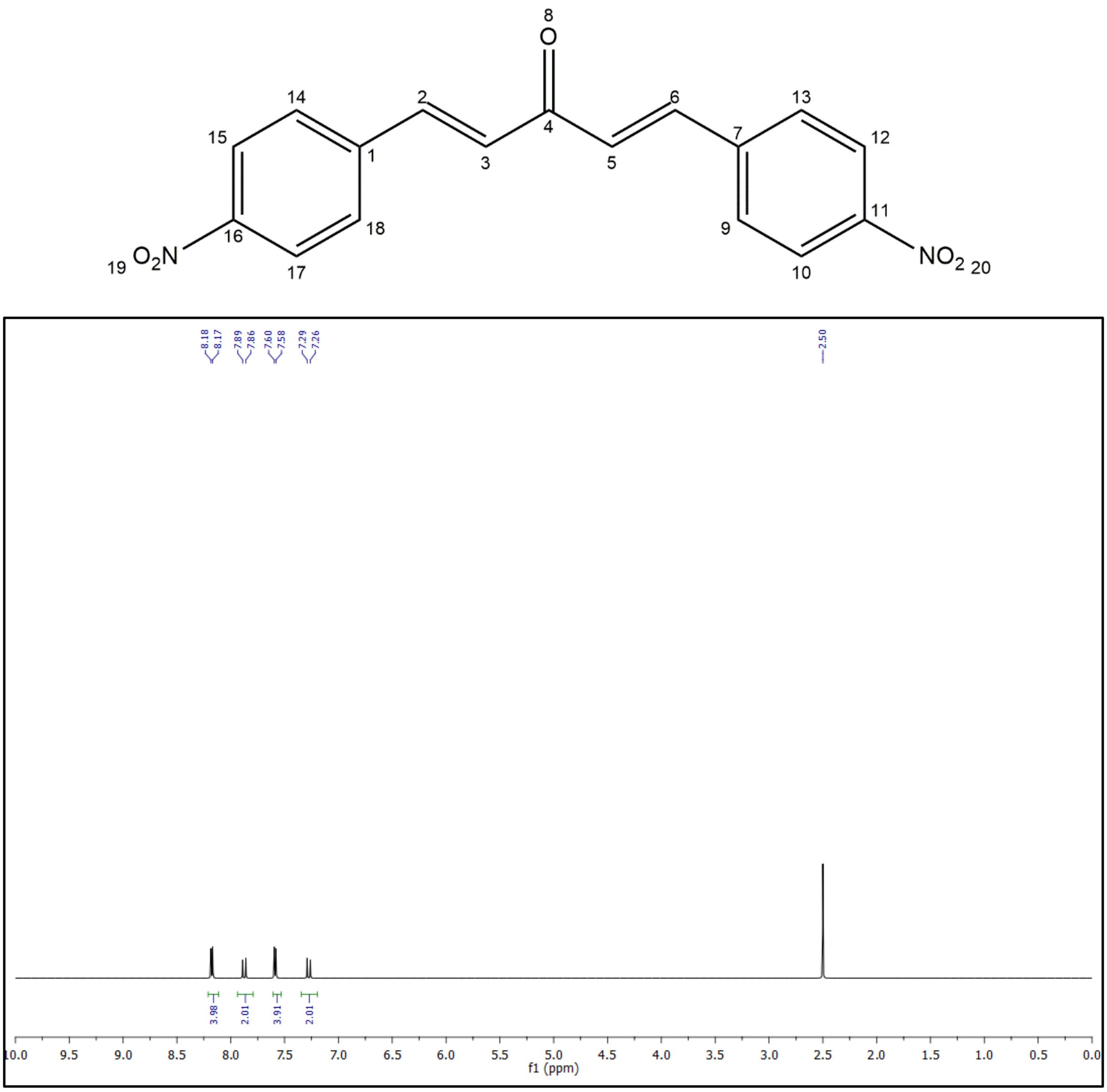

**Figure S34.** $^{13}$C NMR of 1,5-bis(4-nitrophenyl)penta-1,4-dien-3-one (1c)

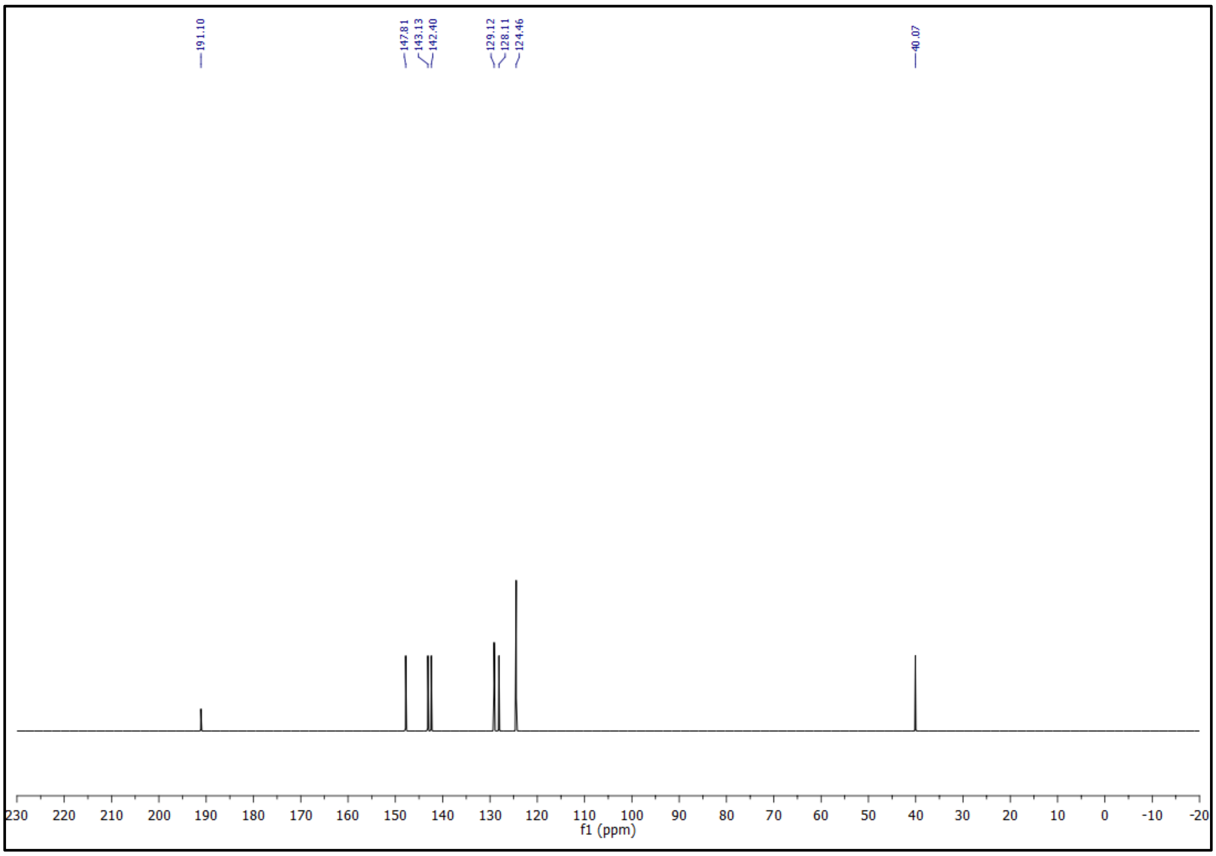

**Figure S35.** $^{1}$H NMR of 1,5-bis(4-hydroxy-3-methoxyphenyl)penta-1,4-dien-3-one (1d)

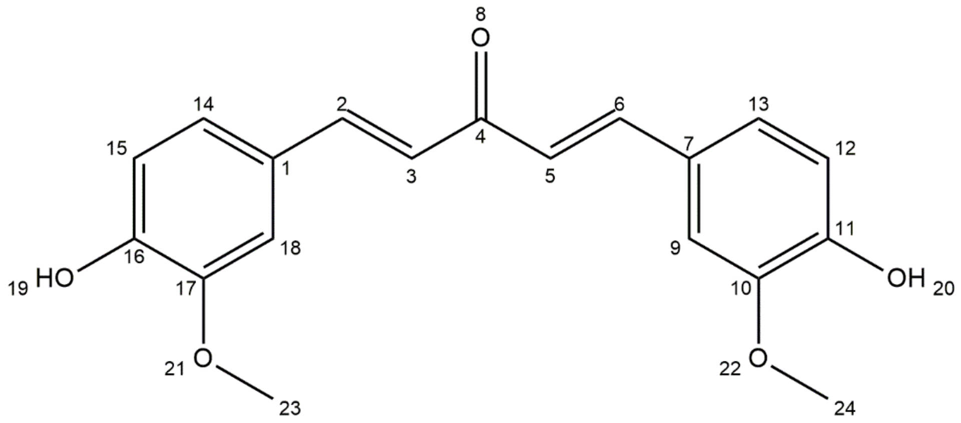


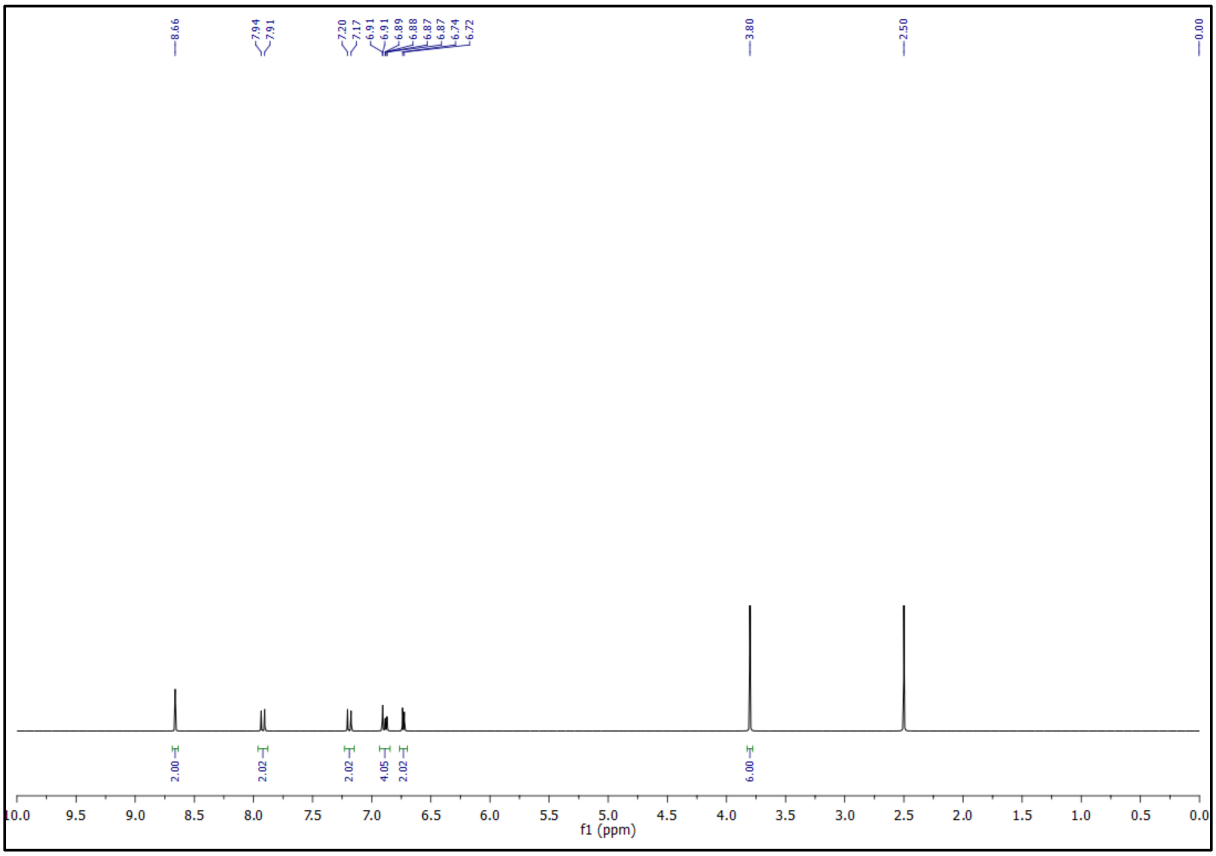

**Figure S36.** $^{13}$C NMR of 1,5-bis(4-hydroxy-3-methoxyphenyl)penta-1,4-dien-3-one (1d)

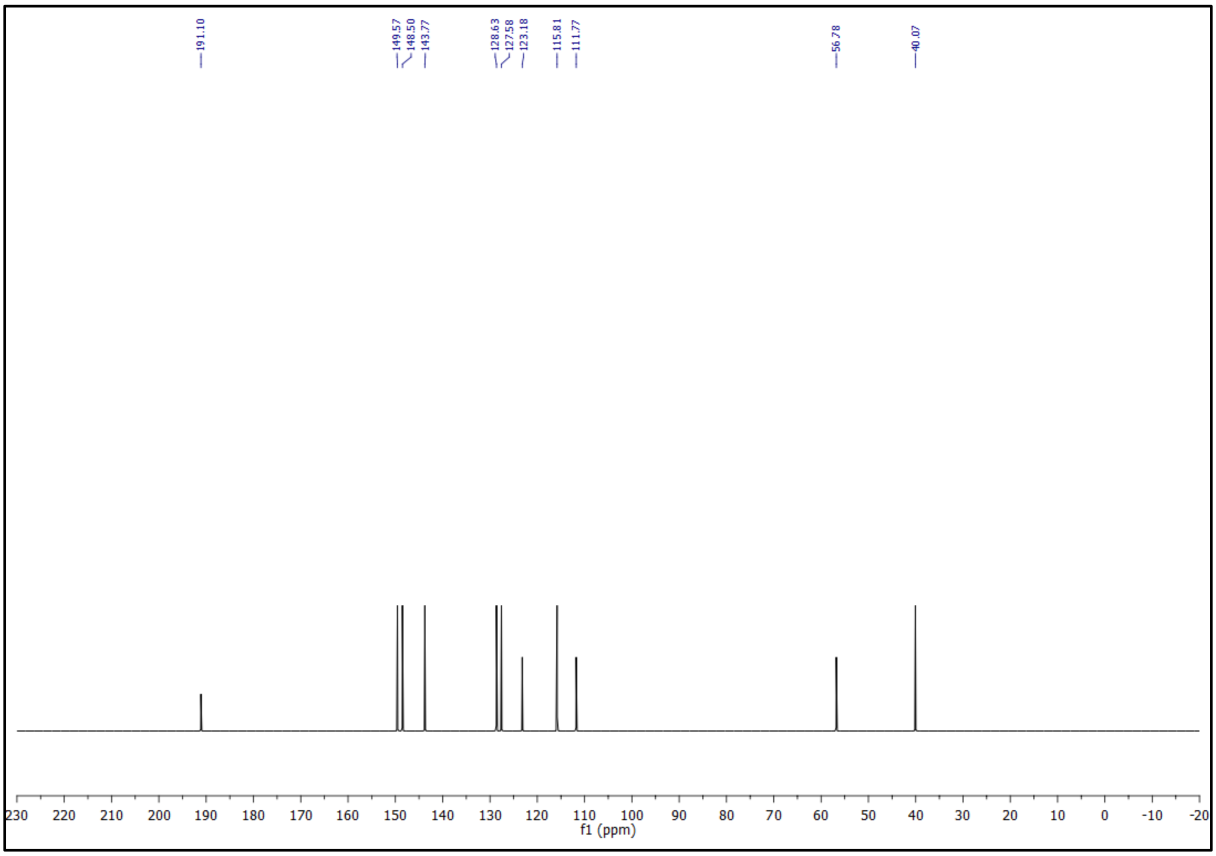

**Figure S37.** $^{1}$H NMR of 1,5-bis(2-hydroxyphenyl)penta-1,4-dien-3-one (1e)

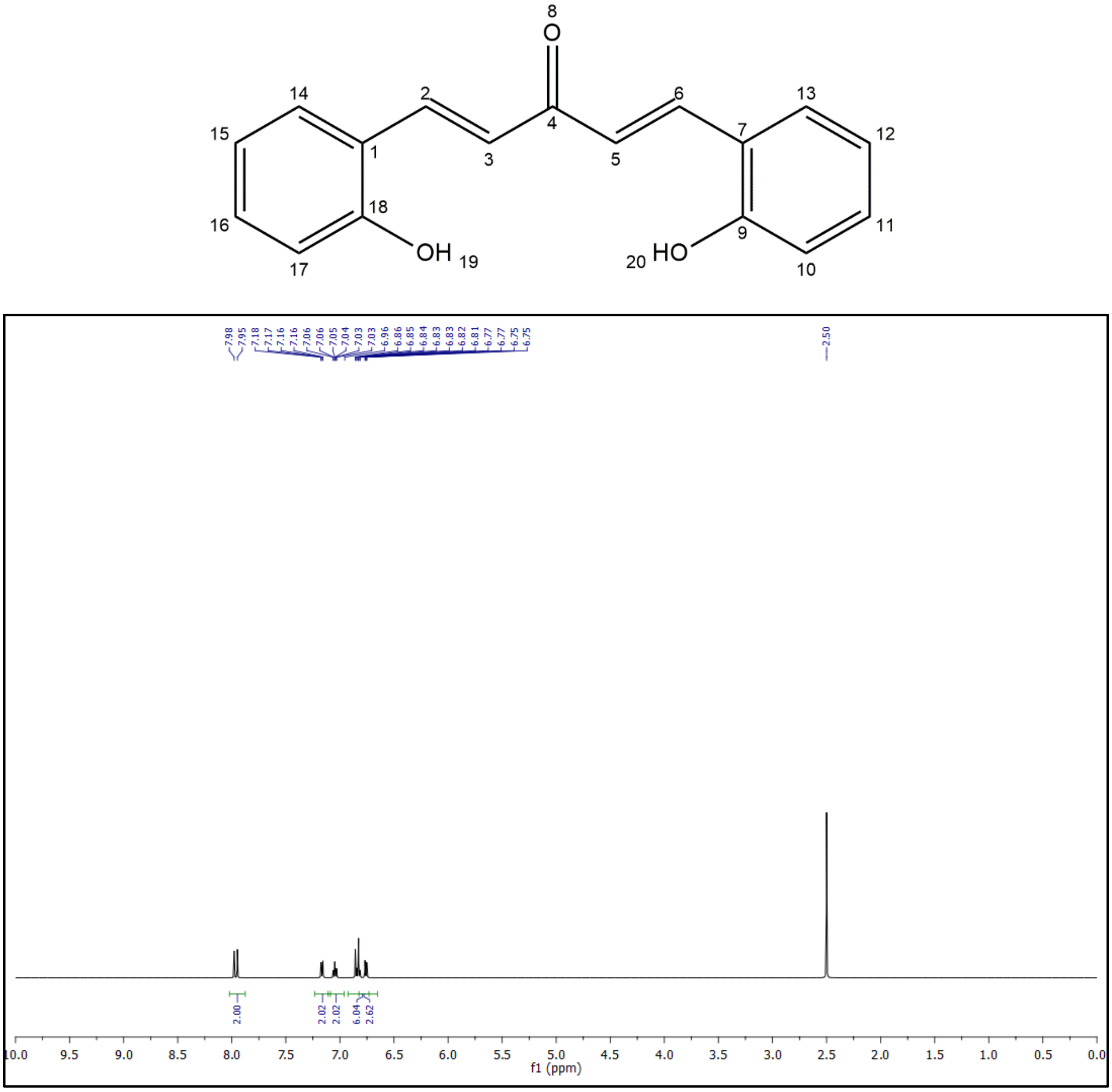

**Figure S38.** $^{13}$C NMR of 1,5-bis(2-hydroxyphenyl)penta-1,4-dien-3-one (1e)

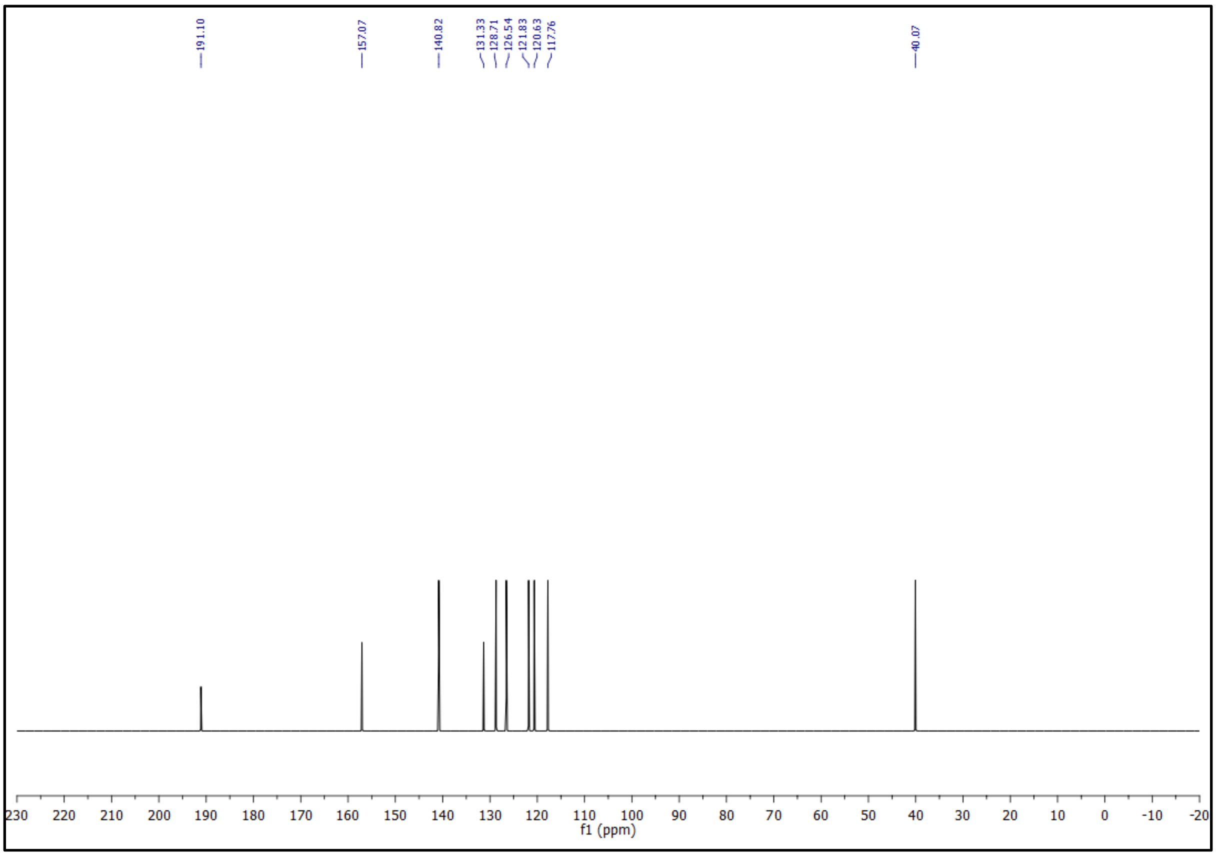

**Figure S39.** $^1$H NMR of 1,5-bis(4-(dimethylamino)phenyl)penta-1,4-dien-3-one (1f)

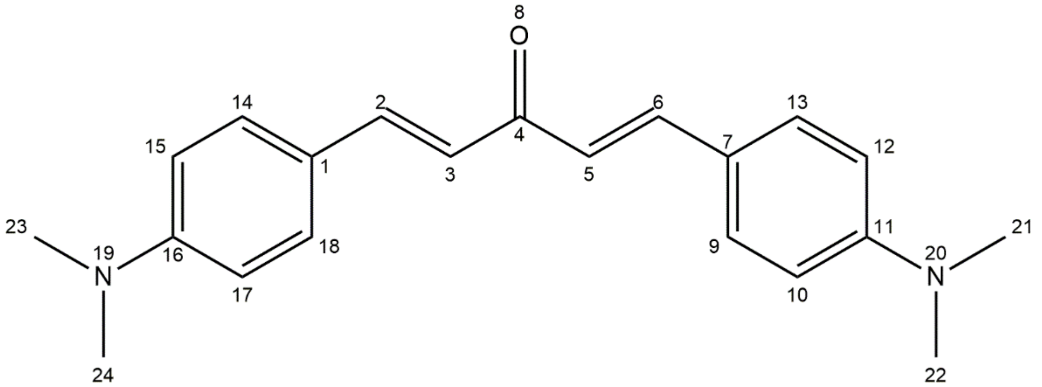


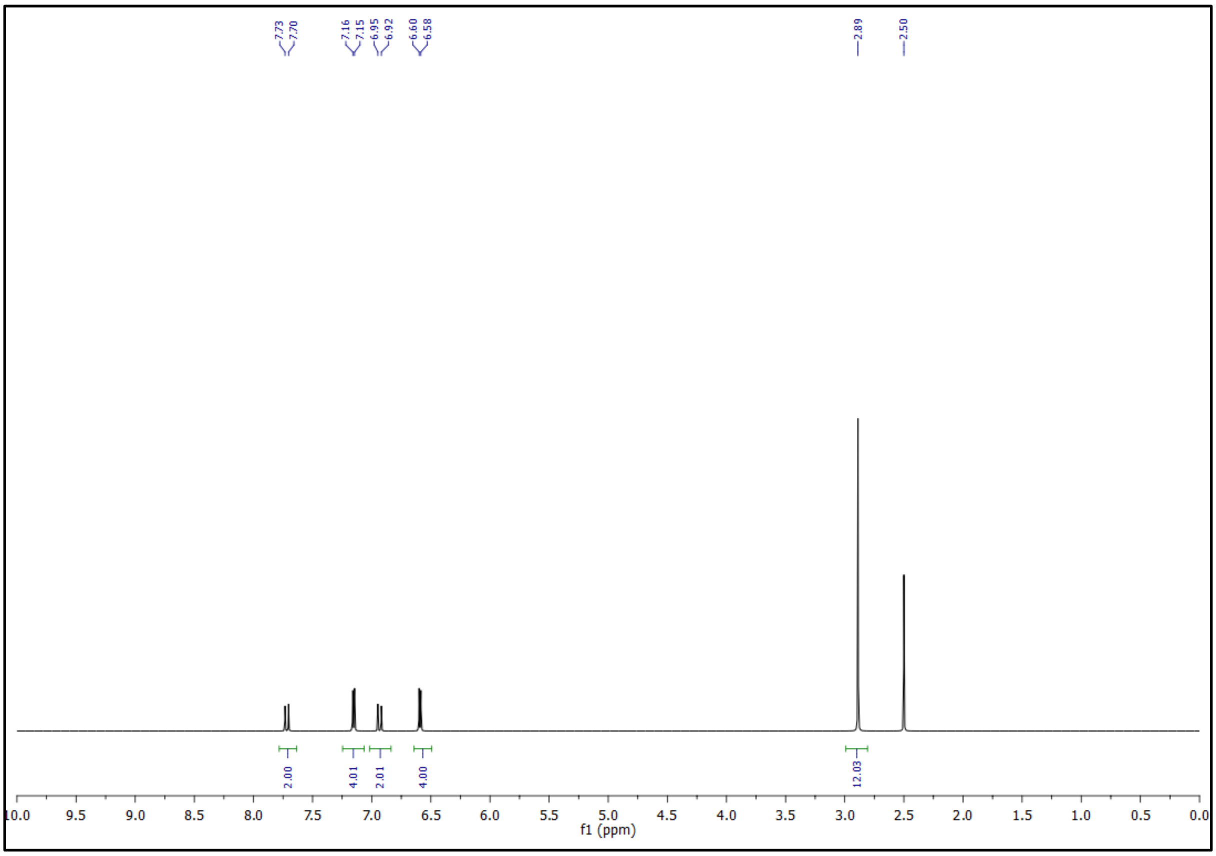

**Figure S40.** $^{13}$C NMR of 1,5-bis(4-(dimethylamino)phenyl)penta-1,4-dien-3-one (1f)

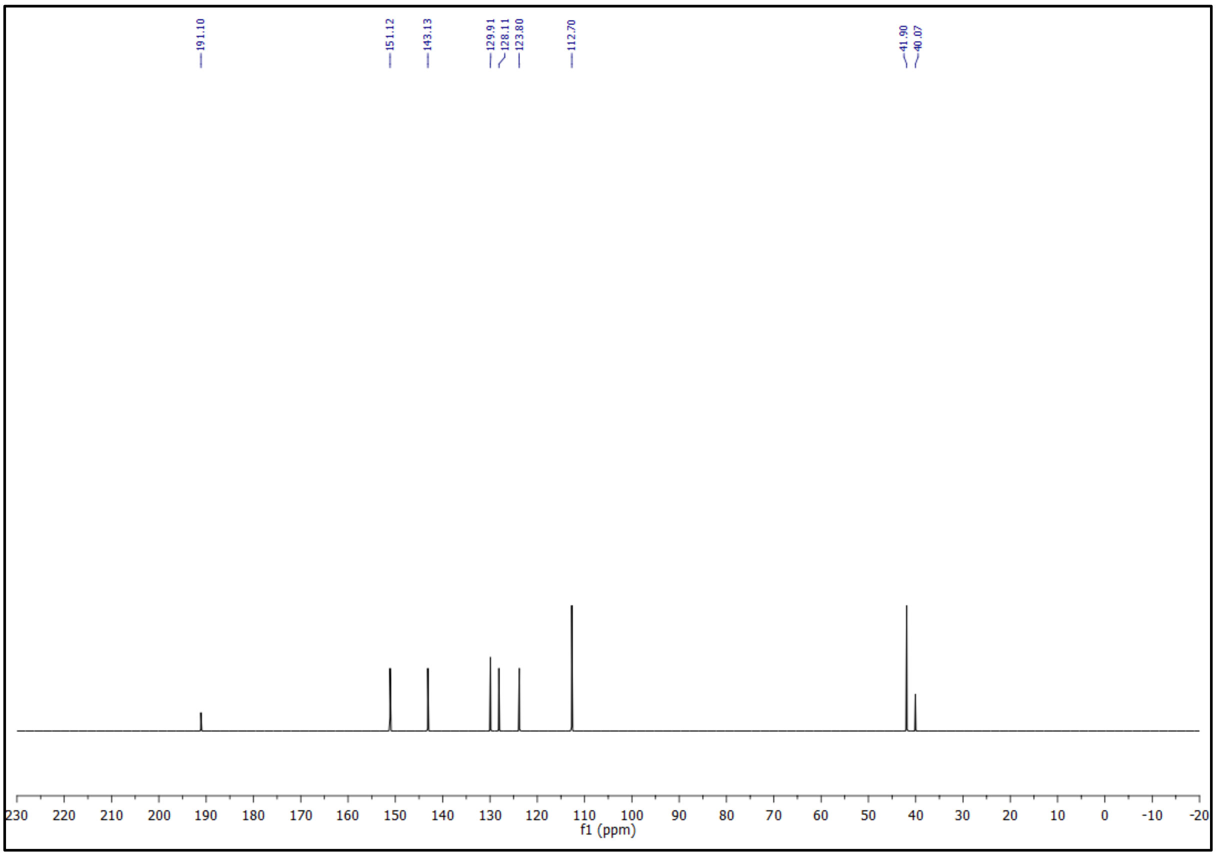

**Figure S41.** $^{1}$H NMR of 1,5-bis(4-chlorophenyl)penta-1,4-dien-3-one (1g)

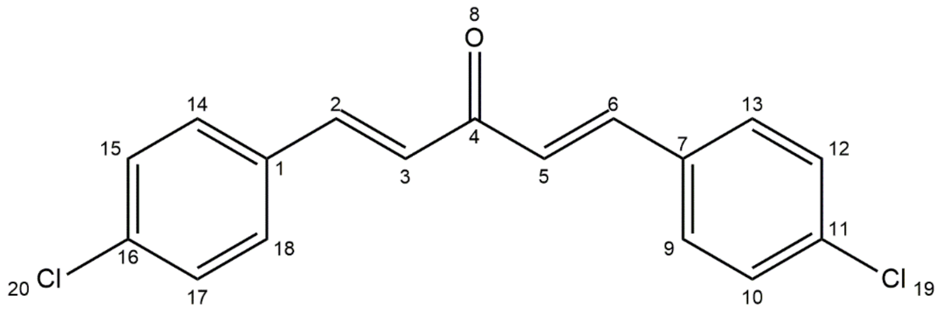

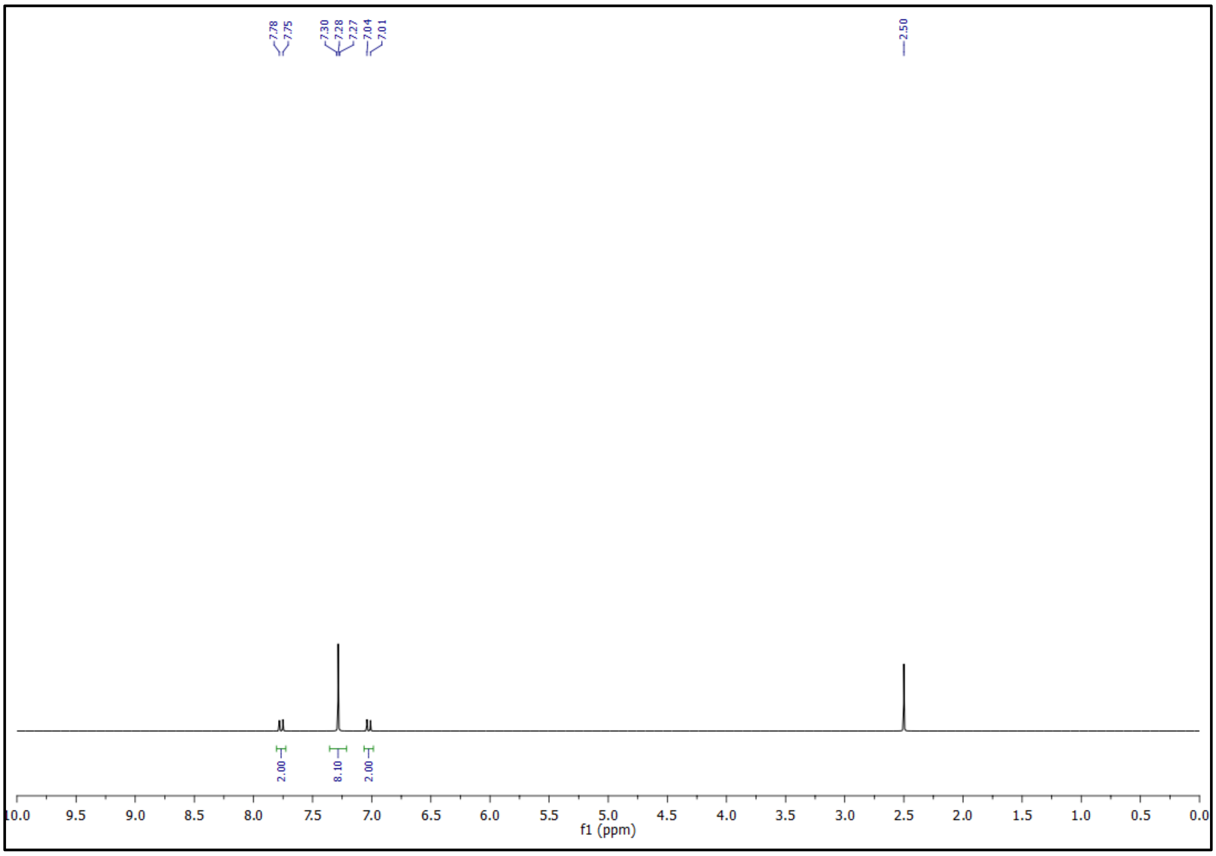

**Figure S42.** $^{13}$C NMR of 1,5-bis(4-chlorophenyl)penta-1,4-dien-3-one (1g)

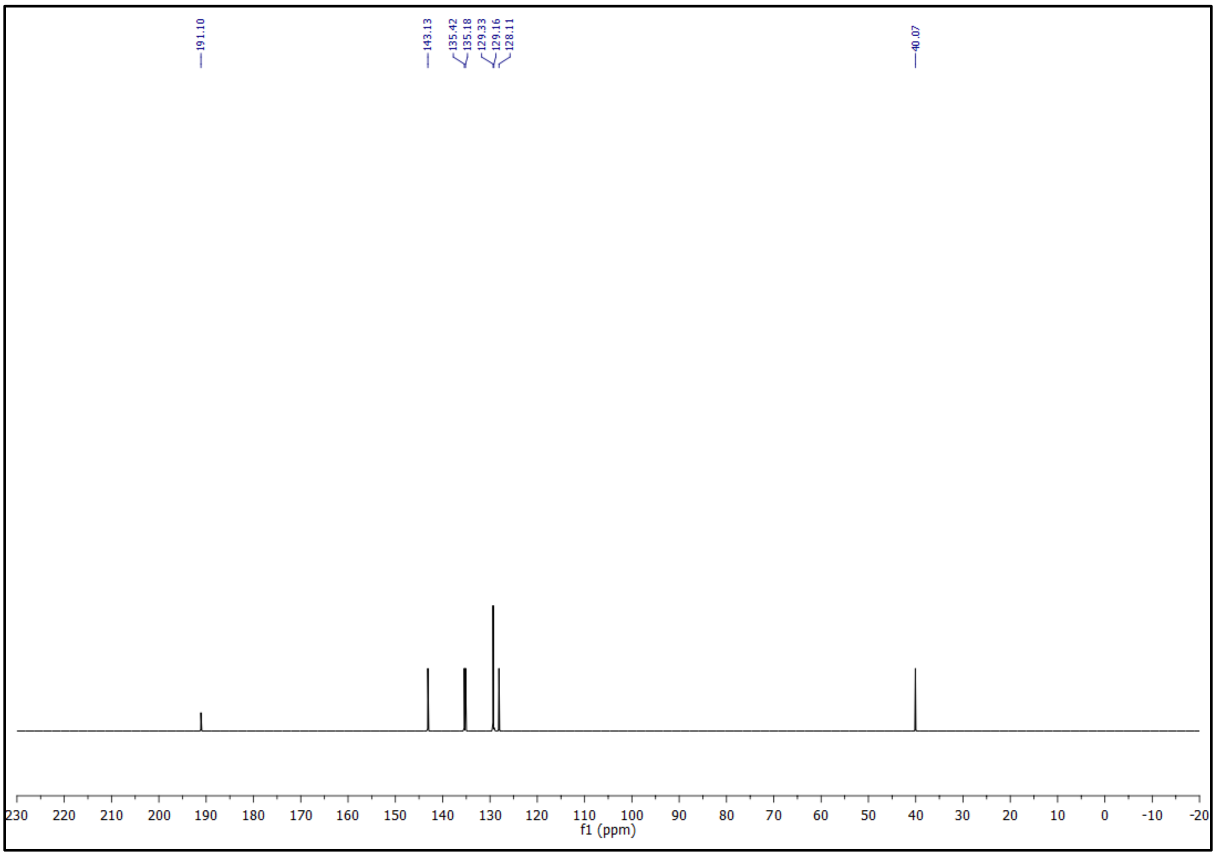

**Figure S43.** $^1$H NMR of *4-(1-(3,5-dinitrophenyl)-3-(4-hydroxystyryl)-4,5-dihydro-1H-pyrazol-5-yl)phenol (IIIab)*

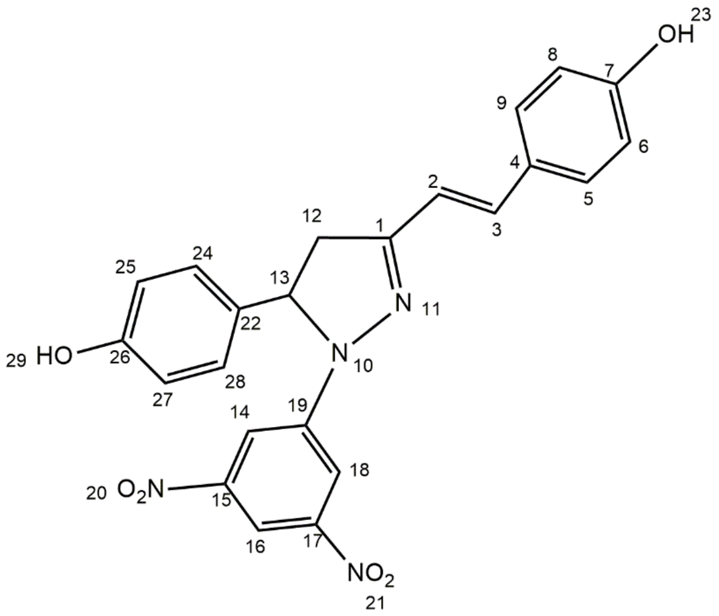

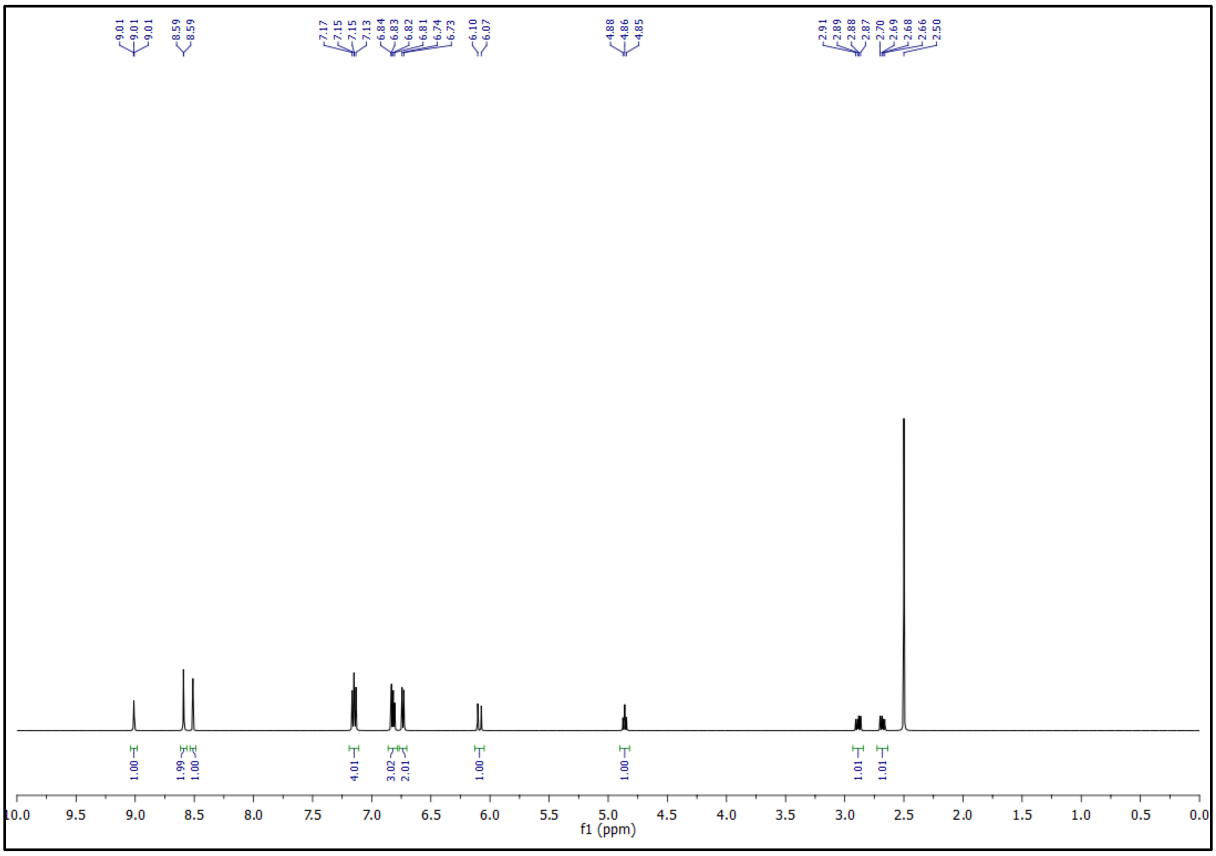

**Figure S44.** $^{13}$C NMR of *4-(1-(3,5-dinitrophenyl)-3-(4-hydroxystyryl)-4,5-dihydro-1H-pyrazol-5-yl)phenol (IIIab)*

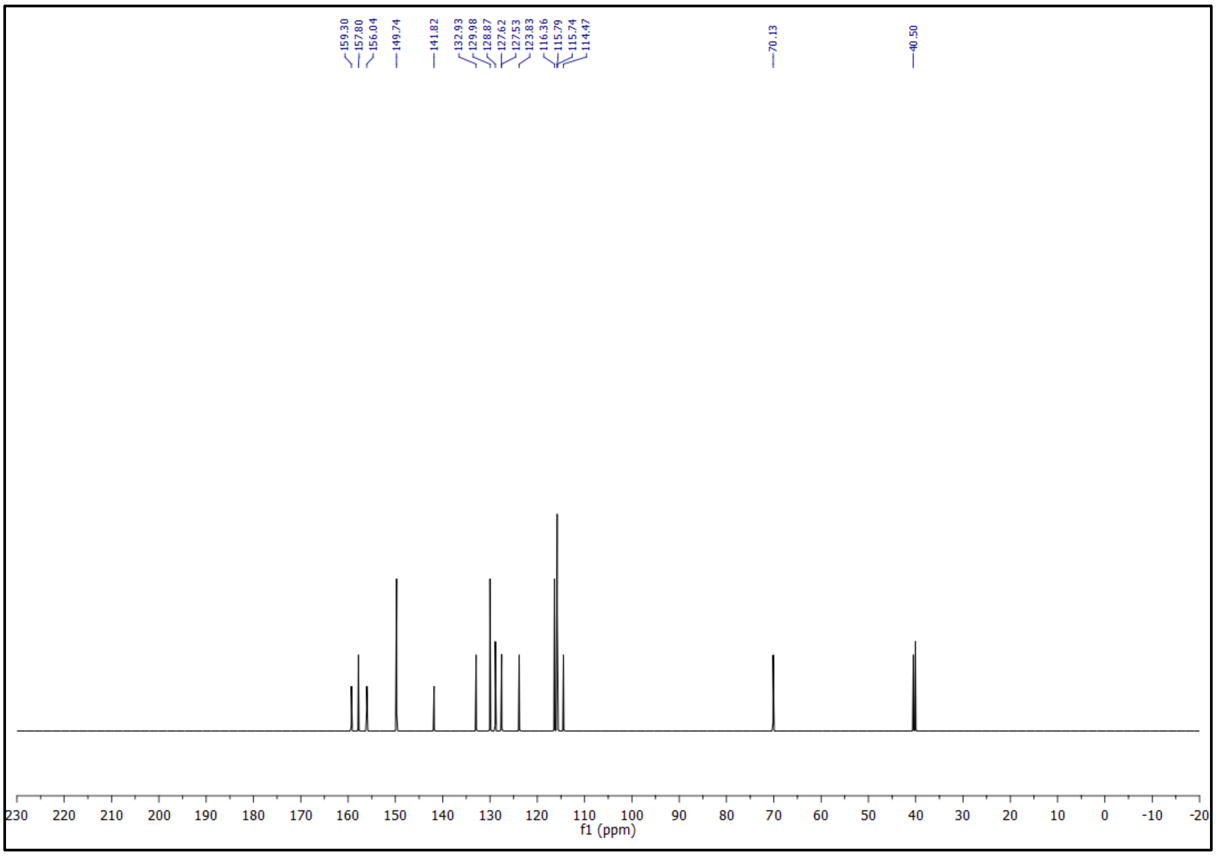

**Figure S45.** $^{1}$H NMR of *4,4'-(3-(2-(2,4-dinitrophenyl)hydrazono)penta-1,4-diene-1,5-diyl)diphenol (IIab)*

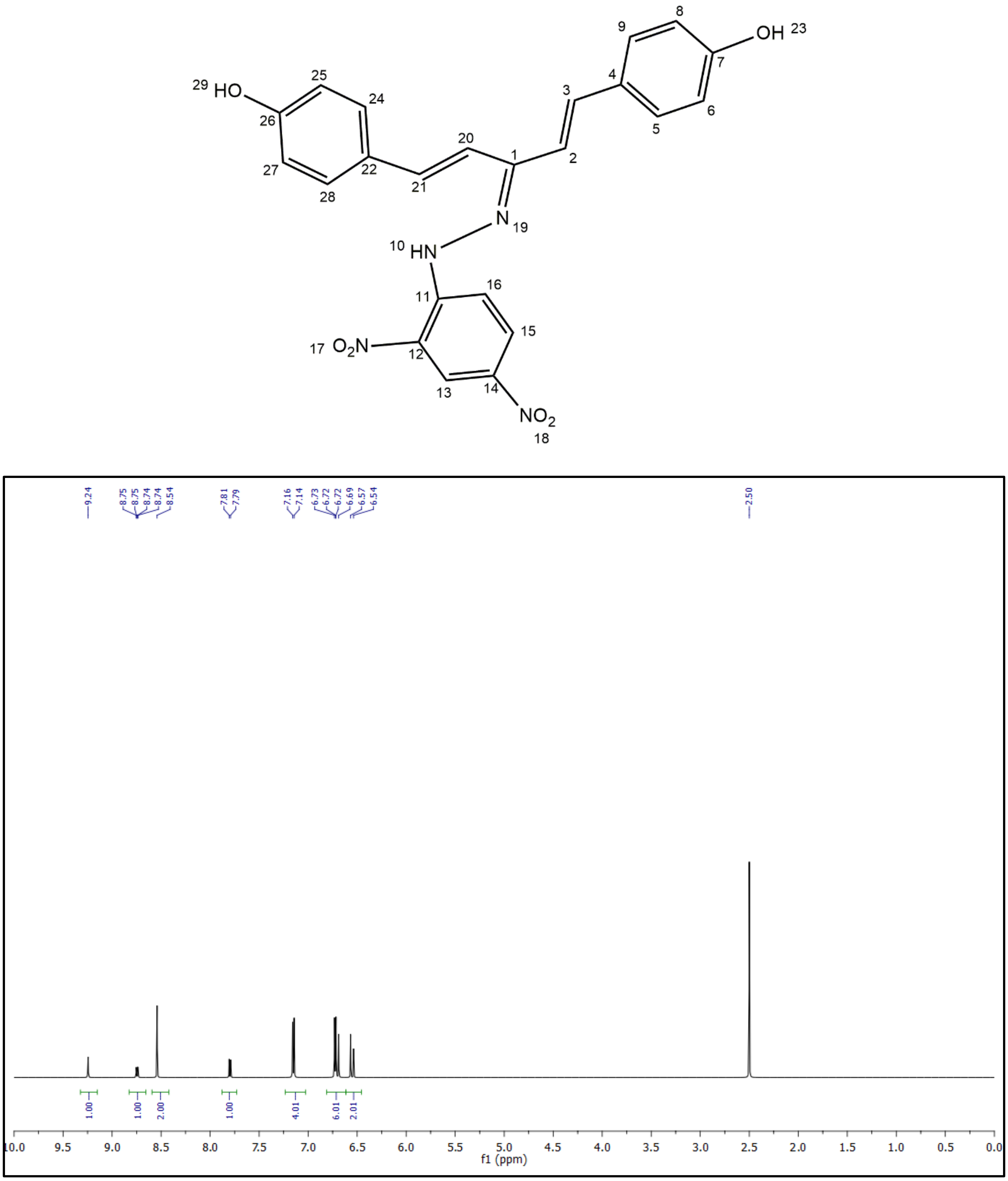

**Figure S46.** $^{13}$C NMR of *4,4'-(3-(2-(2,4-dinitrophenyl)hydrazono)penta-1,4-diene-1,5-diyl)diphenol (IIab)*

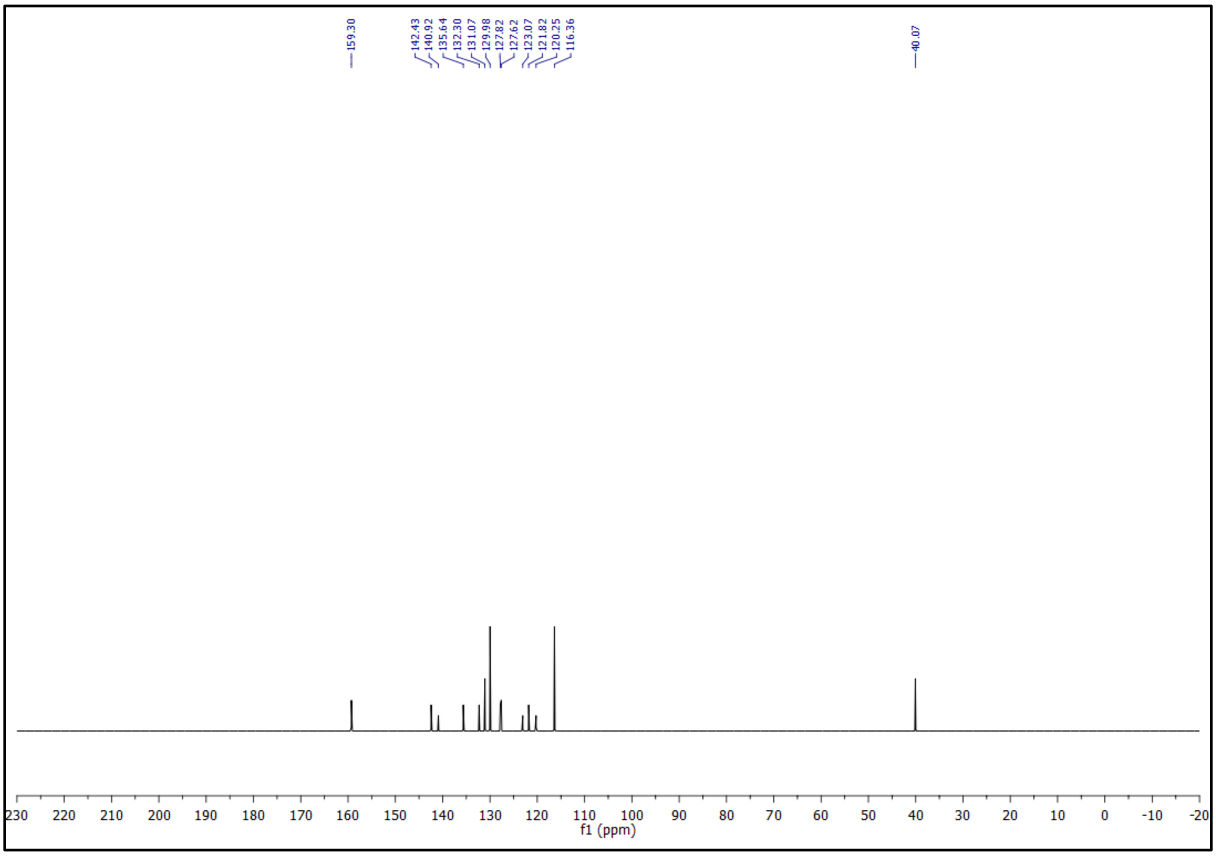

**Figure S47.** 2D (left) and 3D (right) representations of closest binding interaction between synthesized ligands (4a-4n) and receptor protein 2QV4.

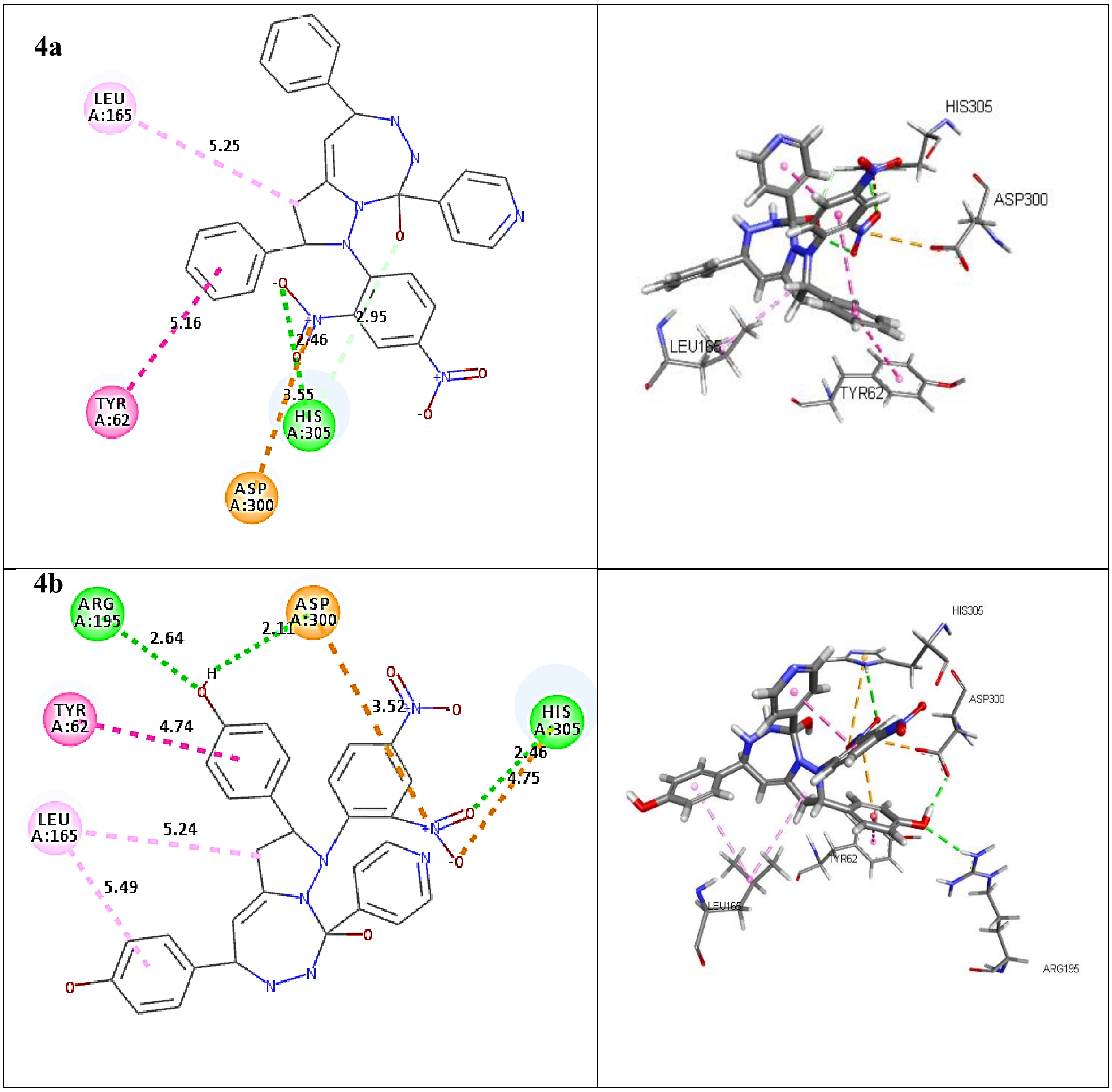

4c

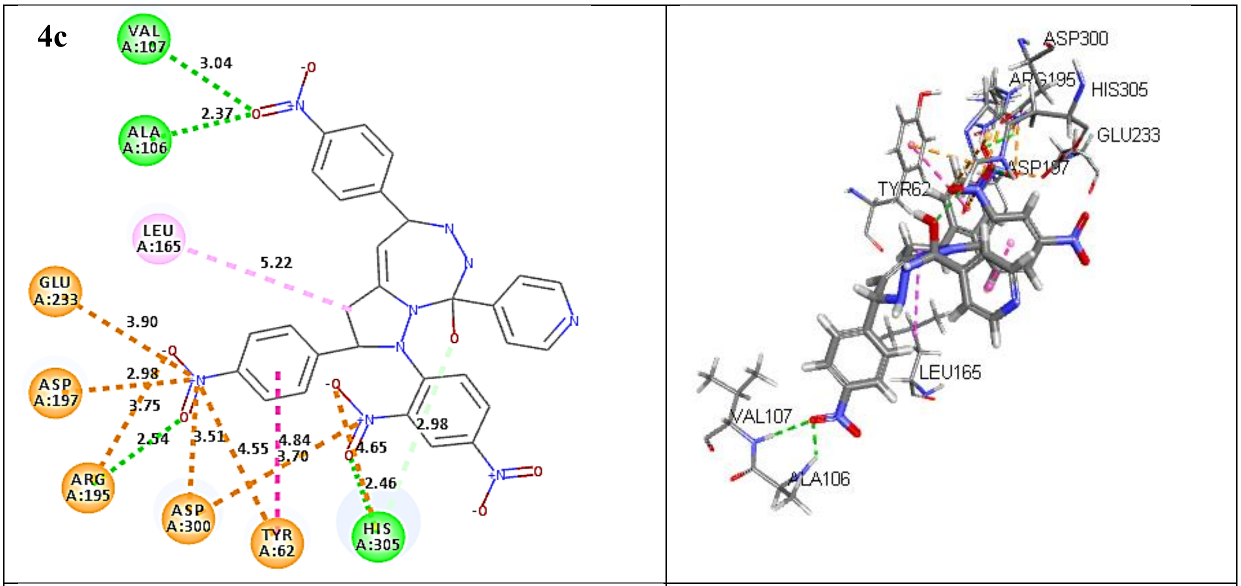
VAL A:107
ALA A:106
LEU A:165
GLU A:233
ASP A:197
ARG A:195
ASP A:300
TYR A:62
HIS A:305
3.04
2.37
5.22
3.90
2.98
3.75
2.54
3.51
4.55
4.84
3.70
4.65
2.98
2.46
ASP300
ARG195
HIS305
GLU233
ASP197
TYR62
LEU165
VAL107
ALA106

4e

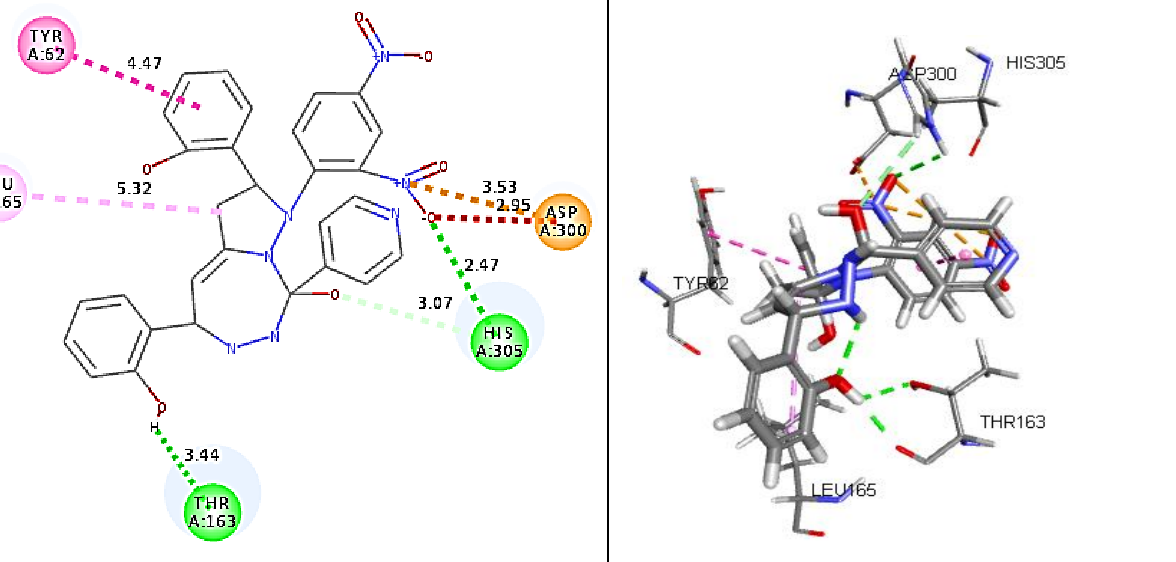
TYR A:62
LEU A:165
ASP A:300
HIS A:305
THR A:163
4.47
5.32
3.53
2.95
2.47
3.07
3.44
ASP300
HIS305
TYR62
THR163
LEU165

4g

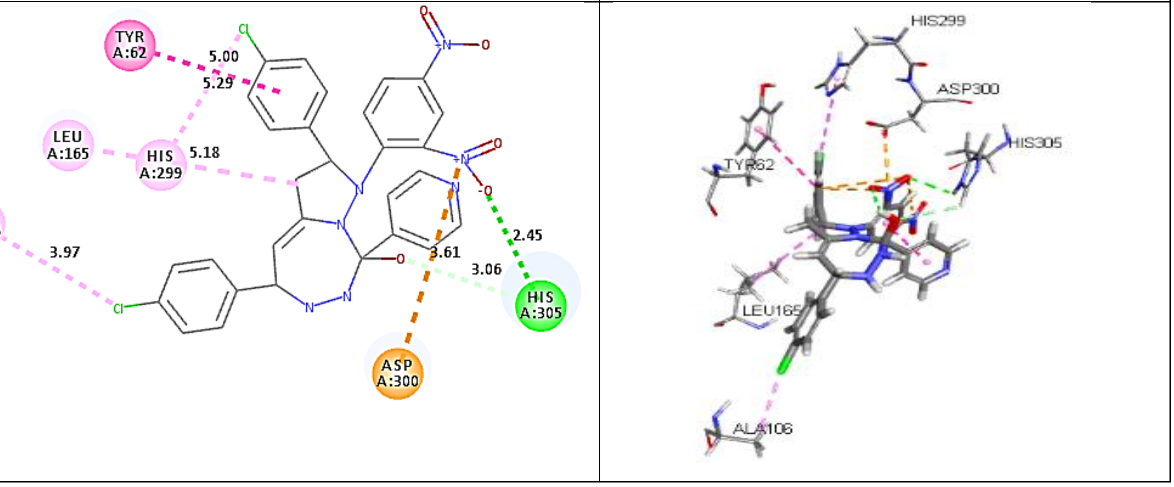
TYR A:62
LEU A:165
HIS A:299
ALA A:106
ASP A:300
HIS A:305
5.00
5.29
5.18
3.97
3.61
3.06
2.45
HIS299
ASP300
HIS305
TYR62
LEU165
ALA106

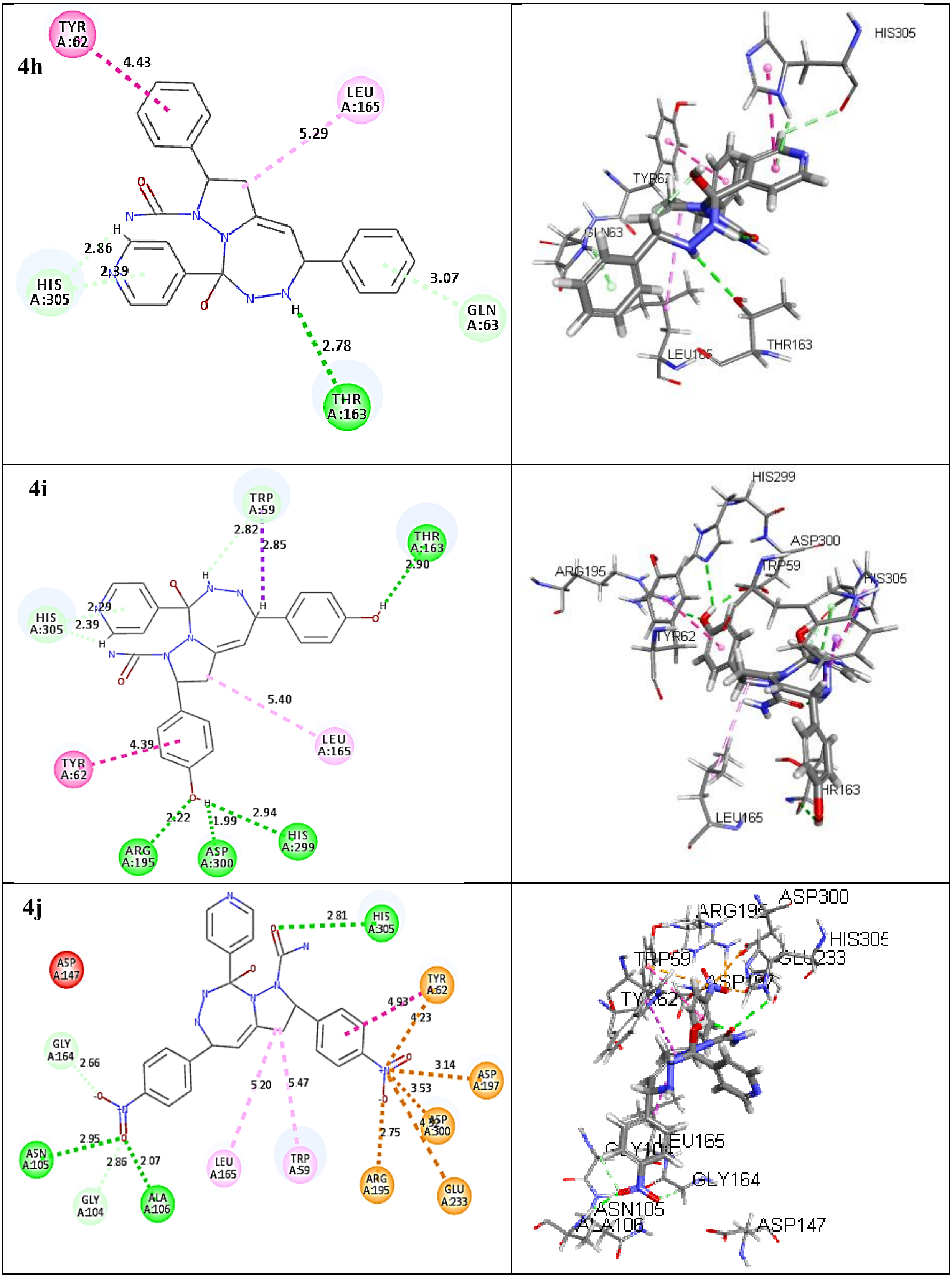
4h
TYR A:62
4.43
LEU A:165
5.29
2.86
2.39
HIS A:305
3.07
GLN A:63
2.78
THR A:163
HIS305
TYR62
GLN63
LEU165
THR163
4i
TRP A:59
2.82
2.85
THR A:163
2.90
HIS A:305
2.29
2.39
5.40
LEU A:165
4.39
TYR A:62
2.22
1.99
2.94
ARG A:195
ASP A:300
HIS A:299
HIS299
ASP300
ARG195
TRP59
HIS305
TYR62
LEU165
4j
2.81
HIS A:305
ASP A:147
TYR A:62
4.93
4.23
GLY A:164
2.66
3.14
ASP A:197
5.20
5.47
3.53
2.95
2.86
2.07
2.75
ASN A:105
GLY A:104
ALA A:106
LEU A:165
TRP A:59
ARG A:195
ASP A:300
GLU A:233
ASP300
HIS305
TRP59
GLY164
ASN105
ASP147

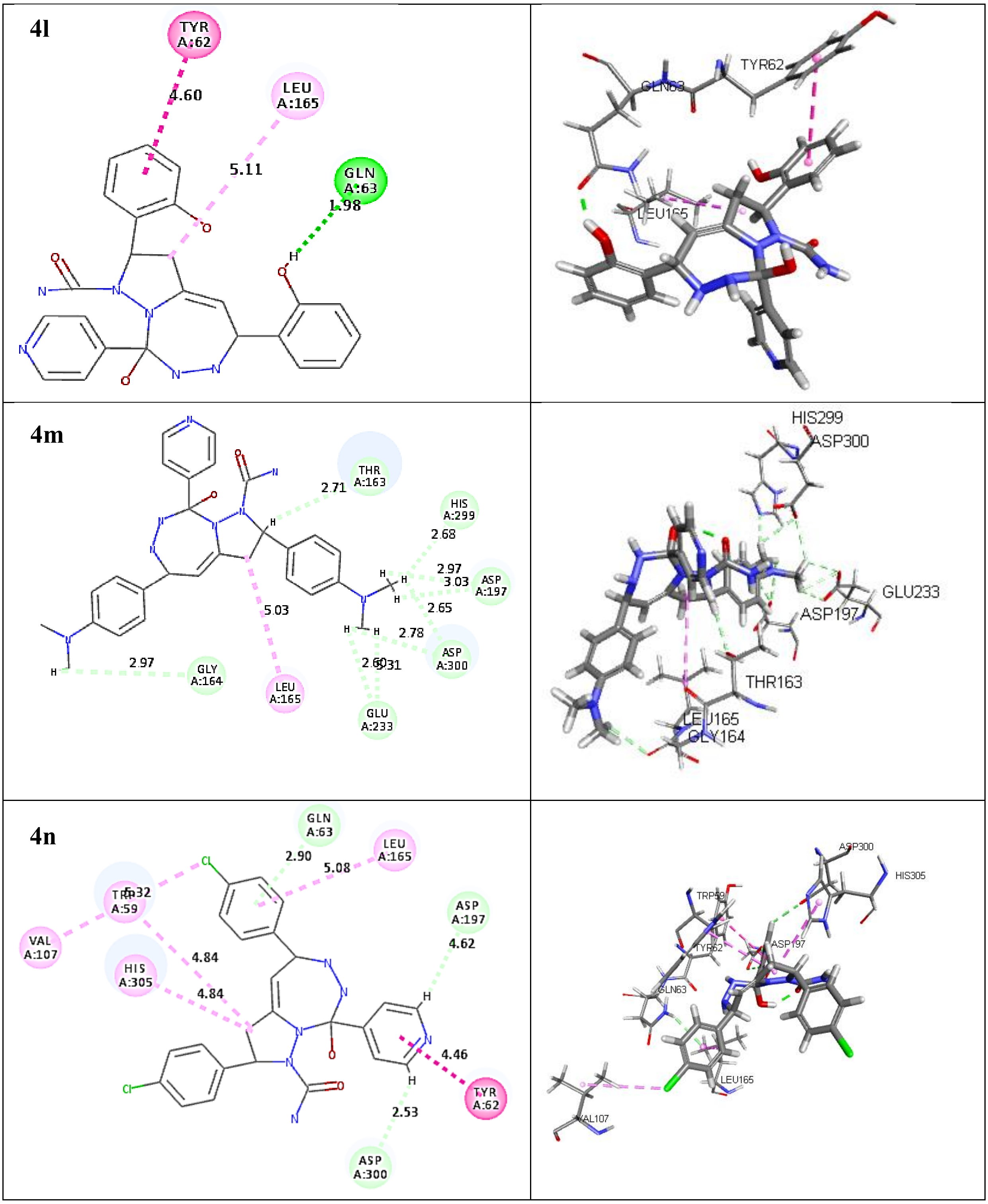


**Figure S48.** 2D (left) and 3D (right) representations of closest binding interaction between synthesized ligands (4a-4n) and receptor protein 3W37.

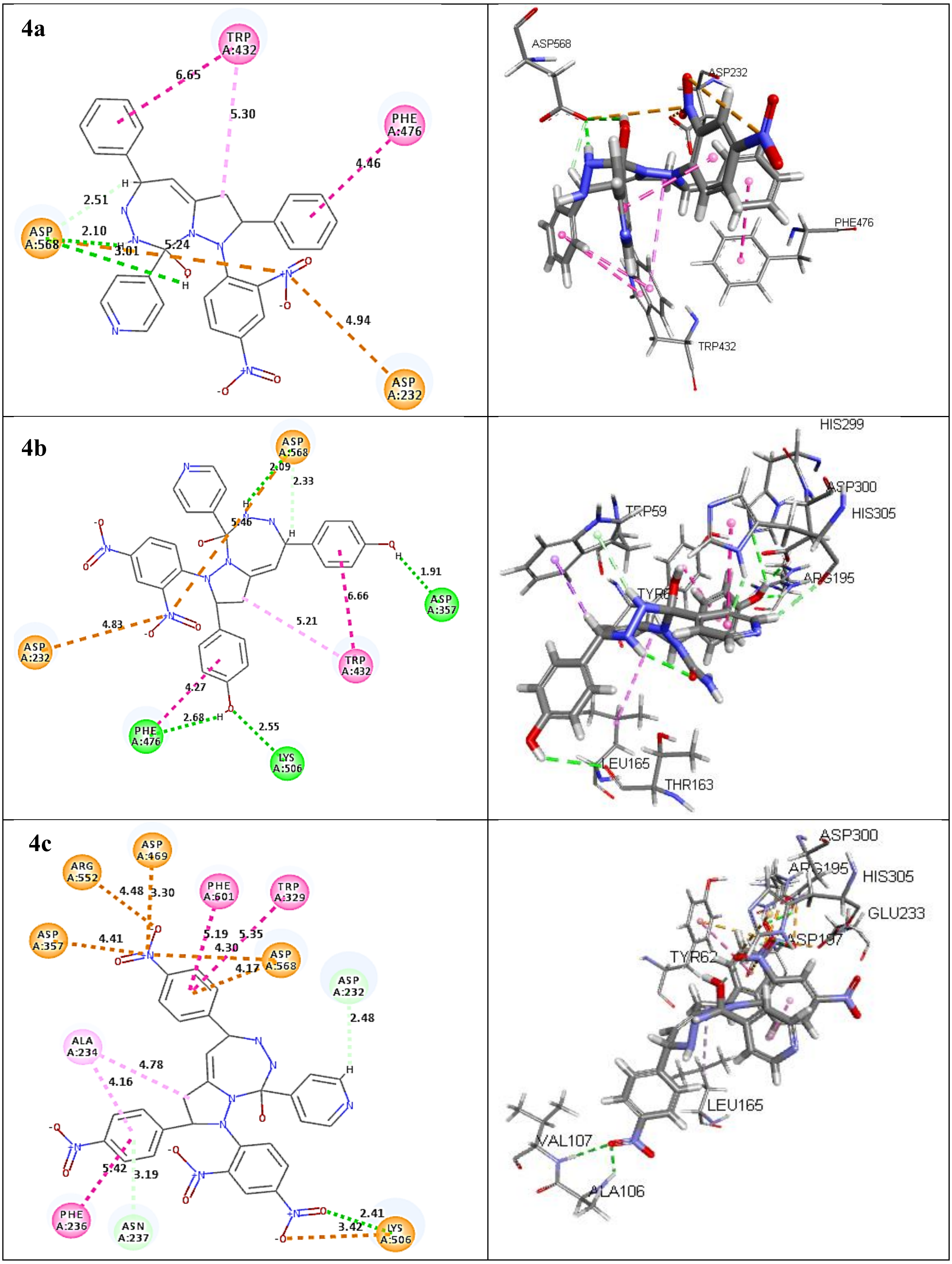

4a
TRP A:432
6.65
5.30
PHE A:476
4.46
2.51
ASP A:568
2.10
3.01
5.24
4.94
ASP A:232
ASP568
ASP232
PHE476
TRP432
4b
ASP A:568
2.09
2.33
5.46
1.91
ASP A:357
6.66
4.83
ASP A:232
5.21
TRP A:432
4.27
2.68
2.55
PHE A:476
LYS A:506
HIS299
ASP300
HIS305
THR163
4c
ARG A:552
ASP A:469
PHE A:601
TRP A:329
4.48
3.30
ASP A:357
4.41
5.19
5.35
4.30
4.17
ASP A:568
ASP A:232
2.48
ALA A:234
4.78
4.16
5.42
3.19
PHE A:236
ASN A:237
2.41
3.42
LYS A:506
ASP300
HIS305
GLU233
LEU165
VAL107

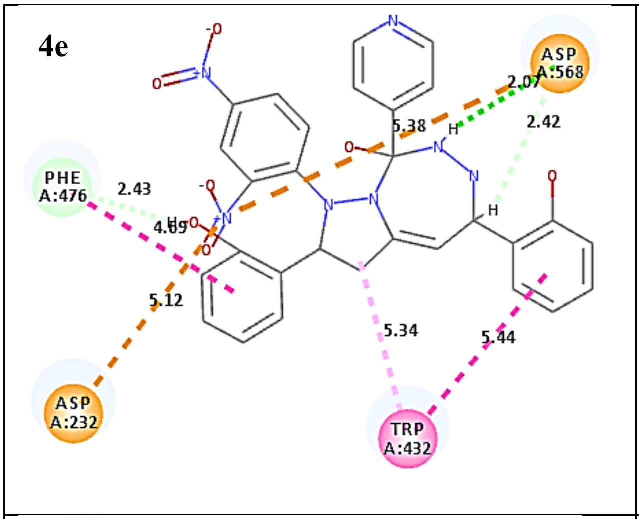
4e
ASP A:568
2.07
5.38
2.42
PHE A:476
2.43
4.69
5.12
5.34
5.44
ASP A:232
TRP A:432

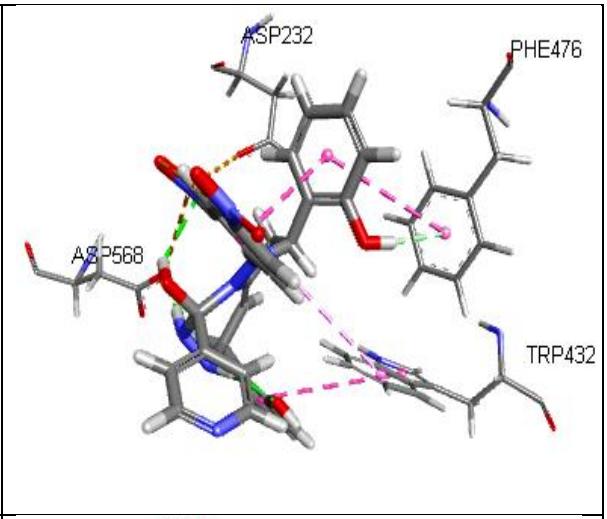
ASP232
PHE476
ASP568
TRP432

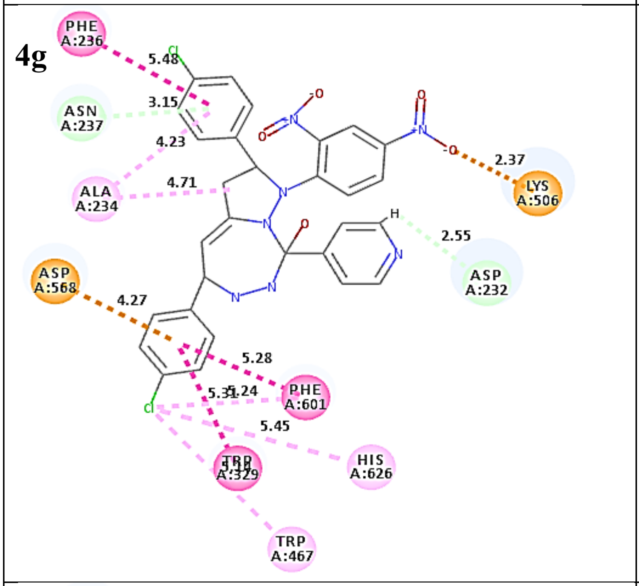
4g
PHE A:236
5.48
ASN A:237
3.15
4.23
ALA A:234
4.71
2.37
LYS A:506
2.55
ASP A:232
ASP A:568
4.27
5.28
PHE A:601
5.45
HIS A:626
TRP A:329
TRP A:467

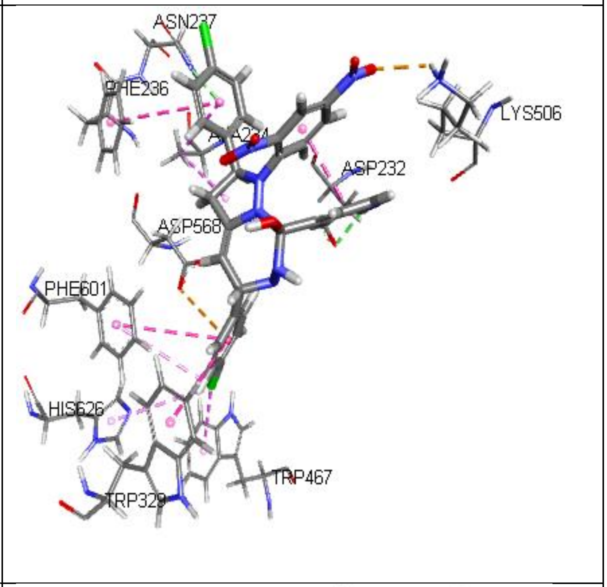
LYS506
ASP232
ASP568
PHE601
HIS626
TRP329
TRP467

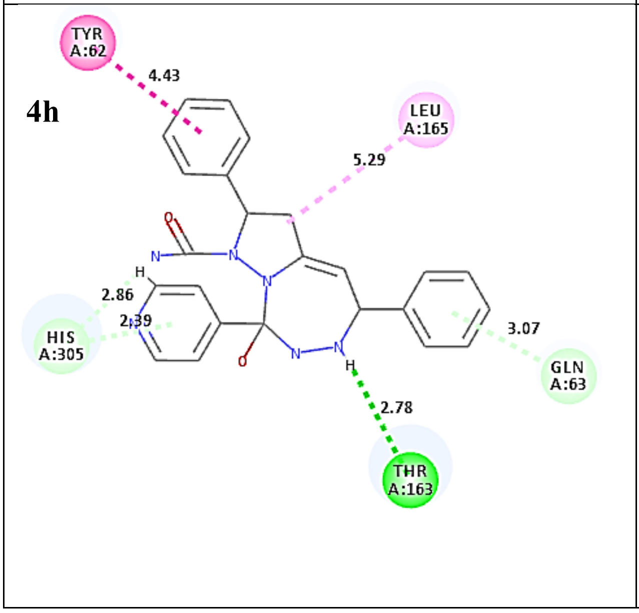
4h
TYR A:62
4.43
LEU A:165
5.29
2.86
HIS A:305
3.07
GLN A:63
2.78
THR A:163

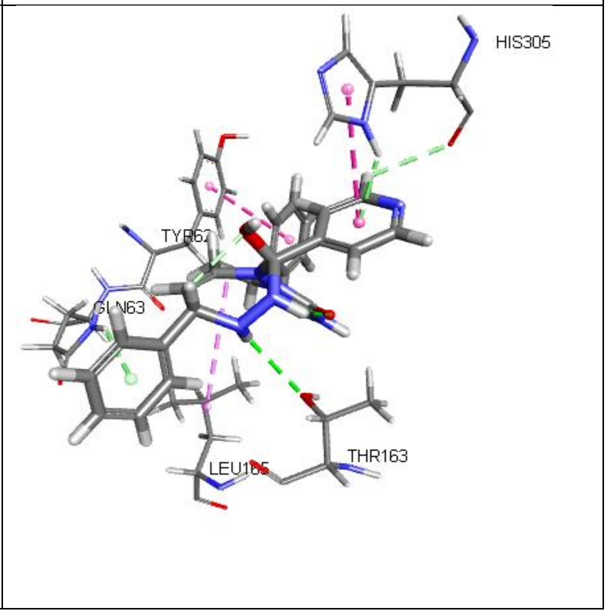
HIS305
THR163

**4h**

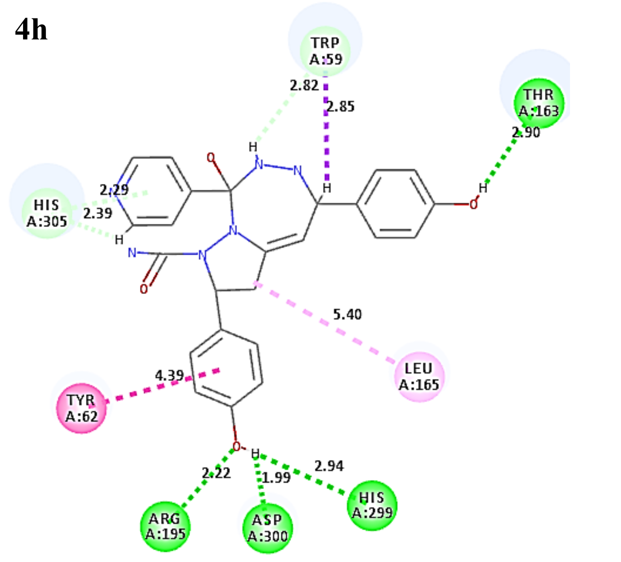
TRP A:59
2.82
2.85
THR A:163
2.90
HIS A:305
2.29
2.39
5.40
LEU A:165
4.39
TYR A:62
2.22
1.99
2.94
ARG A:195
ASP A:300
HIS A:299

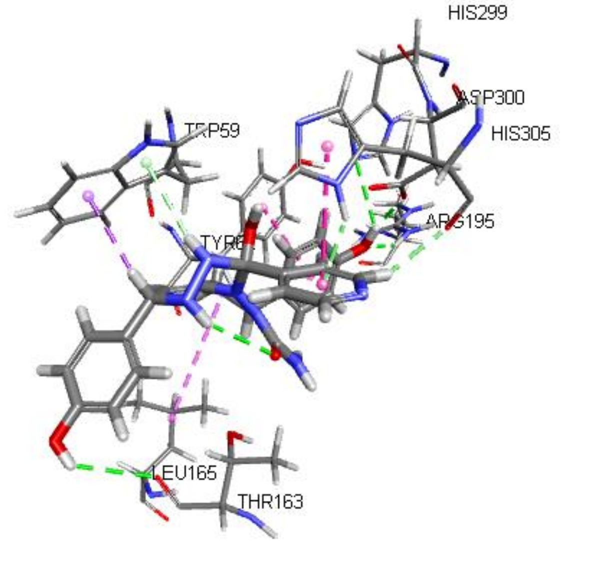
HIS299
ASP300
HIS305
TRP59
LEU165
THR163

**4j**

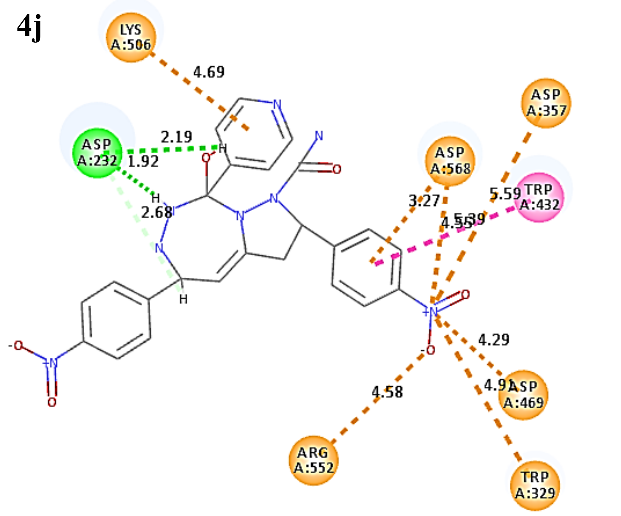
LYS A:506
4.69
ASP A:357
ASP A:232
2.19
1.92
2.68
ASP A:568
3.27
5.59
TRP A:432
4.29
4.58
ASP A:469
ARG A:552
TRP A:329

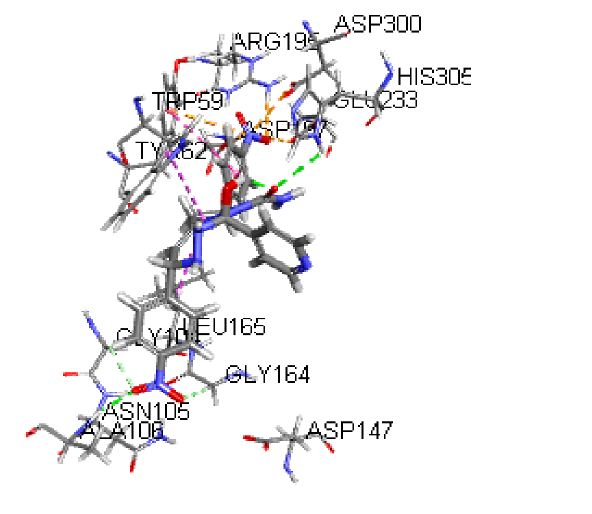
ASP300
HIS305
TRP59
LEU165
GLY164
ASN105
ASP147

**4k**

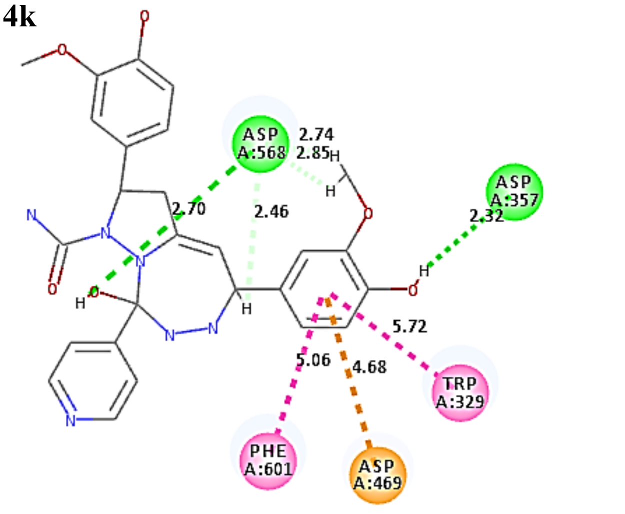
ASP A:568
2.74
2.85
2.70
2.46
ASP A:357
2.32
5.72
5.06
4.68
TRP A:329
PHE A:601
ASP A:469

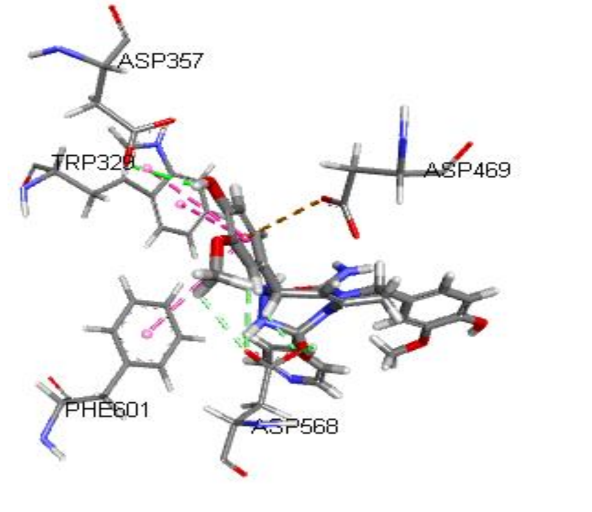
ASP357
ASP469
PHE601
ASP568

| | |
|---|---|
| **4l** 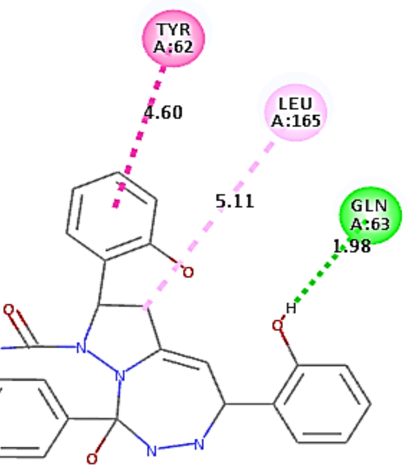 | 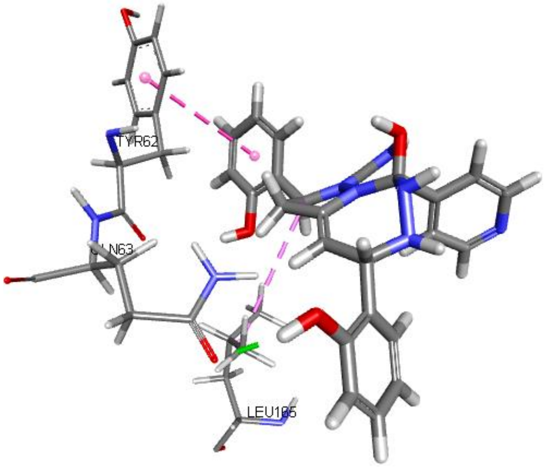 |
| **4m** 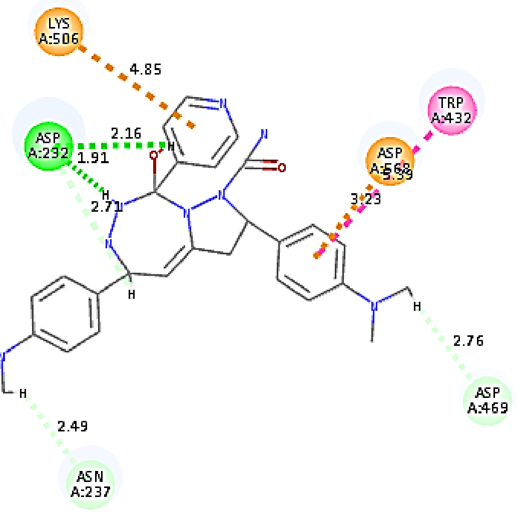 | 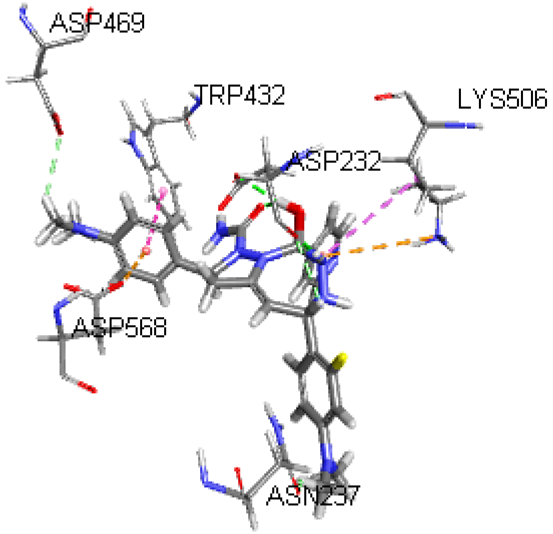 |
| **4n** 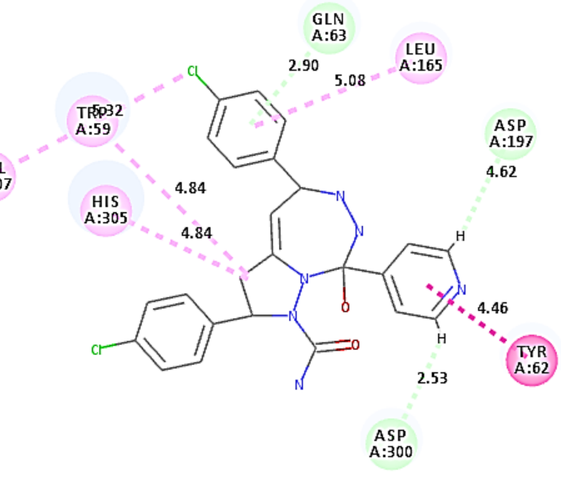 | 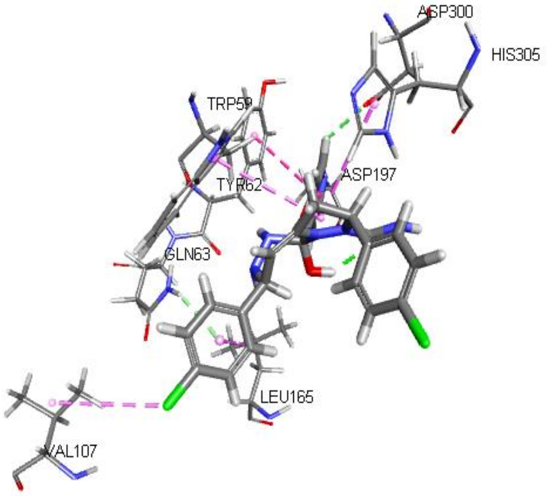 |


TYR A:62
4.60
LEU A:165
5.11
GLN A:63
1.98



TYR62
LEU165



LYS A:506
4.85
ASP A:232
2.16
1.91
2.71
TRP A:432
ASP A:568
3.23
2.76
ASP A:469
2.49
ASN A:237



ASP469
TRP432
LYS506
ASP232
ASP568
ASN237



GLN A:63
2.90
LEU A:165
5.08
TRP A:59
VAL A:107
HIS A:305
4.84
4.84
ASP A:197
4.62
4.46
TYR A:62
2.53
ASP A:300



ASP300
HIS305
TRP59
TYR62
ASP197
GLN63
LEU165
VAL107

**Figure S49.** Molecular orbitals diagram (HOMO; bottom and LUMO; top) with HOMO-LUMO energy gap.

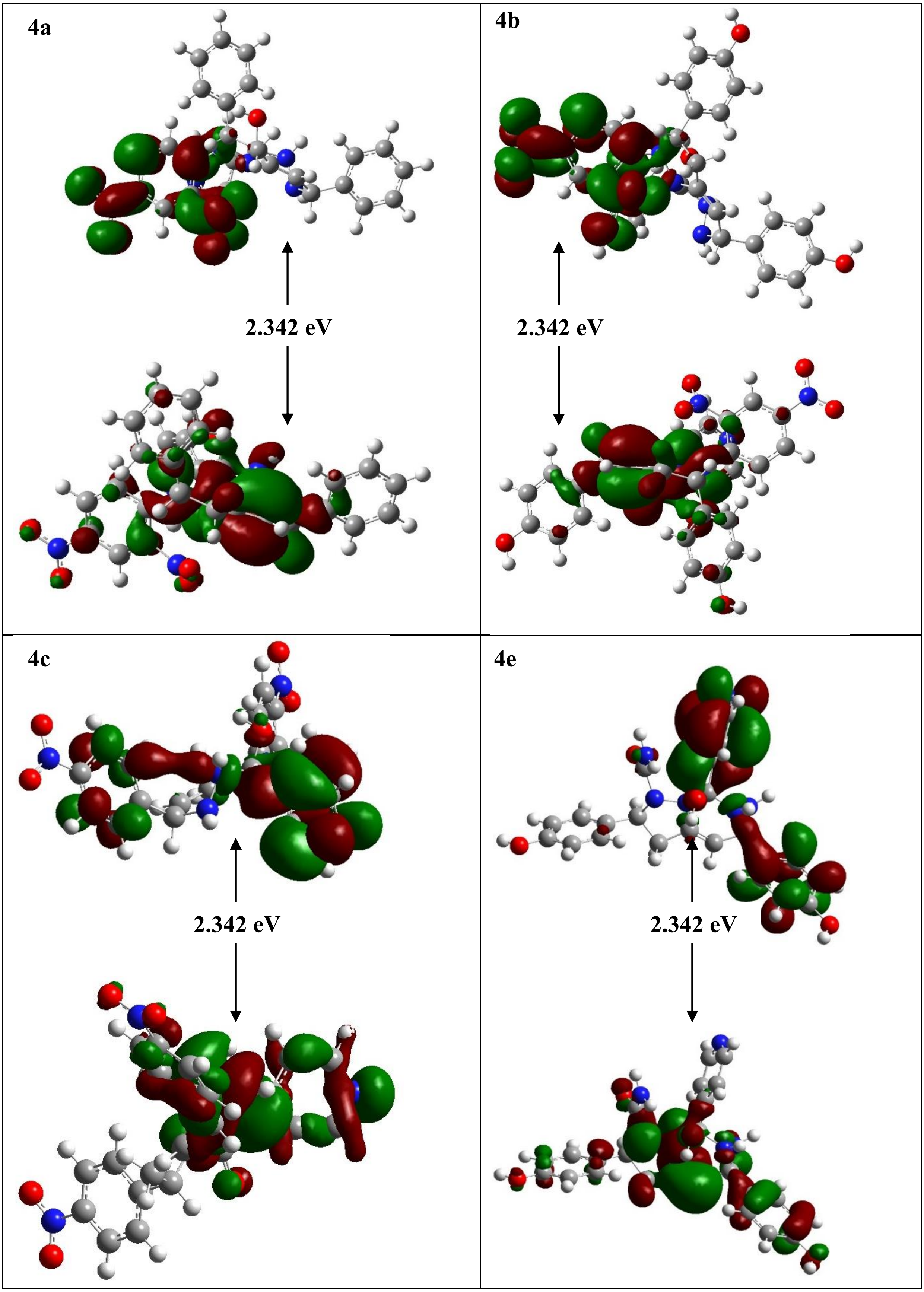

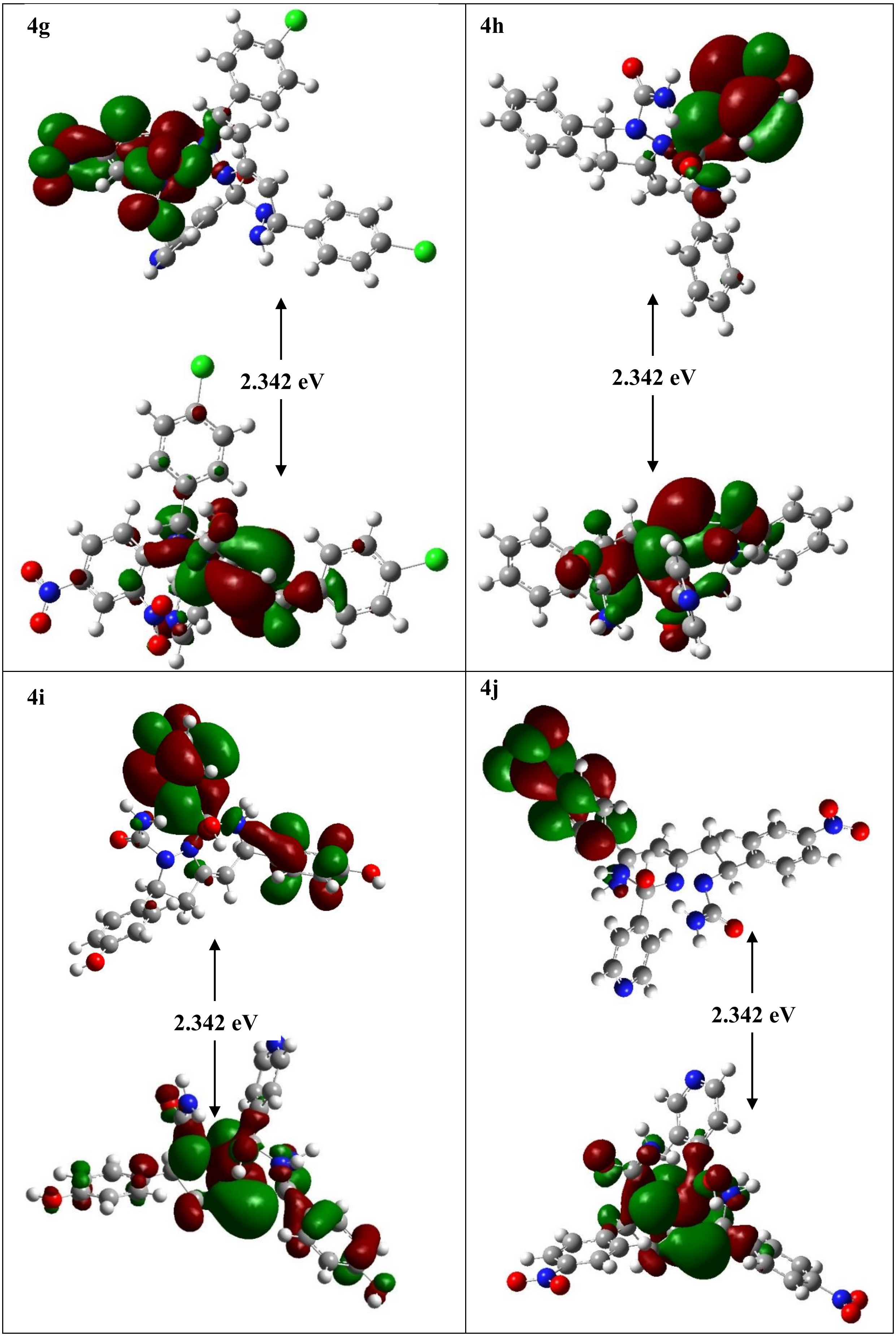
4g
2.342 eV
4h
2.342 eV
4i
2.342 eV
4j
2.342 eV

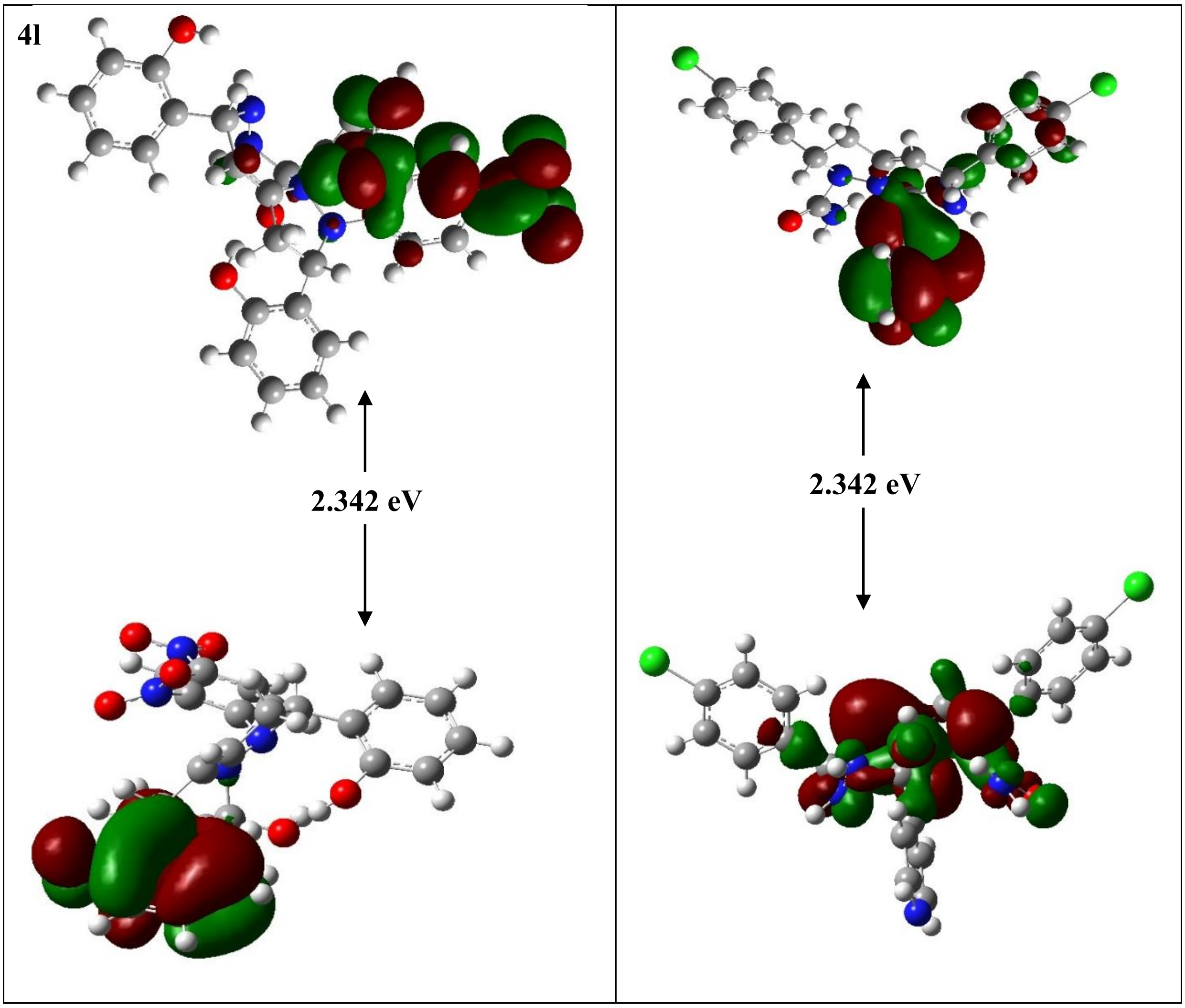


**Table S1.** Absorbance and corresponding % Inhibition of the tested compounds (4a-4n) against α-amylase enzyme at different concentrations.

| Compounds | Absorbance | | | % Inhibition (50 µg/ml) | | | Mean | Std. Dev. | % Inhibition (50 µg/ml) |
|---|---|---|---|---|---|---|---|---|---|
| | Set_1 | Set_2 | Set_3 | Set_1 | Set_2 | Set_3 | | | |
| 4a | 0.77 | 0.85 | 0.74 | 16.30435 | 20.65217 | 19.56522 | 18.84058 | 2.26268 | 18.84 ± 2.26 |
| 4b | 0.56 | 0.64 | 0.53 | 39.13043 | 34.78261 | 42.3913 | 38.76812 | 3.817266 | 39.13 ± 3.81 |
| 4c | 0.58 | 0.66 | 0.55 | 36.95652 | 32.6087 | 40.21739 | 36.5942 | 3.817266 | 36.59 ± 3.81 |
| 4d | 0.48 | 0.56 | 0.45 | 47.82609 | 45.65217 | 51.08696 | 48.18841 | 2.735447 | 48.18 ± 2.73 |
| 4e | 0.7 | 0.78 | 0.67 | 23.91304 | 23.91304 | 27.17391 | 25 | 1.882664 | 25.00 ± 1.88 |
| 4f | 0.51 | 0.59 | 0.48 | 44.56522 | 45.65217 | 47.82609 | 46.01449 | 1.660354 | 46.01 ± 1.66 |
| 4g | 0.74 | 0.78 | 0.7 | 19.56522 | 21.73913 | 23.91304 | 21.73913 | 2.173913 | 21.73 ± 2.17 |
| 4h | 0.78 | 0.82 | 0.74 | 15.21739 | 16.30435 | 19.56522 | 17.02899 | 2.26268 | 17.02 ± 2.26 |
| 4i | 0.64 | 0.68 | 0.6 | 30.43478 | 36.95652 | 34.78261 | 34.05797 | 3.320707 | 34.05 ± 3.32 |
| 4j | 0.68 | 0.72 | 0.64 | 26.08696 | 31.52174 | 30.43478 | 29.34783 | 2.875817 | 29.34 ± 2.87 |
| 4k | 0.6 | 0.64 | 0.56 | 34.78261 | 35.86957 | 39.13043 | 36.5942 | 2.26268 | 36.59 ± 2.26 |
| 4l | 0.71 | 0.77 | 0.67 | 22.82609 | 27.17391 | 27.17391 | 25.72464 | 2.510219 | 25.72 ± 2.51 |
| 4m | 0.62 | 0.68 | 0.58 | 32.6087 | 39.13043 | 36.95652 | 36.23188 | 3.320707 | 36.23 ± 3.32 |
| 4n | 0.75 | 0.81 | 0.71 | 18.47826 | 15.21739 | 22.82609 | 18.84058 | 3.817266 | 18.84 ± 3.81 |

| Compounds | Absorbance | | | % Inhibition (100 µg/ml) | | | Mean | Std. Dev. | % Inhibition (100 µg/ml) |
|---|---|---|---|---|---|---|---|---|---|
| | Set_1 | Set_2 | Set_3 | Set_1 | Set_2 | Set_3 | | | |
| 4a | 0.6 | 0.63 | 0.61 | 34.78261 | 36.95652 | 33.69565 | 35.14493 | 1.660354 | 35.14 ± 1.66 |
| 4b | 0.41 | 0.41 | 0.42 | 55.43478 | 55.43478 | 54.34783 | 55.07246 | 0.627555 | 55.07 ± 0.63 |
| 4c | 0.5 | 0.5 | 0.51 | 45.65217 | 45.65217 | 44.56522 | 45.28986 | 0.627555 | 45.29 ± 0.63 |
| 4d | 0.28 | 0.35 | 0.35 | 69.56522 | 72.82609 | 72.82609 | 71.73913 | 1.882664 | 71.74 ± 1.88 |
| 4e | 0.55 | 0.63 | 0.56 | 40.21739 | 42.3913 | 39.13043 | 40.57971 | 1.660354 | 40.58 ± 1.66 |
| 4f | 0.3 | 0.3 | 0.31 | 67.3913 | 67.3913 | 66.30435 | 67.02899 | 0.627555 | 67.03 ± 0.63 |
| 4g | 0.57 | 0.59 | 0.59 | 38.04348 | 35.86957 | 35.86957 | 36.5942 | 1.255109 | 36.59 ± 1.26 |
| 4h | 0.59 | 0.58 | 0.58 | 35.86957 | 36.95652 | 36.95652 | 36.5942 | 0.627555 | 36.59 ± 0.63 |
| 4i | 0.5 | 0.58 | 0.52 | 45.65217 | 47.82609 | 43.47826 | 45.65217 | 2.173913 | 45.65 ± 2.17 |
| 4j | 0.53 | 0.55 | 0.55 | 42.3913 | 40.21739 | 40.21739 | 40.94203 | 1.255109 | 40.94 ± 1.26 |
| 4k | 0.56 | 0.56 | 0.5 | 50 | 50 | 45.65217 | 48.55072 | 2.510219 | 48.55 ± 2.51 |
| 4l | 0.64 | 0.64 | 0.63 | 41.30435 | 41.30435 | 42.3913 | 41.66667 | 0.627555 | 41.67 ± 0.63 |
| 4m | 0.49 | 0.5 | 0.51 | 46.73913 | 45.65217 | 44.56522 | 45.65217 | 1.086957 | 45.65 ± 1.09 |
| 4n | 0.58 | 0.61 | 0.6 | 36.95652 | 33.69565 | 34.78261 | 35.14493 | 1.660354 | 35.14 ± 1.66 |

| Compounds | Absorbance | | | % Inhibition (200 µg/ml) | | | Mean | Std. Dev. | % Inhibition (200 µg/ml) |
|---|---|---|---|---|---|---|---|---|---|
| | Set_1 | Set_2 | Set_3 | Set_1 | Set_2 | Set_3 | | | |
| 4a | 0.58 | 0.58 | 0.57 | 36.95652 | 36.95652 | 38.04348 | 37.31884 | 0.627555 | 37.32 ± 0.63 |
| 4b | 0.38 | 0.37 | 0.37 | 58.69565 | 59.78261 | 59.78261 | 59.42029 | 0.627555 | 59.42 ± 0.63 |
| 4c | 0.43 | 0.44 | 0.42 | 53.26087 | 52.17391 | 54.34783 | 53.26087 | 1.086957 | 53.26 ± 1.09 |
| 4d | 0.2 | 0.2 | 0.19 | 78.26087 | 78.26087 | 79.34783 | 78.62319 | 0.627555 | 78.62 ± 0.63 |
| 4e | 0.48 | 0.46 | 0.47 | 47.82609 | 50 | 48.91304 | 48.91304 | 1.086957 | 48.91 ± 1.09 |
| 4f | 0.25 | 0.33 | 0.24 | 72.82609 | 75 | 73.91304 | 73.91304 | 1.086957 | 73.91 ± 1.09 |
| 4g | 0.48 | 0.47 | 0.46 | 47.82609 | 48.91304 | 50 | 48.91304 | 1.086957 | 48.91 ± 1.09 |
| 4h | 0.63 | 0.64 | 0.64 | 36.95652 | 44.56522 | 44.56522 | 42.02899 | 4.392882 | 42.03 ± 4.39 |
| 4i | 0.46 | 0.44 | 0.45 | 50 | 52.17391 | 51.08696 | 51.08696 | 1.086957 | 51.09 ± 1.09 |
| 4j | 0.49 | 0.48 | 0.48 | 46.73913 | 47.82609 | 47.82609 | 47.46377 | 0.627555 | 47.46 ± 0.63 |
| 4k | 0.42 | 0.43 | 0.41 | 54.34783 | 53.26087 | 55.43478 | 54.34783 | 1.086957 | 54.35 ± 1.09 |
| 4l | 0.5 | 0.49 | 0.49 | 45.65217 | 46.73913 | 46.73913 | 46.37681 | 0.627555 | 46.38 ± 0.63 |
| 4m | 0.48 | 0.47 | 0.49 | 52.17391 | 47.82609 | 48.91304 | 53.26087 | 2.26268 | 53.26 ± 2.26 |
| 4n | 0.51 | 0.51 | 0.5 | 44.56522 | 44.56522 | 45.65217 | 44.92754 | 0.627555 | 44.93 ± 0.63 |

| Compounds | Absorbance | | | % Inhibition (300 µg/ml) | | | Mean | Std. Dev. | % Inhibition (300 µg/ml) |
|---|---|---|---|---|---|---|---|---|---|
| | Set_1 | Set_2 | Set_3 | Set_1 | Set_2 | Set_3 | | | |
| 4a | 0.5 | 0.49 | 0.49 | 45.65217 | 46.73913 | 46.73913 | 46.37681 | 0.627555 | 46.38 ± 0.63 |
| 4b | 0.31 | 0.3 | 0.29 | 66.30435 | 67.3913 | 68.47826 | 67.3913 | 1.086957 | 67.39 ± 1.09 |
| 4c | 0.4 | 0.41 | 0.41 | 56.52174 | 55.43478 | 55.43478 | 55.7971 | 0.627555 | 55.80 ± 0.63 |
| 4d | 0.11 | 0.1 | 0.13 | 88.04348 | 89.13043 | 85.86957 | 87.68116 | 1.660354 | 87.68 ± 1.66 |
| 4e | 0.46 | 0.45 | 0.46 | 50 | 51.08696 | 50 | 50.36232 | 0.627555 | 50.36 ± 0.63 |
| 4f | 0.2 | 0.19 | 0.18 | 78.26087 | 79.34783 | 80.43478 | 79.34783 | 1.086957 | 79.35 ± 1.09 |
| 4g | 0.45 | 0.47 | 0.45 | 51.08696 | 48.91304 | 51.08696 | 50.36232 | 1.255109 | 50.36 ± 5.02 |
| 4h | 0.53 | 0.52 | 0.52 | 42.3913 | 43.47826 | 43.47826 | 43.11594 | 0.627555 | 43.12 ± 0.63 |
| 4i | 0.44 | 0.43 | 0.43 | 52.17391 | 53.26087 | 54.34783 | 53.26087 | 1.086957 | 53.26 ± 1.09 |

| | | | | | | | | | |
|---|---|---|---|---|---|---|---|---|---|
| 4j | 0.45 | 0.44 | 0.44 | 51.08696 | 52.17391 | 53.26087 | 53.26087 | 1.086957 | 53.26 ± 1.09 |
| 4k | 0.41 | 0.4 | 0.45 | 55.43478 | 56.52174 | 57.6087 | 56.52174 | 1.086957 | 56.52 ± 1.09 |
| 4l | 0.44 | 0.43 | 0.44 | 52.17391 | 53.26087 | 52.17391 | 52.53623 | 0.627555 | 52.54 ± 0.63 |
| 4m | 0.42 | 0.41 | 0.42 | 54.34783 | 55.43478 | 54.34783 | 54.71014 | 0.627555 | 54.71 ± 0.63 |
| 4n | 0.48 | 0.47 | 0.46 | 47.82609 | 48.91304 | 50 | 48.91304 | 1.086957 | 48.91 ± 1.09 |

| Compounds | Absorbance | | | % Inhibition (400 µg/ml) | | | Mean | Std. Dev. | % Inhibition (400 µg/ml) |
|---|---|---|---|---|---|---|---|---|---|
| | Set_1 | Set_2 | Set_3 | Set_1 | Set_2 | Set_3 | | | |
| 4a | 0.48 | 0.49 | 0.48 | 47.82609 | 46.73913 | 47.82609 | 47.46377 | 0.627555 | 47.46 ± 0.63 |
| 4b | 0.29 | 0.3 | 0.29 | 68.47826 | 67.3913 | 68.47826 | 68.11594 | 0.627555 | 68.12 ± 0.63 |
| 4c | 0.38 | 0.39 | 0.38 | 58.69565 | 57.6087 | 58.69565 | 58.33333 | 0.627555 | 58.33 ± 0.63 |
| 4d | 0.11 | 0.1 | 0.1 | 88.04348 | 89.13043 | 89.13043 | 88.76812 | 0.627555 | 88.77 ± 0.63 |
| 4e | 0.44 | 0.45 | 0.44 | 52.17391 | 51.08696 | 52.17391 | 51.81159 | 0.627555 | 51.81 ± 0.63 |
| 4f | 0.18 | 0.19 | 0.18 | 80.43478 | 79.34783 | 80.43478 | 80.07246 | 0.627555 | 80.07 ± 0.63 |
| 4g | 0.44 | 0.45 | 0.44 | 52.17391 | 51.08696 | 52.17391 | 51.81159 | 0.627555 | 51.81 ± 0.62 |
| 4h | 0.41 | 0.41 | 0.42 | 55.43478 | 55.43478 | 54.34783 | 55.07246 | 0.627555 | 55.07 ± 0.63 |
| 4i | 0.44 | 0.43 | 0.43 | 52.17391 | 53.26087 | 54.34783 | 53.26087 | 1.086957 | 53.26 ± 1.09 |
| 4j | 0.39 | 0.44 | 0.39 | 57.26087 | 52.17391 | 57.26087 | 55.56522 | 2.936956 | 52.90 ± 0.63 |
| 4k | 0.41 | 0.4 | 0.4 | 55.43478 | 56.52174 | 56.52174 | 56.15942 | 0.627555 | 56.16 ± 0.63 |
| 4l | 0.44 | 0.43 | 0.42 | 52.17391 | 53.26087 | 54.34783 | 53.26087 | 1.086957 | 53.26 ± 1.09 |
| 4m | 0.42 | 0.41 | 0.41 | 54.34783 | 55.43478 | 55.43478 | 55.07246 | 0.627555 | 55.07 ± 0.63 |
| 4n | 0.43 | 0.44 | - | 53.26087 | 52.17391 | - | 52.71739 | 0.768594 | 52.72 ± 0.77 |

| Compounds | Absorbance | | | % Inhibition (500 µg/ml) | | | Mean | Std. Dev. | % Inhibition (500µg/ml) |
|---|---|---|---|---|---|---|---|---|---|
| | Set_1 | Set_2 | Set_3 | Set_1 | Set_2 | Set_3 | | | |
| 4a | 0.44 | 0.43 | 0.43 | 52.17391 | 54.34783 | 54.34783 | 53.62319 | 1.255109 | 53.62 ± 1.25 |
| 4b | 0.29 | 0.3 | 0.29 | 68.47826 | 67.3913 | 68.47826 | 68.11594 | 0.627555 | 68.11 ± 0.62 |
| 4c | 0.38 | 0.36 | 0.36 | 58.69565 | 60.86957 | 60.86957 | 60.14493 | 1.255109 | 60.14 ± 1.25 |
| 4d | 0.1 | 0.1 | 0.1 | 89.13043 | 89.13043 | 89.13043 | 89.13043 | 0 | 89.13 ± 0.00 |
| 4e | 0.42 | 0.42 | 0.44 | 52.17391 | 54.34783 | 54.34783 | 53.62319 | 1.255109 | 53.62 ± 1.25 |
| 4f | 0.18 | 0.18 | 0.18 | 80.43478 | 80.43478 | 80.43478 | 80.43478 | 0 | 80.43 ± 0.00 |
| 4g | 0.53 | 0.54 | 0.54 | 53.26087 | 52.17391 | 52.17391 | 52.53623 | 0.627555 | 52.54 ± 0.63 |
| 4h | 0.41 | 0.41 | 0.4 | 55.43478 | 55.43478 | 56.52174 | 55.7971 | 0.627555 | 55.79 ± 0.62 |
| 4i | 0.43 | 0.43 | 0.41 | 53.26087 | 53.26087 | 55.43478 | 53.98551 | 1.255109 | 53.98 ± 1.25 |
| 4j | 0.39 | 0.38 | 0.4 | 57.26087 | 58.69565 | 56.52174 | 57.49275 | 1.105352 | 57.49 ± 1.10 |
| 4k | 0.39 | 0.38 | 0.38 | 57.26087 | 58.69565 | 58.69565 | 58.21739 | 0.828372 | 58.21 ± 0.82 |
| 4l | 0.42 | 0.43 | 0.42 | 54.34783 | 53.26087 | 54.34783 | 53.98551 | 0.627555 | 53.98 ± 0.62 |
| 4m | 0.4 | 0.41 | 0.41 | 56.52174 | 55.43478 | 55.43478 | 55.7971 | 0.627555 | 55.79 ± 0.62 |
| 4n | 0.43 | 0.42 | - | 53.26087 | 54.34783 | - | 53.80435 | 0.768594 | 53.80 ± 0.76 |

**Table S2.** Absorbance and corresponding % Inhibition of the tested compounds (4a-4n) against α-glucosidase enzyme at different concentrations.

| Compounds | Absorbance | | | % Inhibition (50 µg/ml) | | | Mean | Std. Dev. | % Inhibition (50 µg/ml) |
|---|---|---|---|---|---|---|---|---|---|
| | Set_1 | Set_2 | Set_3 | Set_1 | Set_2 | Set_3 | | | |
| 4a | - | - | - | - | - | - | - | - | - |
| 4b | 0.62 | 0.63 | 0.61 | 20.51282 | 19.23077 | 21.79487 | 20.51282 | 1.282051 | 20.51 ± 1.28 |
| 4c | 0.66 | 0.65 | 0.67 | 15.38462 | 16.66667 | 14.10256 | 15.38462 | 1.282051 | 15.38 ± 1.28 |
| 4d | 0.5 | 0.51 | 0.51 | 35.89744 | 34.61538 | 34.61538 | 35.04274 | 0.740193 | 35.04 ± 0.74 |
| 4e | 0.68 | 0.68 | 0.67 | 12.82051 | 12.82051 | 14.10256 | 13.24786 | 0.740193 | 13.25 ± 0.74 |
| 4f | 0.52 | 0.52 | 0.51 | 33.33333 | 33.33333 | 34.61538 | 33.76068 | 0.740193 | 33.76 ± 0.74 |
| 4g | 0.58 | 0.57 | 0.57 | 25.64103 | 26.92308 | 26.92308 | 26.49573 | 0.740193 | 26.50 ± 0.74 |
| 4h | - | - | - | - | - | - | - | - | - |
| 4i | 0.54 | 0.53 | 0.55 | 30.76923 | 32.05128 | 29.48718 | 30.76923 | 1.282051 | 30.77 ± 1.28 |
| 4j | 0.57 | 0.56 | 0.57 | 26.92308 | 28.20513 | 26.92308 | 27.35043 | 0.740193 | 27.35 ± 0.74 |
| 4k | 0.56 | 0.55 | 0.56 | 28.20513 | 29.48718 | 28.20513 | 28.63248 | 0.740193 | 28.63 ± 0.74 |
| 4l | 0.69 | 0.68 | 0.69 | 11.53846 | 12.82051 | 11.53846 | 11.96581 | 0.740193 | 11.97 ± 0.74 |
| 4m | 0.6 | 0.61 | 0.6 | 23.07692 | 21.79487 | 23.07692 | 22.64957 | 0.740193 | 22.65 ± 0.74 |
| 4n | 0.65 | 0.64 | 0.65 | 16.66667 | 17.94872 | 16.66667 | 17.09402 | 0.740193 | 17.09 ± 0.74 |

| Compounds | Absorbance | | | % Inhibition (100 µg/ml) | | | Mean | Std. Dev. | % Inhibition (100 µg/ml) |
|---|---|---|---|---|---|---|---|---|---|
| | Set_1 | Set_2 | Set_3 | Set_1 | Set_2 | Set_3 | | | |
| 4a | - | - | - | - | - | - | - | - | - |
| 4b | 0.52 | 0.54 | 0.51 | 33.33333 | 30.76923 | 34.61538 | 32.90598 | 1.958366 | 32.91 ± 1.96 |
| 4c | 0.56 | 0.57 | 0.54 | 28.20513 | 26.92308 | 30.76923 | 28.63248 | 1.958366 | 28.63 ± 1.96 |
| 4d | 0.43 | 0.41 | 0.44 | 44.87179 | 47.4359 | 43.58974 | 45.29915 | 1.958366 | 45.30 ± 1.96 |
| 4e | 0.66 | 0.63 | 0.67 | 15.38462 | 19.23077 | 14.10256 | 16.23932 | 2.668803 | 16.24 ± 2.67 |
| 4f | 0.49 | 0.47 | 0.47 | 37.17949 | 39.74359 | 39.74359 | 38.88889 | 1.480385 | 38.89 ± 1.48 |
| 4g | 0.51 | 0.52 | 0.53 | 34.61538 | 33.33333 | 32.05128 | 33.33333 | 1.282051 | 33.33 ± 1.28 |
| 4h | - | - | - | - | - | - | - | - | - |
| 4i | 0.5 | 0.53 | 0.51 | 35.89744 | 32.05128 | 34.61538 | 34.18803 | 1.958366 | 34.19 ± 1.96 |
| 4j | 0.58 | 0.56 | 0.55 | 25.64103 | 28.20513 | 29.48718 | 27.77778 | 1.958366 | 27.78 ± 1.96 |
| 4k | 0.52 | 0.54 | 0.54 | 33.33333 | 30.76923 | 30.76923 | 31.62393 | 1.480385 | 31.62 ± 1.48 |
| 4l | 0.63 | 0.62 | 0.64 | 19.23077 | 20.51282 | 17.94872 | 19.23077 | 1.282051 | 19.23 ± 1.28 |
| 4m | 0.56 | 0.55 | 0.57 | 28.20513 | 29.48718 | 26.92308 | 28.20513 | 1.282051 | 28.21 ± 1.28 |
| 4n | 0.61 | 0.63 | 0.64 | 21.79487 | 19.23077 | 17.94872 | 19.65812 | 1.958366 | 19.66 ± 1.96 |

| Compounds | Absorbance | | | % Inhibition (200 µg/ml) | | | Mean | Std. Dev. | % Inhibition (200 µg/ml) |
|---|---|---|---|---|---|---|---|---|---|
| | Set_1 | Set_2 | Set_3 | Set_1 | Set_2 | Set_3 | | | |
| 4a | 0.66 | 0.65 | 0.64 | 15.38462 | 16.66667 | 17.94872 | 16.66667 | 1.813094 | 16.67 ± 1.81 |
| 4b | 0.48 | 0.47 | 0.45 | 38.46154 | 39.74359 | 42.30769 | 40.17094 | 23.03851 | 40.17 ± 23.04 |
| 4c | 0.5 | 0.55 | 0.52 | 35.89744 | 29.48718 | 33.33333 | 32.90598 | 19.64603 | 32.91 ± 19.65 |
| 4d | 0.36 | 0.38 | 0.34 | 53.84615 | 51.28205 | 56.41026 | 53.84615 | 31.56566 | 53.85 ± 31.57 |
| 4e | 0.59 | 0.57 | 0.55 | 24.35897 | 26.92308 | 29.48718 | 26.92308 | 15.36688 | 26.92 ± 15.37 |
| 4f | 0.4 | 0.42 | 0.43 | 48.71795 | 46.15385 | 44.87179 | 46.5812 | 26.78593 | 46.58 ± 26.79 |
| 4g | - | - | - | - | - | - | - | - | - |
| 4h | 0.62 | 0.61 | 0.61 | 20.51282 | 21.79487 | 21.79487 | 21.36752 | 0.906547 | 21.37 ± 0.91 |
| 4i | - | - | - | - | - | - | - | - | - |

| | | | | | | | | | |
|---|---|---|---|---|---|---|---|---|---|
| 4j | 0.53 | 0.54 | 0.53 | 32.05128 | 30.76923 | 32.05128 | 31.62393 | 18.17573 | 17.09 ± 9.13 |
| 4k | 0.43 | 0.41 | 0.41 | 44.87179 | 47.4359 | 47.4359 | 46.5812 | 26.35482 | 46.58 ± 26.35 |
| 4l | 0.56 | 0.55 | 0.53 | 28.20513 | 29.48718 | 32.05128 | 29.91453 | 17.10461 | 29.91 ± 17.10 |
| 4m | 0.46 | 0.43 | 0.43 | 41.02564 | 44.87179 | 44.87179 | 43.58974 | 24.52556 | 43.59 ± 24.53 |
| 4n | 0.53 | 0.54 | 0.54 | 32.05128 | 30.76923 | 30.76923 | 31.19658 | 17.77101 | 31.20 ± 17.77 |

| Compounds | Absorbance | | | % Inhibition (300 µg/ml) | | | Mean | Std. Dev. | % Inhibition (300 µg/ml) |
|---|---|---|---|---|---|---|---|---|---|
| | Set_1 | Set_2 | Set_3 | Set_1 | Set_2 | Set_3 | | | |
| 4a | 0.54 | 0.53 | 0.53 | 30.76923 | 32.05128 | 32.05128 | 31.62393 | 0.740193 | 31.62 ± 0.74 |
| 4b | 0.43 | 0.42 | 0.41 | 44.87179 | 46.15385 | 47.4359 | 46.15385 | 1.282051 | 46.15 ± 1.28 |
| 4c | 0.45 | 0.47 | 0.46 | 42.30769 | 39.74359 | 41.02564 | 41.02564 | 1.282051 | 41.03 ± 1.28 |
| 4d | 0.28 | 0.27 | 0.25 | 64.10256 | 65.38462 | 67.94872 | 65.81197 | 1.958366 | 65.81 ± 1.96 |
| 4e | 0.52 | 0.51 | 0.54 | 33.33333 | 34.61538 | 30.76923 | 32.90598 | 1.958366 | 32.91 ± 1.96 |
| 4f | 0.38 | 0.37 | 0.37 | 51.28205 | 52.5641 | 52.5641 | 52.13675 | 0.740193 | 52.14 ± 0.74 |
| 4g | 0.5 | 0.51 | 0.52 | 35.89744 | 34.61538 | 33.33333 | 34.61538 | 1.282051 | 34.62 ± 1.28 |
| 4h | 0.51 | 0.52 | 0.51 | 34.61538 | 33.33333 | 34.61538 | 34.18803 | 0.740193 | 34.19 ± 0.74 |
| 4i | 0.44 | 0.43 | 0.46 | 43.58974 | 44.87179 | 41.02564 | 43.16239 | 1.958366 | 43.16 ± 1.96 |
| 4j | 0.52 | 0.52 | 0.54 | 33.33333 | 33.33333 | 30.76923 | 32.47863 | 1.480385 | 32.48 ± 1.48 |
| 4k | 0.4 | 0.42 | 0.41 | 48.71795 | 46.15385 | 47.4359 | 47.4359 | 1.282051 | 47.44 ± 1.28 |
| 4l | 0.48 | 0.46 | 0.46 | 38.46154 | 41.02564 | 41.02564 | 40.17094 | 1.480385 | 40.17 ± 1.48 |
| 4m | 0.42 | 0.42 | 0.43 | 46.15385 | 46.15385 | 44.87179 | 45.7265 | 0.740193 | 45.73 ± 0.74 |
| 4n | 0.46 | 0.48 | 0.44 | 41.02564 | 38.46154 | 43.58974 | 41.02564 | 2.564103 | 41.03 ± 2.56 |

| Compounds | Absorbance | | | % Inhibition (400 µg/ml) | | | Mean | Std. Dev. | % Inhibition (400 µg/ml) |
|---|---|---|---|---|---|---|---|---|---|
| | Set_1 | Set_2 | Set_3 | Set_1 | Set_2 | Set_3 | | | |
| 4a | 0.43 | 0.54 | 0.53 | 44.87179 | 30.76923 | 32.05128 | 35.89744 | 7.798414 | 35.90 ± 7.80 |
| 4b | 0.39 | 0.37 | 0.37 | 50 | 52.5641 | 52.5641 | 51.7094 | 1.480385 | 51.71 ± 1.48 |
| 4c | 0.43 | 0.42 | 0.41 | 44.87179 | 46.15385 | 47.4359 | 46.15385 | 1.282051 | 46.15 ± 1.28 |
| 4d | 0.22 | 0.24 | 0.21 | 71.79487 | 69.23077 | 73.07692 | 71.36752 | 1.958366 | 71.37 ± 1.96 |
| 4e | 0.49 | 0.47 | 0.47 | 37.17949 | 39.74359 | 39.74359 | 38.88889 | 1.480385 | 38.89 ± 1.48 |
| 4f | 0.24 | 0.22 | 0.28 | 69.23077 | 71.79487 | 64.10256 | 68.37607 | 3.916731 | 68.38 ± 3.92 |
| 4g | 0.39 | 0.37 | 0.37 | 50 | 52.5641 | 52.5641 | 51.7094 | 1.480385 | 51.71 ± 1.48 |
| 4h | 0.48 | 0.46 | 0.46 | 38.46154 | 41.02564 | 41.02564 | 40.17094 | 1.480385 | 40.17 ± 1.48 |
| 4i | 0.41 | 0.39 | 0.43 | 47.4359 | 50 | 44.87179 | 47.4359 | 2.564103 | 47.44 ± 2.56 |
| 4j | 0.48 | 0.46 | 0.43 | 38.46154 | 41.02564 | 44.87179 | 41.45299 | 3.226425 | 41.45 ± 3.23 |
| 4k | 0.3 | 0.28 | 0.32 | 61.53846 | 64.10256 | 58.97436 | 61.53846 | 2.564103 | 61.54 ± 2.56 |
| 4l | 0.46 | 0.44 | 0.44 | 41.02564 | 43.58974 | 43.58974 | 42.73504 | 1.480385 | 42.74 ± 1.48 |
| 4m | 0.34 | 0.32 | 0.35 | 56.41026 | 58.97436 | 55.12821 | 56.83761 | 1.958366 | 56.84 ± 1.96 |
| 4n | 0.49 | 0.47 | 0.48 | 37.17949 | 39.74359 | 38.46154 | 38.46154 | 1.282051 | 38.46 ± 1.28 |

| Compounds | Absorbance | | | % Inhibition (500 µg/ml) | | | Mean | Std. Dev. | % Inhibition (500µg/ml) |
|---|---|---|---|---|---|---|---|---|---|
| | Set_1 | Set_2 | Set_3 | Set_1 | Set_2 | Set_3 | | | |
| 4a | 0.39 | 0.38 | 0.39 | 50 | 51.28205 | 50 | 50.42735 | 28.55574 | 50.43 ± 28.56 |
| 4b | 0.32 | 0.31 | 0.32 | 58.97436 | 60.25641 | 58.97436 | 59.40171 | 33.8006 | 59.40 ± 33.80 |

| | | | | | | | | | |
|---|---|---|---|---|---|---|---|---|---|
| 4c | 0.4 | 0.4 | 0.4 | 48.71795 | 48.71795 | 48.71795 | 48.71795 | 27.86751 | 48.72 ± 27.87 |
| 4d | 0.11 | 0.1 | 0.1 | 85.89744 | 87.17949 | 87.17949 | 86.75214 | 49.80547 | 86.75 ± 49.81 |
| 4e | 0.42 | 0.41 | 0.42 | 46.15385 | 47.4359 | 46.15385 | 46.5812 | 26.34671 | 46.58 ± 26.35 |
| 4f | 0.19 | 0.18 | 0.18 | 75.64103 | 76.92308 | 76.92308 | 76.49573 | 43.82676 | 76.50 ± 43.83 |
| 4g | 0.31 | 0.31 | 0.31 | 60.25641 | 60.25641 | 60.25641 | 60.25641 | 34.50038 | 60.26 ± 34.50 |
| 4h | 0.42 | 0.43 | 0.42 | 46.15385 | 44.87179 | 46.15385 | 45.7265 | 26.35249 | 45.73 ± 26.35 |
| 4i | 0.39 | 0.38 | 0.38 | 50 | 51.28205 | 51.28205 | 50.8547 | 28.99066 | 50.85 ± 28.99 |
| 4j | 0.43 | 0.42 | 0.43 | 44.87179 | 46.15385 | 44.87179 | 45.29915 | 25.60652 | 45.30 ± 25.61 |
| 4k | 0.24 | 0.23 | 0.24 | 69.23077 | 70.51282 | 69.23077 | 69.65812 | 39.73946 | 69.66 ± 39.74 |
| 4l | 0.41 | 0.43 | 0.41 | 47.4359 | 44.87179 | 47.4359 | 46.5812 | 27.11 | 46.58 ± 27.11 |
| 4m | 0.28 | 0.28 | 0.28 | 64.10256 | 64.10256 | 64.10256 | 64.10256 | 36.76715 | 64.10 ± 36.77 |
| 4n | 0.44 | 0.43 | 0.43 | 43.58974 | 44.87179 | 44.87179 | 44.44444 | 25.27919 | 44.44 ± 25.28 |
